# Identification of key opportunities for optimising the management of high-risk COPD patients in the UK using the CONQUEST quality standards: an observational longitudinal study

**DOI:** 10.1016/j.lanepe.2023.100619

**Published:** 2023-04-21

**Authors:** David M.G. Halpin, Andrew P. Dickens, Derek Skinner, Ruth Murray, Mukesh Singh, Katherine Hickman, Victoria Carter, Amy Couper, Alexander Evans, Rachel Pullen, Shruti Menon, Tamsin Morris, Hana Muellerova, Mona Bafadhel, James Chalmers, Graham Devereux, Martin Gibson, John R. Hurst, Rupert Jones, Konstantinos Kostikas, Jennifer Quint, Dave Singh, Marije van Melle, Tom Wilkinson, David Price

**Affiliations:** a University of Exeter Medical School, College of Medicine and Health, University of Exeter, Exeter, UK; b Observational and Pragmatic Research Institute, Singapore; c Optimum Patient Care, UK; d General Practice, Horse Fair Practice Group, Rugeley, Staffordshire, UK; e Keele University Medical School, Keele, UK; f Staffordshire Integrated Care System, UK; g National Asthma and COPD Audit Programme, Care Quality Improvement Department (CQID), RCP, London, UK; h Low Moor Medical Practice, Bradford, UK; i Leeds and Bradford Clinical Commissioning Groups, UK; j Medical and Scientific Affairs, AstraZeneca, London, UK; k BioPharmaceuticals Medical, AstraZeneca, Cambridge, UK; l School of Immunology & Microbial Science, King's College, London, UK; m Scottish Centre for Respiratory Research, University of Dundee, Ninewells Hospital and Medical School, Dundee, UK; n Liverpool School of Tropical Medicine, Liverpool, UK; o Salford Royal NHS Foundation Trust & Chief Executive Officer of NorthWest EHealth, UK; p UCL Respiratory, University College London, London, UK; q Plymouth Marjon University, Plymouth, UK; r Respiratory Medicine Department, University of Ioannina, Greece; s Respiratory Epidemiology, Faculty of Medicine, National Heart & Lung Institute, London, UK; t Division of Infection, Immunity & Respiratory Medicine, University of Manchester, Manchester, UK; u Connecting Medical Dots BV, Utrecht, the Netherlands; v ORTEC, Zoetermeer, the Netherlands; w University of Southampton Faculty of Medicine, Southampton, UK; x National Institute for Health and Care Research Southampton Biomedical Research Centre, Southampton, UK; y Centre of Academic Primary Care, Division of Applied Health Sciences, University of Aberdeen, Aberdeen, UK

**Keywords:** COPD, Diagnosis, Spirometry, Exacerbations, Treatment, Pulmonary rehabilitation

## Abstract

**Background:**

This study compared management of high-risk COPD patients in the UK to national and international management recommendations and quality standards, including the COllaboratioN on QUality improvement initiative for achieving Excellence in STandards of COPD care (CONQUEST). The primary comparison was in 2019, but trends from 2000 to 2019 were also examined.

**Methods:**

Patients identified in the Optimum Patient Care Research Database were categorised as newly diagnosed (≤12 months after diagnosis), already diagnosed, and potential COPD (smokers having exacerbation-like events). High-risk patients had a history of ≥2 moderate or ≥1 severe exacerbations in the previous 12 months.

**Findings:**

For diagnosed patients, the median time between diagnosis and first meeting the high-risk criteria was 617 days (Q1-Q3: 3246). The use of spirometry for diagnosis increased dramatically after 2004 before plateauing and falling in recent years. In 2019, 41% (95% CI 39–44%; n = 550/1343) of newly diagnosed patients had no record of spirometry in the previous year, and 45% (95% CI 43–48%; n = 352/783) had no record of a COPD medication review within 6 months of treatment initiation or change. In 2019, 39% (n = 6893/17,858) of already diagnosed patients had no consideration of exacerbation rates, 46% (95% CI 45–47%; n = 4942/10,725) were not offered or referred for pulmonary rehabilitation, and 41% (95% CI 40–42%; n = 3026/7361) had not had a COPD review within 6 weeks of respiratory hospitalization.

**Interpretation:**

Opportunities for early diagnosis of COPD patients at high risk of exacerbations are being missed. Newly and already diagnosed patients at high-risk are not being assessed or treated promptly. There is substantial scope to improve the assessment and treatment optimisation of these patients.

**Funding:**

This study is conducted by the Observational & Pragmatic Research International Ltd and was co-funded by Optimum Patient Care and AstraZeneca. No funding was received by the Observational & Pragmatic Research Institute Pte Ltd (OPRI) for its contribution.

**Research in context**

**Evidence before this study**

The UK has one of the worst age-standardized years of life lost from COPD in Europe, and there has been no change in mortality rates over the last 10 years. Previous studies have shown that key opportunities to optimize the management of COPD in primary care are being missed as a result of late diagnosis, variable adherence to evidence-based management guidelines, delayed implementation of appropriate interventions, and under-treatment. Recently The COllaboratioN on QUality improvement initiative for achieving Excellence in STandards of COPD care (CONQUEST) developed a roadmap to improved quality of COPD care based on four quality standards (QS). A PubMed search was completed using the following search fields: 1) COPD or Chronic Obstructive Pulmonary Disease (all fields) AND 2) Clinical Trials or Meta-Analysis (all fields) OR 3) articles in the top 20 medical or respiratory journals (available on request) or The Cochrane Database of Systematic Reviews.

**Added value of this study**

Our study includes a large number of patients, and reports data from across the UK, as well as trends over time. We concentrated on the management of high-risk patients, i.e., those who had had ≥2 moderate or ≥1 severe (requiring hospitalization) exacerbations in the previous 12 months. The results highlight the continuing differences between recommendations and QS for COPD and real-world management of these high-risk patients in the UK, as well as examining changes over time. The data will allow clinicians and payors to identify opportunities for improvement.

**Implications of all the available evidence**

Our findings show that opportunities for timely diagnosis of patients with COPD at high risk of exacerbating are being missed. Newly and already diagnosed high-risk patients are still not being assessed or treated promptly, and there is substantial scope to improve assessment of these patients and optimize their treatment.

## Introduction

Despite having universal free health care, comprehensive primary care and hospital services, access to effective therapies, and national guidelines and quality standards (QS) supported by implementation initiatives, the burden of chronic obstructive pulmonary disease (COPD) in the UK remains high.^1^ The UK has one of the worst age-standardised years of life lost from COPD in Europe, with no change in this position between 1990 and 2010,^2^ and there has been no change in mortality rates over the last 10 years.^3^ COPD is an important determinant of current inequalities in health outcomes across the UK.^4^

Key opportunities to optimize the management of COPD in primary care are being missed, often as a result of late diagnosis, variable adherence to evidence-based management guidelines/strategy documents, delayed implementation of appropriate interventions, and under-recognition of patients at higher risk of adverse outcomes.^5^^,^^6^ Delayed diagnosis and under-treatment of COPD results in a significantly higher risk of exacerbations and hospitalisation,^7^ which are themselves associated with poor long-term outcomes and death. Comorbidities, particularly cardiac disease, are associated with increased mortality in COPD^8^ and assessment and management of these conditions is also essential to improve long term outcomes.

A decade ago, the then-Department of Health, now Department of Health and Social Care (DHSC), published an outcomes strategy for COPD that contained a set of measures intended to minimize inequalities, reduce mortality, and ensure those with COPD received safe and effective care.^9^ However, outcomes remain suboptimal, and the 2019 NHS long-term plan has again emphasised the need for improvements in COPD management. It recommended changes in the models of COPD care, with a focus on community care and collaborative and integrated working.^10^ Improving COPD management has also been identified as a priority by the Welsh and Scottish Governments.^11^^,^^12^

The COllaboratioN on QUality improvement initiative for achieving Excellence in STandards of COPD care (CONQUEST) is a new, multi-national program providing a roadmap to improved quality of COPD care based on four QS.^13^^,^^14^ The aim of this study is to describe how current UK management of high-risk patients with COPD compares to the CONQUEST QS, to examine how this evolved between 2000 and 2019, and to identify opportunities for improvement.

## Methods

### Study design

**Fig. 1:**
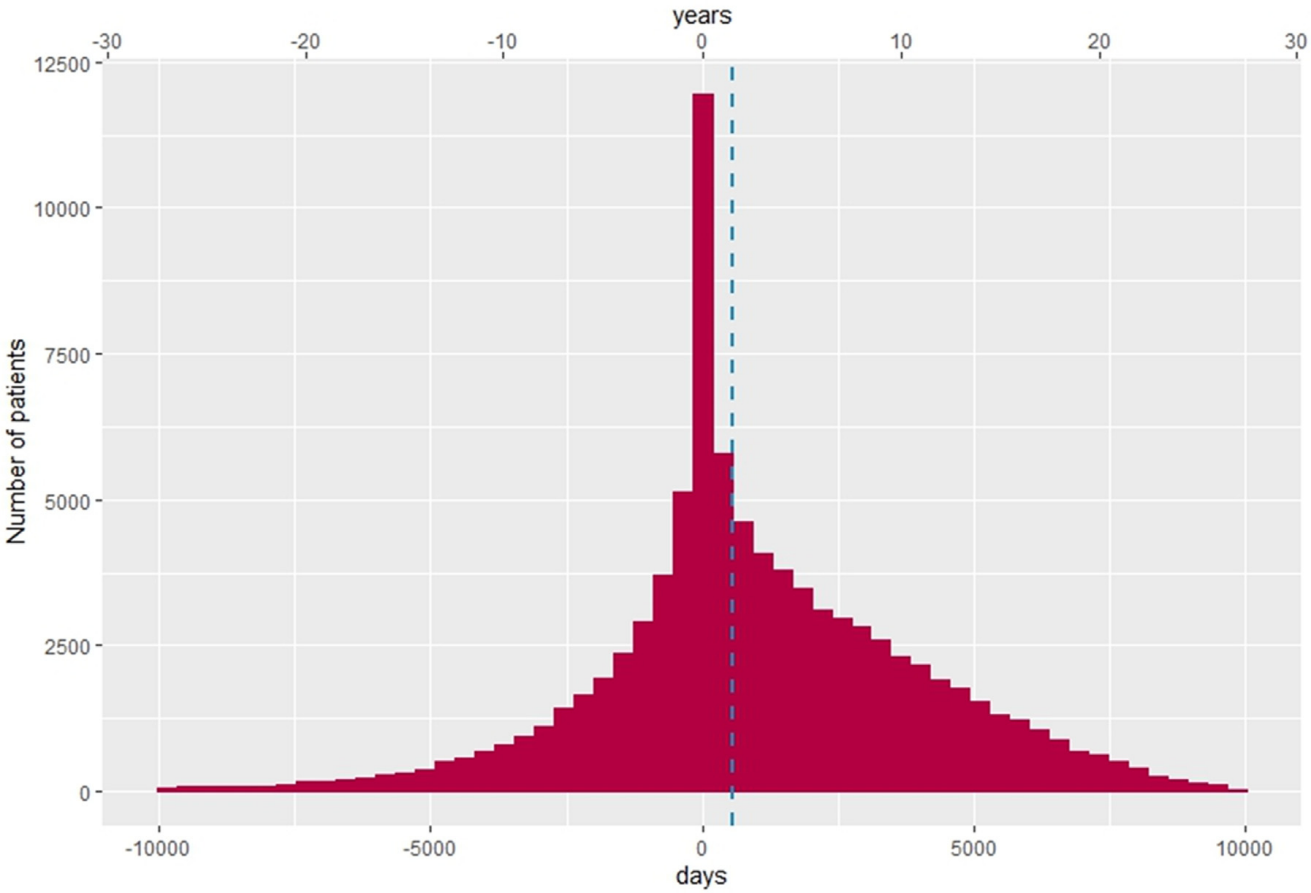
Time between COPD diagnosis and fist meeting high-risk criteria. Data are expressed as a histogram showing number of patients on the Y-axis and time (days) between diagnosis and high risk on the X-axis. Blue dotted line = median time. See Table 1 for definitions of high risk. N numbers for each patient cohort are provided in S-Table 3**.** Analysis of spirometry data was based on valid entries in any of the following indicators: FEV_1_, FVC, FEV_1_/FVC. Eligible patient: patients aged ≥40 years with a COPD diagnosis, and those who did not have a COPD diagnosis, but had a history of smoking and COPD-like exacerbations. High-risk patients were those with ≥2 moderate or ≥1 severe (hospitalised) exacerbations in the last 12 months.

This observational, longitudinal, descriptive study included a population of high-risk patients with diagnosed or potential COPD (Table 1) registered at general practitioner (GP) practices. The analysis sample was identified in 2019 and in each previous year back to 2000, with routinely collected primary care data being assessed over the relevant time frame for each outcome; the 12 months before or after 1 January of each study year (S-Fig. 1). 2019 was chosen as the key year for the data as it provides the most up-to-date information prior to the COVID-19 pandemic. The study protocol was established prior to data extraction, in accordance with the criteria for the European Network Centers for Pharmacoepidemiology and Pharmacovigilance (ENCePP) and is registered with the European Network of Centers for Pharmacoepidemiology and Pharmacovigilance (ENCEPP/DSPP/42512).^15^ As noted, the dataset supporting the conclusions of this article was derived from the OPCRD. The OPCRD has ethical approval from the National Health Service (NHS) Research Authority to hold and process anonymised research data (Research Ethics Committee reference: 15/EM/0150). Registration of the study with the European Union electronic Register of Post-Authorization studies was also undertaken (EUPAS43721). Approval for this study was granted by the Anonymised Data Ethics Protocols and Transparency (ADEPT) committee – the independent scientific advisory committee for the OPCRD (ADEPT0221).

**Table 1:** Exacerbation and patient cohort definitions.

Term	Definition
COPD exacerbation	A significant worsening in respiratory symptoms in people with COPD (or an event analogous to a COPD exacerbation in people with suspected but undiagnosed COPD), categorized as moderate or severe using validated code lists.^34^ Exacerbations occurring a minimum of 14 days from start of treatment of the initial exacerbation were considered as separate exacerbations. Prescribing instructions held in EMRs were used to identify the end of an exacerbation episode (i.e., using recorded information on the duration of the treatment).
Moderate exacerbation	Required a prescription of OCS and/or a course of antibiotics within 3 days of a lower respiratory consultation, or a hospital attendance
Severe exacerbation	An exacerbation resulting in a respiratory-related hospitalisation or death.
High-risk patients with COPD	Patients with COPD (or potential COPD) who have had 2 or more moderate, or 1 or more severe exacerbations in the last 12 months.
Newly diagnosed high-risk patients with COPD	Those with a first recorded COPD diagnosis within the 12-month period prior to 1 January for each study year, who fit the high-risk criteria with respect to recent exacerbations of potential COPD.
Already diagnosed high-risk patients with COPD	Those diagnosed with COPD at any point in their history before the 12-month period prior to 1 January of each study year, who fit the high-risk criteria with respect to recent exacerbations of COPD.
Potential undiagnosed high-risk patients with COPD	Those without a COPD diagnosis (ever) in their EMR prior to 1 January of each study year, who fit the high-risk criteria with respect to recent exacerbations of potential COPD, and who are current or ex-smokers with either 10 years smoking duration or 10 pack-years.
Initiation/step-up to maintenance therapy	Initiation or step-up to new pharmacological treatment in response to recent exacerbations

COPD: chronic obstructive pulmonary disease; EMR: electronic medical record; OCS: oral corticosteroids

### Data source

Data were obtained from patient electronic medical records (EMRs) using the Optimum Patient Care Research Database (OPCRD).^16^ The OPCRD, established in 2005, comprises EMRs of more than 15 million patients (approximately 8% of the total UK population) and contains regularly inputted data from 1988 and retrospectively inputted data from 1950.^16^ See online supplement for additional information on data capture. It is approved by the UK National Health Service for clinical research use,^17^ is well-validated, and is used frequently for health research.^18^

### Patients

The eligible population included patients aged ≥40 years who were categorised into 3 cohorts (Table 1):

(i) newly diagnosed COPD; the first record of a COPD diagnosis occurred within the 12-month baseline period beginning the 1st of January.
(ii) already diagnosed COPD: the diagnosis had been made at any point prior to the 12-month baseline period before the 1st of January.
(iii) potential COPD: Without COPD diagnosis but having a history of smoking and COPD-like exacerbations (subsequently referred to as exacerbations of potential COPD) indicating that a COPD diagnosis is likely.

High-risk patients are defined as patients who had had ≥2 moderate or ≥1 severe (i.e., requiring hospitalisation) exacerbations of COPD or potential COPD in the previous 12 months. The exacerbation algorithm included COPD exacerbation codes, COPD hospital admissions, or codes for COPD/LRTI/other lower respiratory/influenza within 3 days of prescribed OCS or respiratory-specific antibiotic, or hospital admission (Table 1). A sensitivity analysis was performed using a tighter definition of exacerbations that required a respiratory-related code within 3 days of a steroid or antibiotic prescription.

Patients were also required to have at least 12 months of adequate EMR data in the 12 months prior to and after the 1st of January of each study year. Patients with active asthma (i.e., clinical asthma consultation in the 12 months before the 1st of January of each year), other significant lung disease, or active cancer (except non-invasive skin cancer) were excluded.

### Objectives by quality standard

COPD management was assessed relative to Global Initiative for Chronic Lung Disease (GOLD) strategy document,^19^ current UK management guidelines^20^ and the CONQUEST QS,^13^ and included whether high-risk patients:

• Had no delay in diagnosis;
• Received an appropriate diagnosis, disease assessment and quantification of future risk of exacerbations and cardiac events;
• Received appropriate and prompt non-pharmacological and pharmacological interventions according to individual risk assessment and biological traits;
• Were regularly followed-up to review non-pharmacological and pharmacological interventions, utilising symptom and exacerbation assessment, evaluation of lifestyle risks, and prediction of future risk.

### Outcomes and opportunities

**Fig. 2:**
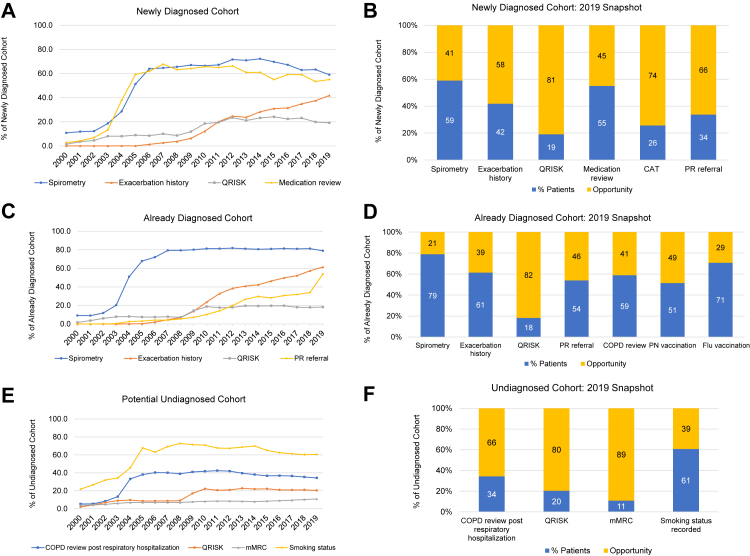
Proportion of high-risk patients with COPD who met relevant CONQUEST quality standards from 2009 to 2019; and opportunities for management improvements in 2019; **A and B**: newly diagnosed COPD patients; **C and D**: already diagnosed COPD patients; **E and F**: potential undiagnosed COPD patients. High-risk patients were those with ≥2 moderate or ≥1 severe (hospitalized) exacerbations in the last 12 months. See Table 2 for details and timings of all outcome variables. CAT: COPD Assessment Test; CONQUEST: The COllaboratioN on QUality improvement initiative for achieving Excellence in STandards of COPD care; COPD: chronic obstructive pulmonary disease; Flu: Influenza; mMRC: modified Medical Research Council; PN: pneumococcal; PR: pulmonary rehabilitation; QRISK: Cardiovascular risk (Pre-2007 we searched for any coding of cardiac risk, including Framingham score, Joint British Societies cardiac risk as well as additional evidence of cardiac risk assessments. The data were dominated by QRISK in post 2007).

The proportion of eligible patients classified as high-risk and time-to-classification were quantified. The latter was quantified by assessment of the time-to-classification once high-risk criteria were met. See S-Table 1 for outcome definitions and details, S-Table 2 for outcomes assessed for each patient cohort and S-Fig. 2A–C for timelines for each outcome assessed relative to date of follow-up or date of first COPD diagnosis.

### Data management and statistical analyses

Data were extracted from the OPCRD into a study dataset using UK Read and SNOMED code lists to identify relevant patient types, interventions, and assessments. The statistical analysis plan was pre-defined. RStudio version 1.4.171 (Boston, MA, USA) was used to conduct all statistical analyses. Demographic and clinical characteristics of the cohorts were described, based on data for five-year time periods (2000–2004, 2005–2009, 2010–2014, 2015–2019). We report COPD management in 2019 to show the most recent situation and opportunities for improvement and changes between 2000 and 2019.

### Role of funding source

This study is conducted by the Observational & Pragmatic Research International Ltd and was co-funded by Optimum Patient Care and 10.13039/100004325AstraZeneca. No funding was received by the Observational & Pragmatic Research Institute Pte Ltd (OPRI) for its contribution.

## Results

### Patient characteristics

The numbers of eligible patients and high-risk patients are presented in S-Table 3. In 2019, the latest year assessed, the mean (standard deviation) ages of the newly diagnosed, already diagnosed, and potential patients were 69.0 (11.6), 72.1 (10.4), and 62.5 (13.8) respectively, 49%, 51%, and 56%, respectively, were female, 95%, 96%, and 100%, respectively, were current or ex-smokers, 79%, 84%, and 76%, respectively, had had two or more moderate exacerbations, and 26%, 16%, and 17%, respectively, had had one or more severe exacerbations. Percent predicted FEV_1_ was 67.9% and 59.8% for the newly and already diagnosed cohorts respectively, prevalence of ICS mono prescription was low (6.3% and 1.3%, respectively) and triple therapy was prescribed in 10.7% and 53.1% of patients, respectively (Table 2). Compared to the diagnosed cohorts, undiagnosed patients were younger, more likely to be female, have a current or former smoking history, and have been prescribed no therapy, although moderate and severe exacerbation rates were similar to those of the newly diagnosed cohort (Table 2). Demographic characteristics for the earliest assessment period (2000–2004) were similar, although compared to 2019, newly and already diagnosed and undiagnosed patients all tended to experience more moderate and less severe exacerbations, had worse lung function, and were more likely to have been prescribed ICS mono. Triple therapy prescription was infrequent (Table 3). Detailed patient cohort characteristics are provided in S-Tables 4–7.

**Table 2:** High-risk patient demographic and baseline clinical characteristics (2019).

	Newly diagnosed	Already diagnosed	Undiagnosed	p value
**N**	1343	17,858	31,205	
**Age by index date, mean (SD)**	69.0 (11.6)	72.1 (10.4)	62.5 (13.8)	<0.001
**Female, n (%)**	660 (49.1)	9096 (50.9)	17,424 (55.8)	<0.001
**Ethnicity, n (%)**				
White	939 (69.9)	13,090 (73.3)	21,353 (68.4)	<0.001
Mixed/Multiple ethnic groups	3 (0.2)	42 (0.2)	154 (0.5)	
Asian/Asian British	31 (2.3)	231 (1.3)	1107 (3.6)	
Black/African/Caribbean/Black British	4 (0.3)	38 (0.2)	159 (0.5)	
Other ethnic group	49 (3.7)	801 (4.5)	1248 (4.0)	
Missing ethnicity	317 (23.6)	3656 (20.5)	7184 (23.0)	
**Smoking, n (%)**				
Never-smoker	62 (4.6)	703 (3.9)	0 (0.0)	<0.001
Current smoker	571 (42.5)	5934 (33.2)	12,044 (38.6)	
Former smoker	704 (52.4)	11,203 (62.7)	19,161 (61.4)	
Missing smoking status	6 (0.5)	18 (0.1)	0 (0.0)	
**BMI (within 5 years of index date), n (%)**				
Underweight (<18.5)	53 (4.0)	1001 (5.6)	586 (1.9)	<0.001
Normal weight (18.5–24)	398 (29.6)	5491 (30.8)	6974 (22.4)	
Overweight (25–29)	400 (29.8)	5473 (30.7)	9219 (29.5)	
Obese (30.0+)	435 (32.4)	5422 (30.4)	10,380 (33.3)	
Missing BMI	57 (4.2)	471 (2.6)	4046 (13.0)	
**BEC within 5 years of index date, mean (SD) count 10ˆ9/L**	0.2 (0.2)	0.2 (0.2)	0.2 (0.2)	<0.001
**Number of moderate exacerbations in baseline 12m, Mean (SD)**	2.7 (2.2)	3.6 (2.8)	2.7 (2.4)	<0.001
0, n (%)	150 (11.2)	1174 (6.6)	3454 (11.1)	<0.001
1, n (%)	138 (10.3)	1693 (9.5)	3901 (12.5)	
2, n (%)	522 (38.9)	5407 (30.3)	1403 (45.0)	
3+, n (%)	533 (39.7)	9584 (53.7)	9818 (31.5)	
**Number of severe exacerbations (hospital admittance for respiratory reason) in baseline 12m, mean (SD)**	0.3 (0.5)	0.2 (0.5)	0.2 (0.4)	<0.001
0	994 (74.0)	15,093 (84.5)	25,887 (83.0)	<0.001
1	320 (23.8)	2409 (13.5)	5007 (16.1)	
2+	29 (12.2)	356 (2.0)	311 (1.0)	
**Number of moderate exacerbations in follow-up 12m, Mean (SD)**	1.9 (2.6)	3.1 (3.2)	1.7 (2.7)	<0.001
**Number of severe exacerbations (hospital admittance for respiratory reason) in follow-up 12m, mean (SD)**	0.1 (0.4)	0.2 (0.5)	0.1 (0.3)	<0.001
**Number of rescue inhaler prescriptions in baseline 12m, mean (SD)**	2.7 (3.8)	6.4 (5.6)	0.6 (2.0)	<0.001
**Number of rescue inhaler prescriptions in follow-up 12m, mean (SD)**	4.2 (4.8)	6.1 (5.4)	0.6 (2.1)	<0.001
**MRC dyspnoea score recorded in 12 months before index date, mean (SD)**	2.3 (0.9)	2.8 (1.1)	2.1 (0.9)	<0.001
No MRC score, N (%)	729 (54.3)	4101 (23.0)	29,311 (93.9)	<0.001
**Spirometry values recorded in 12 months before index date, mean (SD)**				
FEV_1_% predicted	67.9 (18.7)	59.8 (20.4)	84.6 (20.1)	<0.001
**COPD therapy in baseline 12m, n (%)**				
No COPD therapy	282 (21.0)	1186 (6.6)	25,336 (81.2)	<0.001
Reliever only (SABA, SAMA and combinations)	341 (25.4)	1022 (5.7)	3118 (10.0)	
ICS only (mono)	85 (6.3)	236 (1.3)	1064 (3.4)	
LABA or LAMA only (mono)	205 (15.3)	1711 (9.6)	191 (0.6)	
LABA-ICS (dual)	156 (11.6)	2264 (12.7)	1124 (3.6)	
LABA-LAMA (dual)	91 (6.8)	1810 (10.1)	69 (0.2)	
LAMA-ICS (dual)	37 (2.8)	137 (0.8)	27 (0.1)	
LABA-LAMA-ICS (triple)	143 (10.7)	9478 (53.1)	191 (0.6)	
**Cambridge multimorbidity score, mean (SD)**	2.8 (1.4)	3.0 (1.4)	1.1 (1.3)	<0.001
**Hospital admission for any condition, mean (SD)**				
In baseline 12 m	0.8 (1.2)	0.8 (1.2)	0.7 (1.1)	<0.001
In follow-up 12 m	0.7 (1.2)	0.8 (1.3)	0.5 (1.1)	<0.001

**Table 3:** High-risk patient demographic and baseline clinical characteristics (2000–2004).

	Newly diagnosed	Already diagnosed	Undiagnosed	p value
**N**	3987	30,978	105,831	
**Age by index date, mean (SD)**	68.2 (10.6)	70.8 (10.4)	59.0 (12.5)	<0.001
**Female, n (%)**	1910 (47.9)	14,785 (47.7)	61,978 (58.6)	<0.001
**Ethnicity, n (%)**				
White	1557 (39.1)	11,111 (35.9)	55,561 (52.5)	<0.001
Mixed/Multiple ethnic groups	<5	8 (0.0)	204 (0.2)	
Asian/Asian British	24 (0.6)	171 (0.6)	1118 (1.1)	
Black/African/Caribbean/Black British	6 (0.2)	12 (0.04)	203 (0.2)	
Other ethnic group	312 (7.8)	2219 (7.2)	5487 (5.2)	
Missing ethnicity	2085 (52.3)	17,457 (56.4)	43,258 (40.9)	
**Smoking, n (%)**				
Never-smoker	260 (6.5)	2244 (7.2)	0 (0.0)	<0.001
Current smoker	1746 (43.8)	11,502 (37.1)	76,532 (72.3)	
Former smoker	1461 (36.6)	14,268 (46.1)	29,299 (27.7)	
Missing smoking status	520 (13.04)	2964 (9.6)	0 (0.0)	
**BMI (within 5 years of index date), n (%)**				
Underweight (<18.5)	191 (4.8)	1748 (5.6)	1977 (1.9)	<0.001
Normal weight (18.5–24)	1310 (32.9)	9887 (31.9)	27,516 (26.0)	
Overweight (25–29)	1019 (25.6)	7756 (25.04)	31,092 (29.4)	
Obese (30.0+)	706 (17.7)	5639 (18.2)	25,573 (24.2)	
Missing BMI	761 (19.1)	5948 (19.2)	19,673 (18.6)	
**BEC within 5 years of index date, mean (SD) count 10ˆ9/L**	0.2 (0.2)	0.2 (0.2)	0.2 (0.2)	<0.001
**Number of moderate exacerbations in baseline 12m, Mean (SD)**	3.2 (2.1)	3.7 (2.5)	2.7 (1.7)	<0.001
0	78 (2.0)	451 (1.5)	949 (0.9)	<0.001
1	270 (6.8)	1733 (5.6)	11,607 (11.0)	
2	1518 (38.1)	11,235 (36.3)	57,306 (54.2)	
3+	2121 (53.2)	17,559 (56.7)	35,969 (34.0)	
**Number of severe exacerbations (hospital admittance for respiratory reason) in baseline 12m, mean (SD)**	0.1 (0.3)	0.1 (0.3)	0.02 (0.1)	<0.001
0	3742 (93.9)	29,455 (95.1)	104,185 (98.4)	<0.001
1	231 (5.8)	1375 (4.4)	1613 (1.5)	
2+	14 (0.4)	148 (0.5)	33 (0.03)	
**Number of moderate exacerbations in follow-up 12m, mean (SD)**	2.4 (2.6)	3.1 (3.0)	1.5 (2.2)	<0.001
**Number of severe exacerbations (hospital admittance for respiratory reason) in follow-up 12m, mean (SD)**	0.03 (0.2)	0.1 (0.3)	0.01 (0.1)	<0.001
**Number of rescue inhaler prescriptions in baseline 12m, mean (SD)**	5.2 (7.6)	9.7 (10.0)	1.0 (3.3)	<0.001
**Number of rescue inhaler prescriptions in follow-up 12m, mean (SD)**	7.9 (9.01)	10.0 (10.2)	1.1 (3.6)	<0.001
**MRC dyspnoea score recorded in 12 months before index date, mean (SD)**	2.5 (0.9)	2.5 (0.9)	2.3 (0.9)	<0.001
No MRC score, N (%)	3401 (85.3)	27,503 (88.8)	102,740 (97.1)	<0.001
**Spirometry values recorded in 12 months before index date, mean (SD)**				
FEV_1_% predicted	56.5 (17.2)	50.1 (20.2)	80.3 (21.3)	<0.001
**COPD therapy in baseline 12m, n (%)**				
No COPD therapy	901 (22.6)	4245 (13.7)	83,635 (79.03)	<0.001
Reliever only (SABA, SAMA and combinations)	874 (21.9)	3973 (12.8)	8296 (7.8)	
ICS only (mono)	1455 (36.5)	11,826 (38.2)	10,027 (9.5)	
LABA or LAMA only (mono)	87 (3.2)	1142 (3.7)	304 (0.31)	
LABA-ICS (dual)	603 (15.1)	8422 (27.2)	3248 (3.1)	
LABA-LAMA (dual)	<5	51 (0.2)	0 (0.0)	
LAMA-ICS (dual)	8 (0.2)	163 (0.5)	11 (0.01)	
LABA-LAMA-ICS (triple)	13 (0.3)	529 (1.7)	17 (0.02)	
**Cambridge multimorbidity score, mean (SD)**	2.3 (1.1)	2.5 (1.2)	0.7 (0.9)	<0.001
**Hospital admission for any condition, mean (SD)**				
In baseline 12 m	0.2 (0.6)	0.2 (0.7)	0.1 (0.5)	<0.001
In follow-up 12 m	0.2 (0.6)	0.3 (0.8)	0.1 (0.5)	<0.001

### Proportion of eligible patients classified as high-risk COPD

**Fig. 3:**
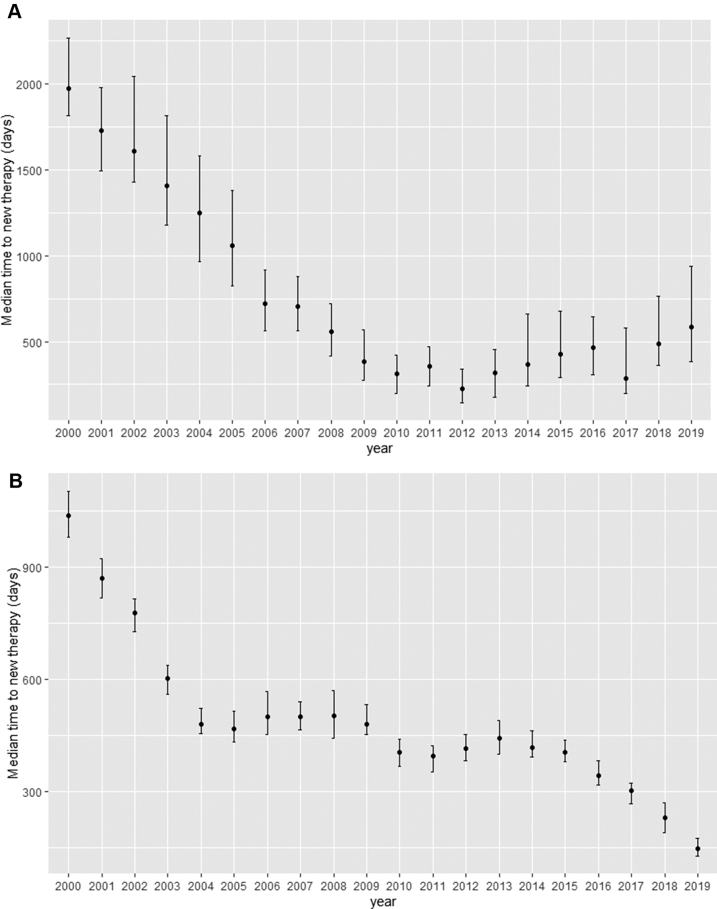
Median time to initiation of new therapy after an exacerbation for the **(A)** newly diagnosed and **(B)** already diagnosed cohorts.

In 2019, the proportions of high-risk patients among newly and already diagnosed COPD patients were 33% and 37%, respectively. These proportions had increased slightly between 2000 and 2019. As expected, the proportion of patients assessed as being high-risk in the undiagnosed cohort was much lower (6% in 2019) and had remained stable over time (S-Fig. 3; S-Table 3).

### Time between COPD diagnosis and meeting high-risk COPD criteria

Over the entire study period, the median time between COPD diagnosis (for already and newly diagnosed patients with COPD) and first meeting the high-risk criteria was 617 days (Q1-Q3: 3246) (i.e., 1.7 years), increasing to 729 days (Q1-Q3: 3509) (i.e., 2 years) during 2015–2019 (S-Fig. 4). However, over a third of patients (35%) were high-risk for many years *prior* to receiving a COPD diagnosis (Fig. 1).

### Analysis of high-risk patients according to CONQUEST QS in 2019 and over the period 2000–2019 by cohort

#### High-risk newly diagnosed patient cohort

In 2019, only 59% of high-risk newly diagnosed patients had a record of spirometry in the 12-month period prior to their COPD diagnosis. In the early 2000s, most newly diagnosed patients with COPD who met high-risk criteria had not had the diagnosis confirmed by spirometry; however, the situation changed rapidly after-2004, reaching a peak of 72% of patients with a record of spirometry in 2014 before declining again (Fig. 2A and S-Table 8). The prevalence of medication review within 6 months of treatment initiation or change in the 12-month period after diagnosis exhibited a similar longitudinal pattern; increasing rapidly post-2004 (59% in 2005), peaking in 2012 (66%), and gradually falling over recent years. Only 42% of patients were recorded as having had an exacerbation history review in the 12-month period before or after 1 January 2019 as part of a COPD annual review. In the early years of the study, no patients were recorded as having a review of exacerbation history; however, there was a steady increase between 2006 and 2019 (Fig. 2A and S-Table 8). In 2019, only 19% of patients without a prior history of cardiac disease had had a cardiac risk assessment (see S-Table 1 for details). The proportion having this assessment increased from 1% in 2000 to plateau at around 24% between 2014 and 2017, with a slight decrease in recent years (Fig. 2A and S-Table 8). We found virtually identical results in the sub-group of patients not on any cardiac medication (data not shown). Data on assessment of breathlessness and recording of smoking status are shown in S-Table 8.

In 2019, most newly diagnosed high-risk patients with COPD did not have an exacerbation history review (58%), cardiac risk assessment (81%), or COPD Assessment Test (CAT) assessment (75%) 12 months before or after diagnosis (Fig. 2B; S-Table 8). Furthermore, 66% of these patients had not been offered or received a referral for pulmonary rehabilitation (PR) if indicated (i.e. mMRC ≥2 before or after the 1st of January each year), 41% were diagnosed without a record of spirometry, 45% had not received a medication review within 6 months of treatment initiation or change, 15% did not have their smoking status recorded and, 17% did not have their modified Medical Research Council (mMRC) Dyspnoea Scale score recorded (Fig. 2B and S-Table 8). 21% of newly diagnosed high-risk patients did not receive any respiratory therapy in the year after diagnosis, and this had not changed since 2000 (S-Table 8). The median time to initiation or step-up to new maintenance therapy after an exacerbation in these patients was 584 days in 2019. This had fallen from 1975 days in 2000 to reach a minimum of 224 days in 2012 before increasing again (Fig. 3A).

#### High-risk already diagnosed patient cohort

In the 12-month period before or after 1 January 2019, 79% of high-risk patients with COPD in the already diagnosed cohort had had spirometry. Prior to 2004 spirometry had been performed infrequently (<20%) in these high-risk already diagnosed patients; however, its use increased dramatically in 2004 to 51% and continued to rise until plateauing at 81% in 2010 (Fig. 2C and S-Table 9). A diagnosis was not confirmed (i.e., FEV_1_/FVC >0.7) in about 10% of patients from 2004 onwards when spirometry was available (S-Fig. 5). There was no decline in its use in recent years. The prevalence of a recorded exacerbation history review in the 12 months before or after the 1st of January in the already diagnosed cohort increased steadily from 2% in 2006 to 61% in 2019 (Fig. 2C and S-Table 9). Only 18% of patients in the already diagnosed cohort without a prior history of cardiac disease had had a cardiac risk assessment in the 12-month period before or after 1 January 2019. The temporal pattern of cardiac risk assessment in this cohort was infrequent (<10%) from 2000 to 2008, increased in 2009 (14%) and 2010 (19%), and remained relatively stable until 2019) (Fig. 2C and S-Table 9). Just over half of eligible patients (54%) had been offered or referred for PR in the 12 months before or after 1 January 2019; however, this was a marked increase compared to the previous year (34%). Prior to 2010, offering/referral rates had been <10%, but subsequently there was a steady increase (Fig. 2C and S-Table 9). Data on assessment of breathlessness and smoking status are shown in S-Table 9.

In the 12-month period before or after 1 January 2019, most (82%) already diagnosed high-risk patients with COPD had had no assessment of cardiac risk, 39% had not had an exacerbation history review, and 41% had not received a COPD review within 6 weeks if hospitalisation for respiratory problem in the previous 12 months. 7% of the patients were not on any COPD medication. This percentage had decreased from 17% in 2000 to 8% in 2010 but had not decreased since then (S-Table 9). The median time to initiation or step-up to maintenance therapy after an exacerbation in these high-risk patients was 148 days in 2019. This had improved steadily since 2000 (Fig. 3B). 49% had not received a pneumococcal vaccination before 1 January 2019, and 29% had not received their influenza vaccination. 15% did not have their smoking status recorded, and 9% did not have their mMRC score recorded (Fig. 2D and S-Table 9).

#### High- risk potential undiagnosed patient cohort

In 2019, 4% patients in this cohort had had spirometry, and 20% without a prior history of cardiac disease had had a cardiac risk assessment in the 12-month period before or after 1 January. The proportion of patients with a cardiac risk assessment had remained stable since 2010, having progressively increased between 2000 and 2010. The prevalence of an mMRC assessment in this cohort in 2019 (12-months before or after 1 January) was low (11%); however, this had increased gradually from 4% in 2000 (Fig. 2E and S-Table 10). In 2019, there was a significant opportunity to improve the management of these undiagnosed potential COPD patients at high risk of exacerbating. In particular, in the previous 12 months, 66% had not received a review including COPD assessment tools following a respiratory hospitalisation, and 40% had no record of smoking status. 80% had not had a cardiac risk assessment 12 months before or after 1 January 2019 (Fig. 2F and S-Table 10). Approximately 2% of undiagnosed patients received a diagnosis of COPD within the subsequent year; however, most of these patients still had not had spirometry (S-Table 10). Nearly 20% of undiagnosed patients were receiving COPD treatments without a diagnosis (S-Table 10).

#### Sensitivity analysis

Similar findings were seen when a definition of high-risk based on a tighter definition of exacerbations requiring a respiratory-related code within 3 days of a steroid or antibiotic prescription (S-Fig. 6A and 6B, S-Tables 11–18).

## Discussion

Although COPD was identified as a priority for health services in the UK over 10 years ago, this analysis of routinely available primary care practice data shows that there are still many opportunities to improve management. Our findings rely on the accuracy of recording of reviews in patients’ EMRs, but as incentive payments through the quality and outcomes framework (QOF)^21^ are linked to records of many of these activities (i.e., spirometry, review of exacerbation history, MRC score, and PR referral) we are confident that if assessments and reviews had been performed, they would have been recorded in the EMR and that the opportunities identified are real.

We noted a trend for increasing number of diagnosed patients with COPD over time, most likely due to fact that practices contributing data to OPCRD have grown in size over time, hence the population denominator has increased. Other factors that may have had a small effect on number of patients with COPD include the ageing population, improved clinical recording and assessment in response to the introduction of QOF, and by nature of the study design. As an annual cross-sectional study, each year a segment of the undiagnosed potential COPD cohort moved into the newly diagnosed cohort in the next year if they had a recorded COPD diagnosis, and a segment of the newly diagnosed cohort moved into the already diagnosed cohort in the years following. Patients were only included in the study years in which they met the eligibility criteria and had experienced ≥2 exacerbations in the previous 12 months.

Accurate diagnosis is crucial for effective management, yet in the most recent year of our analysis (2019), over 40% of newly diagnosed patients had not had spirometry recorded in the previous year. These findings are similar to rates of use of spirometry to confirm diagnosis reported in other countries such as Denmark, Sweden, and the United States,^22^, ^23^, ^24^ but higher than in South Korea.^25^ There was also evidence that rather than improving over time, there has been a decline in the proportion of patients who have had spirometry at diagnosis in recent years, despite guidance from the national health outcomes strategy, NICE, and international guidelines/strategy documents. There is, therefore, a clear opportunity to improve COPD management by increasing the use of spirometry to confirm the diagnosis. It is also important to identify the reasons for the decline in the use of spirometry and develop ways of addressing them. Approximately three-quarters of patients with an established diagnosis of COPD had spirometry in the 12-month period before or after 1 January 2019, and this proportion had remained constant over the last 10 years. The use of spirometry in both cohorts showed a marked increase between 2003 and 2006. This coincided with the publication of the NICE guidelines on COPD^26^ and the introduction of payments through QOF.

Our study population includes patients who were identified from their EMR as potentially having COPD based on their smoking history and occurrence of exacerbation-like events. As they remained undiagnosed, it is to be expected that the frequency of assessment with spirometry was low, yet there was a missed opportunity to confirm or refute a diagnosis of COPD. A small number of these patients were diagnosed with COPD in the subsequent year; however, most of these patients had also not had spirometry. Screening and case finding for COPD in the general population are not recommended because the yield is thought to be low, thus making it not cost-effective.^27^^,^^28^ However, identification of potential patients with COPD using the CONQUEST methodology offers a simple—and potentially cost effective—method of finding patients suitable for diagnostic assessment, 6% of whom qualified as high-risk in 2019. The fact that 1 in 5 of these patients were receiving respiratory medications suggests that their clinicians were treating their symptoms but had not yet made or formally recorded a diagnosis.

Recording the frequency of exacerbations and assessments of breathlessness are indicators in QOF, as these factors are required to guide therapy and to create personalized management plans. There are still clear opportunities to improve the assessment of high-risk patients by increasing the proportion of patients having these reviews of exacerbation history from the current levels of 42% and 61% in and newly and already diagnosed patients, respectively. Recording of breathlessness was good in established patients, but there were opportunities to improve assessment of symptoms in newly diagnosed patients, with 17% not having a record in their EMR. Although the recording of smoking status—the main modifiable risk for COPD—was good, there was still room for improvement, particularly for potential undiagnosed patients.

Assessment and management of comorbidities is a key component of COPD management and is included in both national and international recommendations.^19^^,^^20^^,^^29^ Although cardiovascular disease is one of the commonest causes of death in patients with COPD,^3^ only 1 in 5 patients without a history of cardiac disease had an assessment of cardiac risk. Addressing this gap in current care offers a major opportunity to improve management and outcomes.

20% of newly diagnosed patients were not receiving any inhaled therapy in the year after diagnosis, and 6% of the already diagnosed patients were on no respiratory therapy despite meeting the criteria of being high-risk. Patients also waited a significant time after an exacerbation for initiation or step-up to new medication. Medication reviews are essential to assess whether therapeutic goals have been achieved, whether the medication is being taken as recommended, and whether there are adverse effects. However, just over half of newly diagnosed patients had a review of their medication within 6 months of initiating or changing therapy. There is a significant opportunity to improve management by ensuring all patients have a review following initiation of, or changes to, therapy. This is particularly important for newly diagnosed patients who may not have used an inhaler before or may not know what to expect from their medication.

Similarly, review following hospitalisation for an exacerbation provides an opportunity to ensure that the patient is receiving appropriate pharmacological and non-pharmacological therapy, is using their inhaler correctly and is adherent, and is addressing potential triggers and their future avoidance. National Asthma and COPD Audit Programme (NACAP) data show readmission rates at 30 days have increased from 33% to 43% between 2008 and 2019.^30^ 41% of already diagnosed patients did not have a review within 6 weeks of hospitalisation; this is another important missed opportunity to improve outcomes.

Pulmonary rehabilitation is one of the most effective interventions in COPD. It leads to clinically significant improvements in symptoms, exercise capacity, and health-related quality of life, and results in fewer and shorter hospital stays and readmission,^31^ and is recommended for all patients with exercise limitation due to breathlessness.^19^^,^^25^ Referral rates are included in the NICE QS^20^ and feature in QOF; however, only approximately one-third of newly diagnosed patients and half of already diagnosed patients had been offered or received a PR referral. To some extent this may reflect the poor availability and capacity of PR programmes^32^ but improving access to PR and referring appropriate patients offers another important opportunity to improve patients’ wellbeing and reduce hospitalisation rates.

Given the pressures on primary care, it seems likely that more opportunities to improve the management of high-risk patients with COPD have been missed and, meaning there is now an even greater need to address these issues.

### Strengths

The OPCRD is a high-quality data source that is well described, representative of the UK COPD population, and has been extensively used in respiratory research.^16^^,^^18^ The study includes a large number of patients and reports data from across the UK, highlighting differences between recommendations and QS for COPD and real-world management in the UK. The 20 years of data give a unique insight into trends in management and the influence of guidelines/strategies and implementation initiatives.

### Limitations

Although the OPCRD is a well-maintained and validated database, we cannot rule out the possibility of inaccurate or missing data and, in earlier years of the study period, assessments may have been carried out but were either not recorded or were recorded in other health records (e.g., secondary care). The data solely reflect UK practice and cannot be used to identify opportunities to improve COPD care in other countries that are part of the CONQUEST initiative. The findings are based on information contained in the primary care records and it is possible that some management opportunities related to the QS may have been addressed by care provided by community respiratory services and hospitals. Definition of treatment optimization was based on initiation/step up to new pharmacological treatment but not on the specifics of initiated or stepped-up treatment. Future research is planned to examine this issue in more detail in our cohort but is beyond the scope of the current study. It has been examined in detail elsewhere.^6^ The study does not take into account the effects of the COVID-19 pandemic on the management of COPD in primary care. Some routine reviews have continued remotely, but data from the DHSC show that the rate of new diagnoses has fallen to about a half of the rate pre-pandemic.^33^

### Conclusions

Our analysis shows that opportunities for timely diagnosis of patients with COPD at high risk of exacerbating are being missed. Newly and already diagnosed high-risk patients are not being assessed or treated promptly, and there is substantial scope to improve assessment of these patients and optimize their treatment. Ways of achieving this are summarized in Table 4.

**Table 4:** Summary of recommendations to improve management of high-risk patients with COPD.

• Ensure all patients diagnosed with COPD have had spirometry that confirms the diagnosis at least once • Perform spirometry to confirm or refute a diagnosis of COPD in all patients presenting with symptoms suggestive of COPD or recurrent chest infections. • Ensure the frequency of both exacerbations and assessments of breathlessness is recorded for all patients with COPD and, if necessary, an appropriate change in management is made. • Ensure co-morbidities are actively sought, diagnosed and managed. • Ensure patients are reviewed regularly to check pharmacological and non-pharmacological therapy follows guideline/strategy document recommendations for individual patients taking into account the severity of symptoms and risk of exacerbations. • Ensure patients are reviewed following an exacerbation to ensure that they are receiving appropriate therapy to reduce the risk of future events.

## Contributors

The authors meet criteria for authorship as recommended by the International Committee of Medical Journal Editors. All authors had full access to the data in this study and take complete responsibility for the integrity of the data and accuracy of the data analysis, and responsibility for the integrity of the work as a whole. DMGH & RM wrote a first draft of the paper. All authors discussed and interpreted the results, contributed to the data analyses, contributed to the writing and reviewing of the manuscript, and have given final approval for the version to be published.

## Data sharing statement

The dataset supporting the conclusions of this article was derived from the Optimum Patient Care Research Database (www.opcrd.co.uk). The OPCRD has ethical approval from the National Health Service (NHS) Research Authority to hold and process anonymised research data (Research Ethics Committee reference: 15/EM/0150). This study was approved by the Anonymised Data Ethics Protocols and Transparency (ADEPT) committee – the independent scientific advisory committee for the OPCRD. The authors do not have permission to give public access to the study dataset; researchers may request access to OPCRD data for their own purposes. Access to OPCRD can be made via the OCPRD website (https://opcrd.co.uk/our-database/data-requests/) or via the enquiries email info@opcrd.co.uk.

## Declaration of interests

**David MG Halpin** has received sponsorship to attend international meetings, and honoraria for lecturing, attending advisory boards and preparing educational materials from AstraZeneca, Boehringer Ingelheim, Chiesi, GSK, Novartis and Pfizer.

**Andrew P. Dickens, Rachel Pullen, and Amy Couper**, are employees of the Observational and Pragmatic Research Institute, which is a research collaborator of the CONQUEST initiative with Optimum Patient Care and AstraZeneca.

**Alexander Evans, Victoria Carter, Derek Skinner and Ruth Murray** are employees of Optimum Patient Care, UK, which is a research collaborator of the CONQUEST initiative with Optimum Patient Care and AstraZeneca.

**Mukesh Singh** reports personal fees from AstraZeneca, Boehringer Ingelheim, Cheisi, GlaxoSmithKline, Napp/Mundipharma, Pfizer, Teva.

**Tamsin Morris, Shruti Menon and Hana Muellerova** are employees of AstraZeneca and hold stock and/or stock options in the company. AstraZeneca is a co-funder of the CONQUEST initiative.

**Mona Bafadhel** reports grants from AstraZeneca and personal fees and non-financial support from AstraZeneca, Chiesi, GSK and others from AlbusHealth, outside the submitted work.

**James Chalmers** has received research grants or consultancy fees from AstraZeneca, Boehringer Ingelheim, Gilead Sciences, GlaxoSmithKline, Grifols, Insmed and Zambon.

**Katherine Hickman, Graham Devereux, Martin Gibson, Jennifer Quint,** and **Marije van Melle** report no conflicts of interest.

**John Hurst** has received personal payment and payment to his institution (UCL), including research grants, reimbursement for advisory work and educational activities, and support to attend meetings from pharmaceutical companies that make medicines to treat COPD, which includes AstraZeneca, Boehringer Ingelheim, Chiesi, and Novartis.

**Rupert C. Jones** declares grants from AstraZeneca, GlaxoSmithKline, Novartis, and Teva, and personal fees for consultancy, speakers’ fees or travel support from AstraZeneca, Boehringer Ingelheim, GlaxoSmithKline, Novartis, and Observational & Pragmatic Research Institute Pte Ltd (OPRI).

**Konstantinos Kostikas** has received honoraria for presentations and consultancy fees from AstraZeneca, Boehringer Ingelheim, CSL Behring, Chiesi, ELPEN, GILEAD, GSK, Menarini, Novartis, Sanofi, Specialty Therapeutics; WebMD (paid to the University of Ioannina); his department has received funding and grants from AstraZeneca, Boehringer Ingelheim, Chiesi, Innovis, ELPEN, GSK, Menarini, Novartis and NuvoAir (paid to the University of Ioannina); KK is a member of the GOLD Assembly.

**Dave Singh** has received personal fees from GSK, Cipla, Genentech and Peptinnovate, and personal fees and grant support from AstraZeneca, Boehringer Ingelheim, Chiesi, Glenmark, Gossamerbio, Menarini, Mundipharma, Novartis, Pfizer, Pulmatrix, Theravance, and Verona.

**Tom Wilkinson** is the co-founder, shareholder and director of Mymhealth Limited; he has received grants or consultancy fees from GSK, AstraZeneca, Janssen, Bergenbio, UCB, Olam, Valneva, Synairgen, Novavax, Teva, BI.

**David Price** has advisory board membership with AstraZeneca, Boehringer Ingelheim, Chiesi, Mylan, Novartis, Regeneron Pharmaceuticals, Sanofi Genzyme, Thermofisher; consultancy agreements with Airway Vista Secretariat, AstraZeneca, Boehringer Ingelheim, Chiesi, EPG Communication Holdings Ltd, FIECON Ltd, Fieldwork International, GlaxoSmithKline, Mylan, Mundipharma, Novartis, OM Pharma SA, PeerVoice, Phadia AB, Spirosure Inc, Strategic North Limited, Synapse Research Management Partners S.L., Talos Health Solutions, Theravance and WebMD Global LLC; grants and unrestricted funding for investigator-initiated studies (conducted through Observational and Pragmatic Research Institute Pte Ltd) from AstraZeneca, Boehringer Ingelheim, Chiesi, Mylan, Novartis, Regeneron Pharmaceuticals, Respiratory Effectiveness Group, Sanofi Genzyme, Theravance and UK National Health Service; payment for lectures/speaking engagements from AstraZeneca, Boehringer Ingelheim, Chiesi, Cipla, GlaxoSmithKline, Kyorin, Mylan, Mundipharma, Novartis, Regeneron Pharmaceuticals and Sanofi Genzyme; payment for travel/accommodation/meeting expenses from AstraZeneca, Boehringer Ingelheim, Mundipharma, Mylan, Novartis, Thermofisher; stock/stock options from AKL Research and Development Ltd which produces phytopharmaceuticals; owns 74% of the social enterprise Optimum Patient Care Ltd (Australia and UK) and 92.61% of Observational and Pragmatic Research Institute Pte Ltd (Singapore); 5% shareholding in Timestamp which develops adherence monitoring technology; is peer reviewer for grant committees of the UK Efficacy and Mechanism Evaluation programme, and Health Technology Assessment; and was an expert witness for GlaxoSmithKline.

## Appendix A Supplementary data

Supplementary S-Fig. 1

Supplementary S-Fig. 2A

Supplementary S-Fig. 2B

Supplementary S-Fig. 2C

Supplementary S-Fig. 5

Supplementary S-Fig. 6A

Supplementary S-Fig. 6B

Supplementary material

Caption for supplementary material
