# Supplementary figures and images for: Identification of key opportunities for optimising the management of high-risk COPD patients in the UK using the CONQUEST quality standards: an observational longitudinal study
Source: Lancet Reg Health Eur. 2023 Apr 21;29:100619. doi: 10.1016/j.lanepe.2023.100619 (PMC10149261; doi:10.1016/j.lanepe.2023.100619)

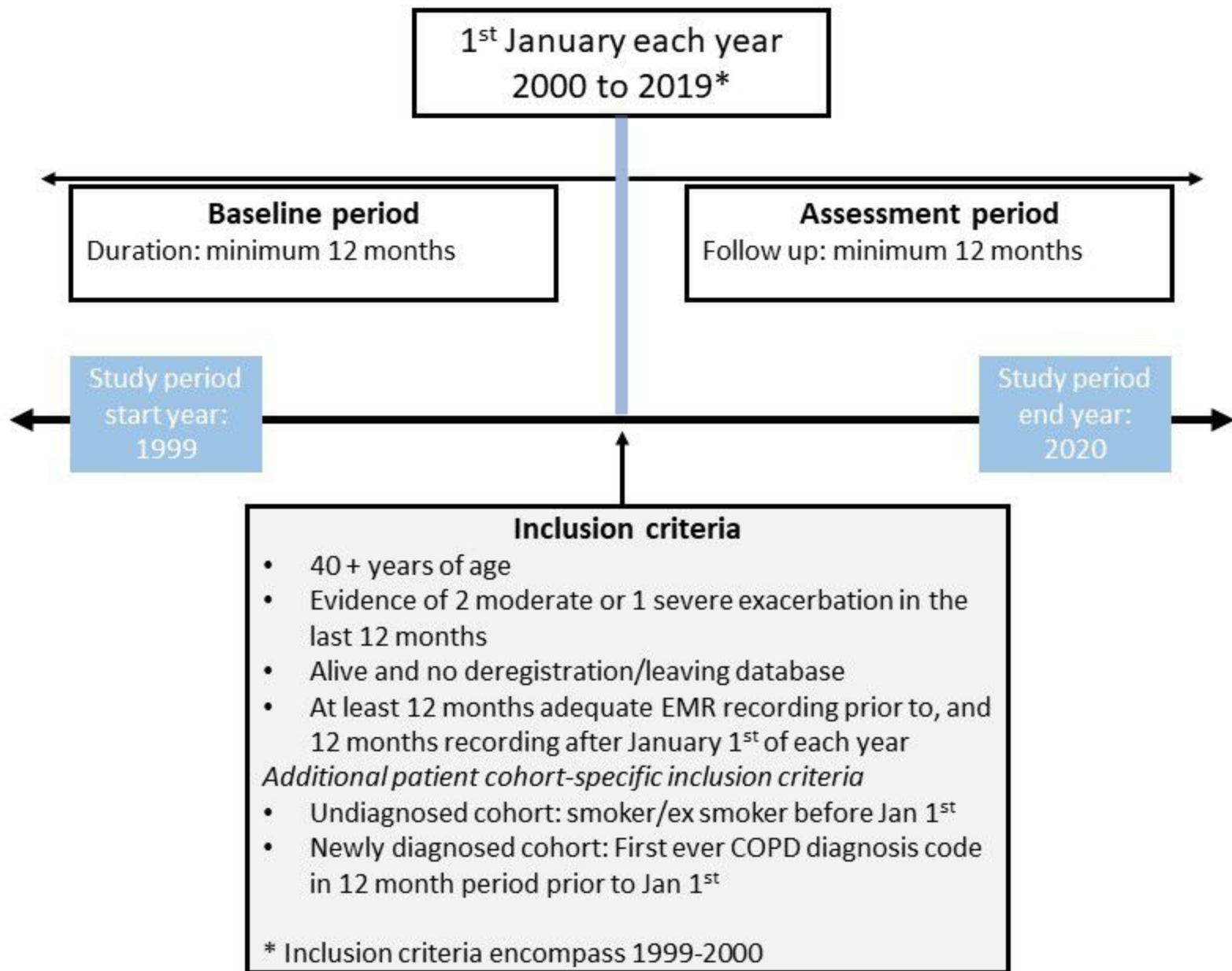

Supplement: Supplementary S-Fig. 1 [file mmc1.pdf]

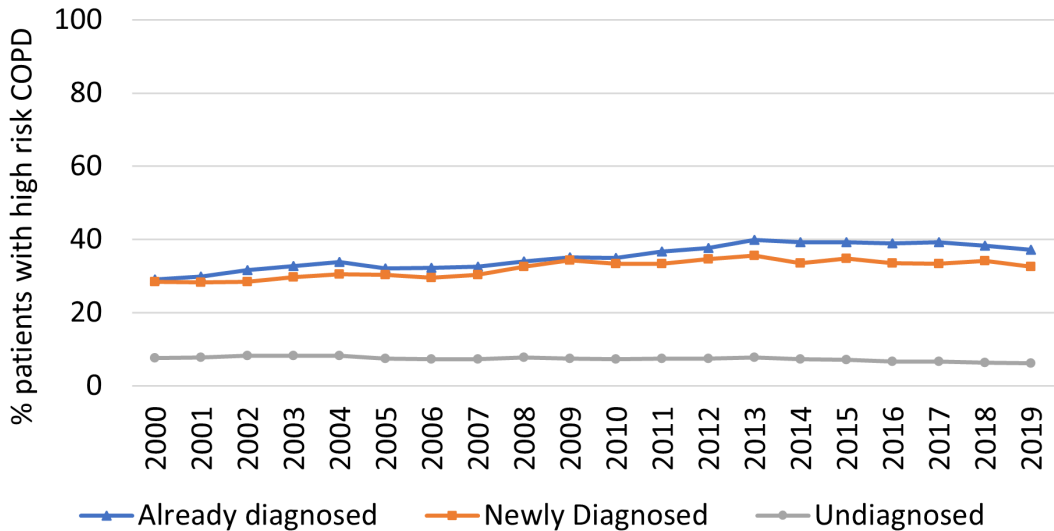

Supplement: Supplementary S-Fig. 3 [file mmc5.pdf]

First High Risk to COPD diagnosis

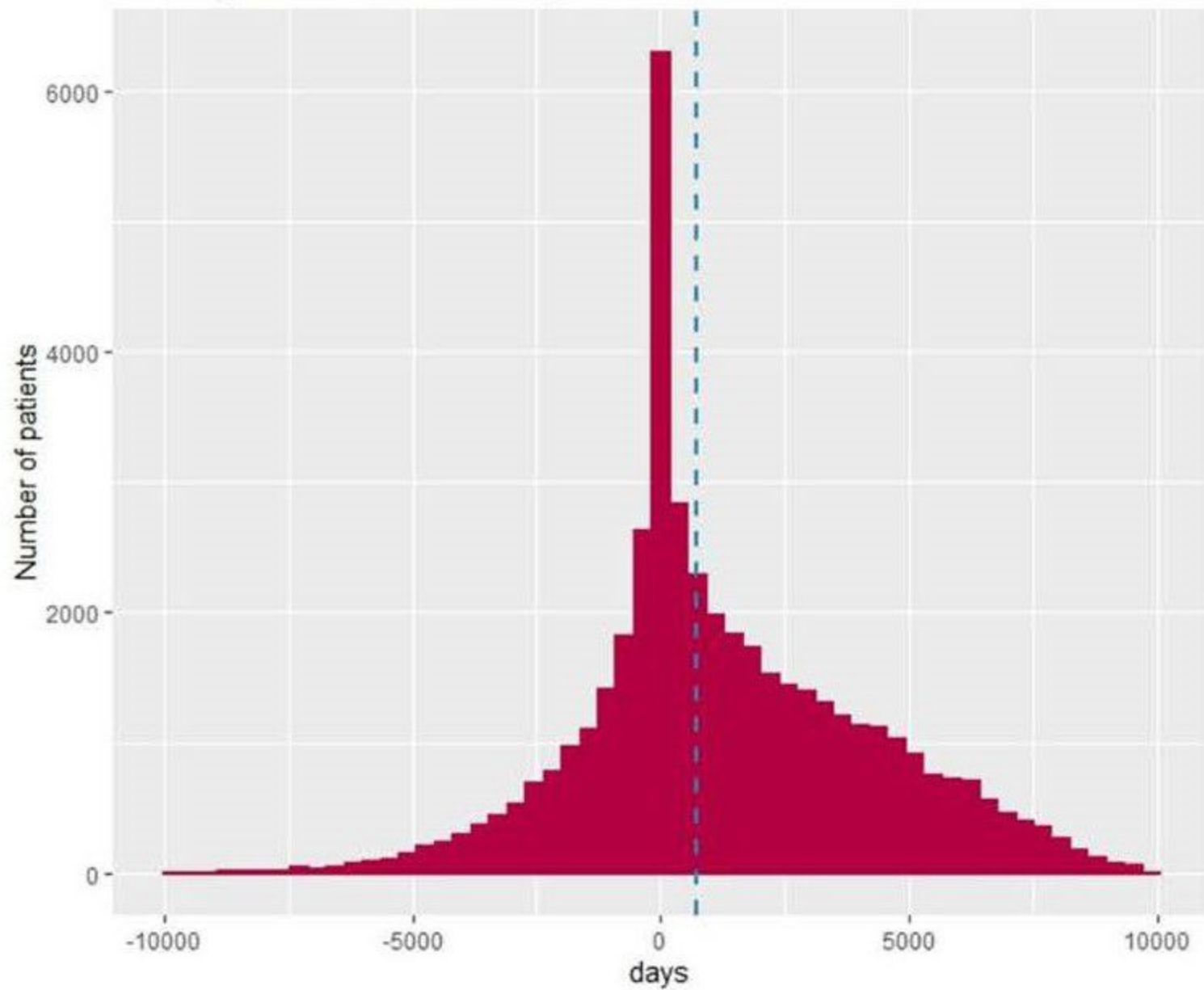

Supplement: Supplementary S-Fig. 4 [file mmc6.pdf]

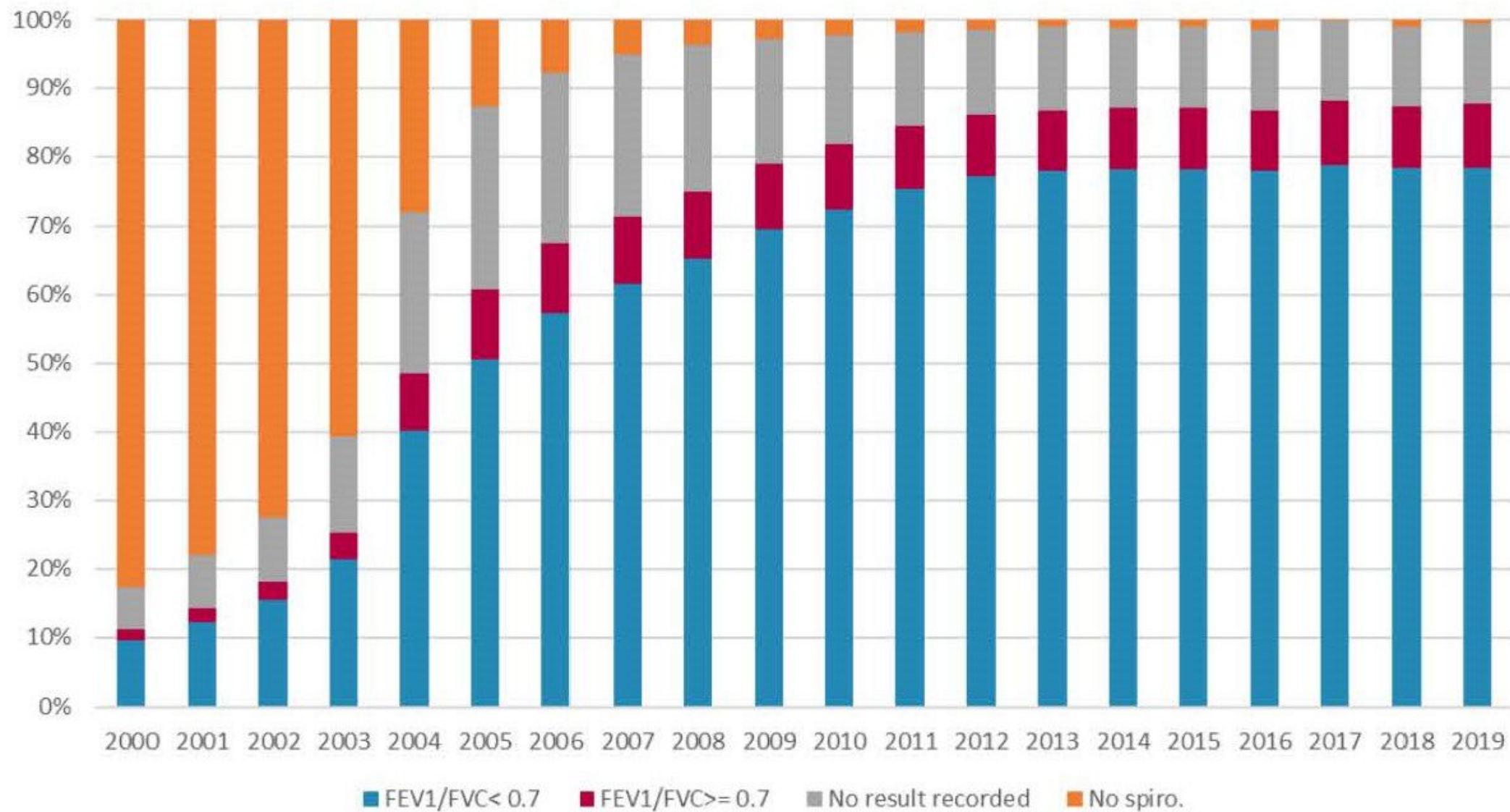

Supplement: Supplementary S-Fig. 5 [file mmc7.pdf]

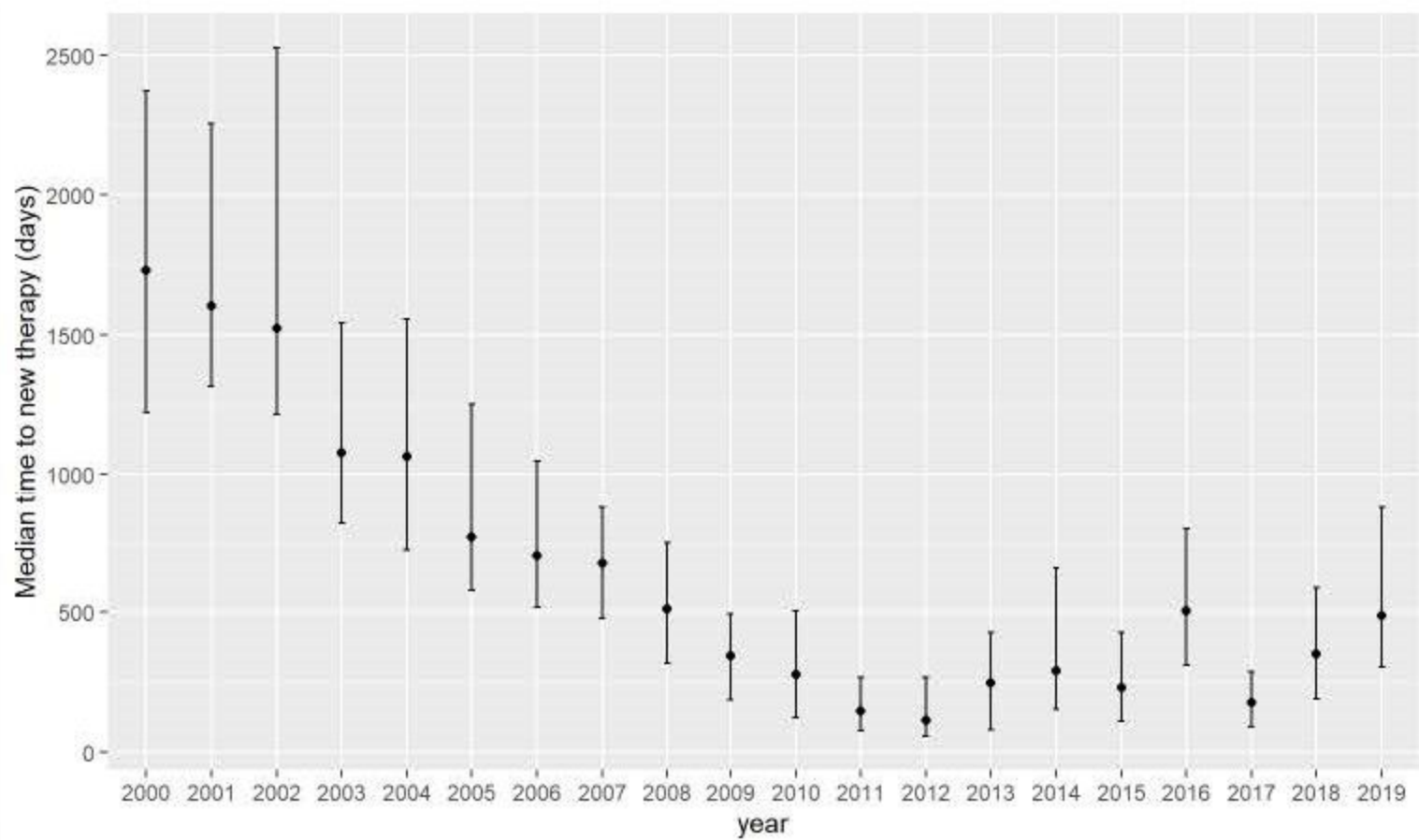

Supplement: Supplementary S-Fig. 6A [file mmc8.pdf]

Median time to new therapy (days)

year

2000 2001 2002 2003 2004 2005 2006 2007 2008 2009 2010 2011 2012 2013 2014 2015 2016 2017 2018 2019

900

600

300

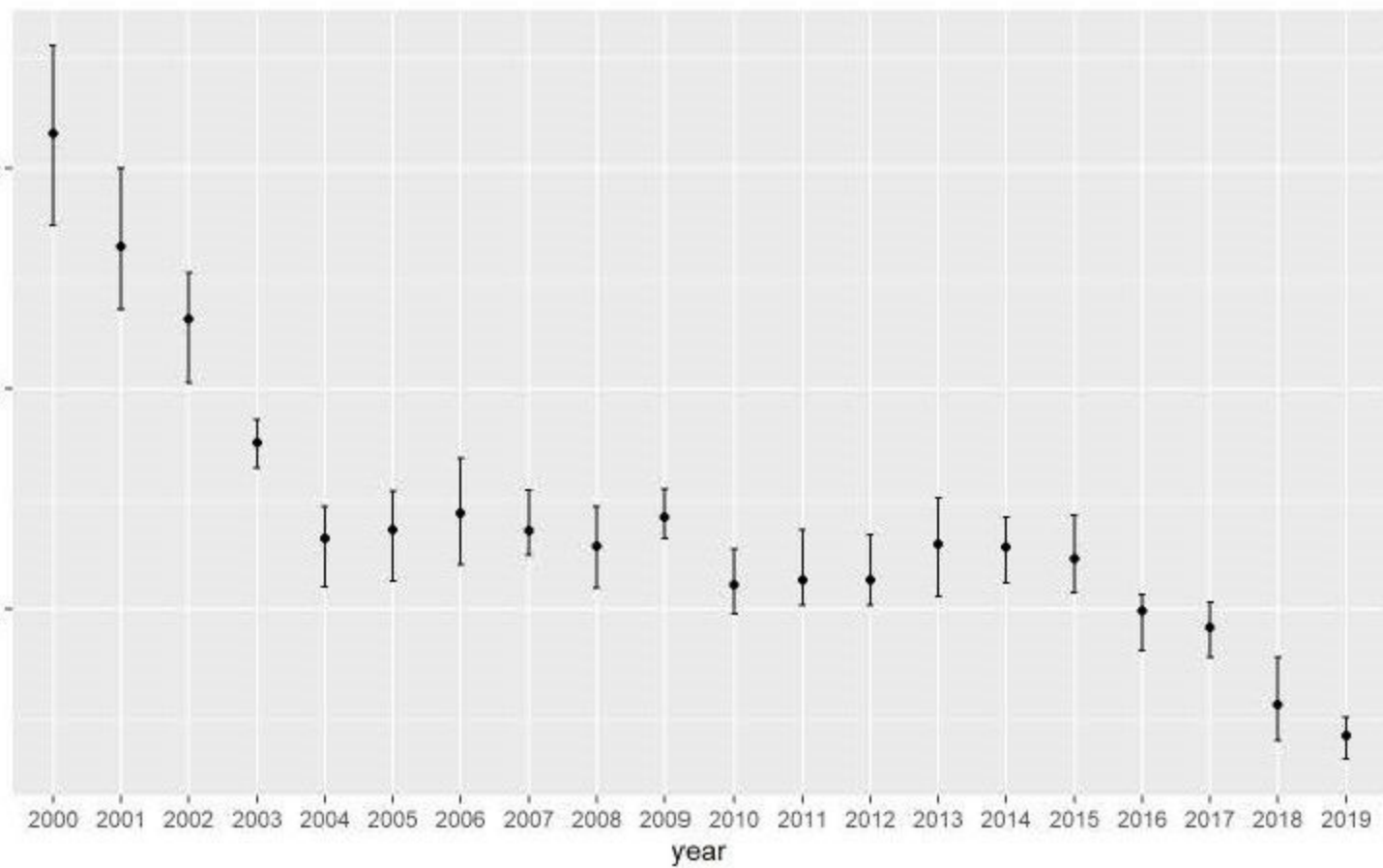

Supplement: Supplementary S-Fig. 6B [file mmc9.pdf]
