# Supplementary material for: Identification of key opportunities for optimising the management of high-risk COPD patients in the UK using the CONQUEST quality standards: an observational longitudinal study
Source: Lancet Reg Health Eur. 2023 Apr 21;29:100619. doi: 10.1016/j.lanepe.2023.100619 (PMC10149261; doi:10.1016/j.lanepe.2023.100619)
Supplement: Supplementary S-Fig. 2A [file mmc2.pdf]

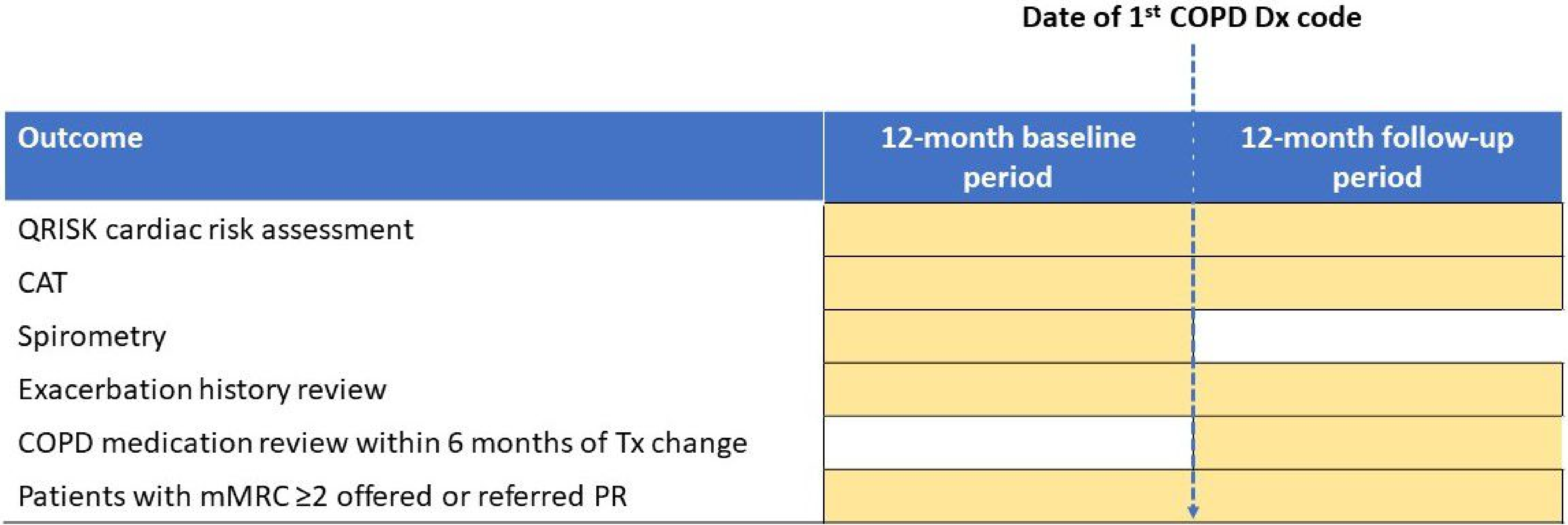
Date of 1st COPD Dx code
Outcome
12-month baseline period
12-month follow-up period
QRISK cardiac risk assessment
CAT
Spirometry
Exacerbation history review
COPD medication review within 6 months of Tx change
Patients with mMRC ≥2 offered or referred PR
