# Supplementary material for: Identification of key opportunities for optimising the management of high-risk COPD patients in the UK using the CONQUEST quality standards: an observational longitudinal study
Source: Lancet Reg Health Eur. 2023 Apr 21;29:100619. doi: 10.1016/j.lanepe.2023.100619 (PMC10149261; doi:10.1016/j.lanepe.2023.100619)
Supplement: Supplementary S-Fig. 2C [file mmc4.pdf]

1 January of each year

Outcome	12 month baseline period	12 month follow-up period
Smoking status		
COPD review within 6 weeks of respiratory hospitalization		
QRISK Cardiac Risk Assessment		
mMRC		
