# Supplementary material for: Identification of key opportunities for optimising the management of high-risk COPD patients in the UK using the CONQUEST quality standards: an observational longitudinal study
Source: Lancet Reg Health Eur. 2023 Apr 21;29:100619. doi: 10.1016/j.lanepe.2023.100619 (PMC10149261; doi:10.1016/j.lanepe.2023.100619)
Supplement: Supplementary material [file mmc10.docx]

**Methods**

Data capture

Free text is not extracted into OPCRD. To cover this insecurity/incompleteness, we identified all indications of a COPD review including the generic COPD review codes (often used for QOF purposes), but also all components that can be part of such a review. That leads to a list of not entirely equivalent terms/indicators that all contribute to a COPD review according to national COPD guidelines, ensuring our lists are as complete as possible. For each of these indicators, we searched all relevant codes, such as Read codes v2 and v3 and SNOMED codes, including but not limited to QOF-related codes.

**S-Table 1: Outcome definitions**

| **Term** | **Definition** |
| --- | --- |
| **Outcomes** | |
| **Cambridge Multimorbidity Score** | The score was calculated by summing the standardised weights of 20 individual long-term conditions, to represent overall multimorbidity burden.^1^ |
| **CAT** | Based on valid entries in COPD Assessment Test (CAT). The time frame for this analysis differed depending on the patient group: i) newly diagnosed patients: 12 months either side of the first COPD diagnostic code, ii) already diagnosed/ undiagnosed patients: 12 months before or after 1 January in each year. |
| **COPD medication review** | The proportion of patients with a medical review within 6 months of change of pharmacological therapy. Analysis of medication review data was based on valid entries in any of the following indicators: medication review, and COPD self-management advice. The denominator was patients initiating mono, dual, or triple therapy for COPD. |
| **COPD review** | COPD review within 6 weeks of respiratory hospitalization. Analysis of COPD review data was based on valid entries in any of the following indicators: medication review, annual review, spirometry, MRC, CAT, total exacerbations in last 12 months, COPD self-management advice, inhaler technique, and pulmonary rehabilitation (offered/referred/accepted). |
| **Exacerbation review** | Analysis of COPD exacerbation data was based on valid entries in the following indicator: total exacerbations in the last 12 months. It was not possible to analyse data for validated COPD risk assessments such as Dyspnoea, Obstruction, Smoking, Exacerbation (DOSE) index or body-mass index, airflow obstruction, dyspnoea, and exercise capacity index (BODE), due to insufficient data availability within OPCRD. The time frame for these analyses differed depending on the patient group: i) newly diagnosed patients: 12 months before or after the first COPD diagnostic code, ii) already diagnosed patients: 12 months either side of the index date, i.e., 1 January in each year |
| **Flu vaccination** | Analysis of influenza vaccination data was based on valid entries in this indicator. The numerator for this analysis was patients receiving an influenza vaccination 12 months before or after 1 January, as guidelines recommend annual vaccinations for adults aged 50 years or above and those with long-term health conditions, including COPD. <https://www.nhs.uk/conditions/vaccinations/flu-influenza-vaccine/> |
| **mMRC** | Based on valid entries in mMRC dyspnoea scale. The time frame for this analysis differed depending on the patient group: i) newly diagnosed patients: 12 months before or after the first COPD diagnostic code, ii) already diagnosed/ undiagnosed patients: 12 months before or after index date, i.e., 1 January in each year. |
| **Optimised maintenance therapy** | Initiation or change in pharmacological treatment in response to recent exacerbations. |
| **Cardiac risk*** | Patients who received cardiac risk assessment based on EMR codes either being accepted or declined. The time frame for these analyses differed depending on the patient group: i) newly diagnosed patients: 12 months before or after the first COPD diagnostic code, ii) already diagnosed/ undiagnosed patients: 12 months before or after the index date, i.e., 1 January in each year. |
| **Smoking status** | Based on valid entries in this indicator. Recorded in the 12-month period before 1 January of each study year |
| **Spirometry** | Analysis of spirometry data was based on valid entries in any of the following indicators: FEV_1_, FVC, FEV_1_/FVC. Assessed in the 12-month period before 1 January of each study year (newly diagnosed) or in the 12-month period before or after 1 January of each study year (already diagnosed). |
| **PN vaccination** | Analysis of pneumococcal vaccination data was based on valid entries in this indicator. The numerator for this analysis was patients who had ever received a pneumococcal vaccination, as guidelines (<https://www.nhs.uk/conditions/vaccinations/when-is-pneumococcal-vaccine-needed/>) recommend a one-off vaccination for adults aged 65 years or above and those at increased risk of infection, including patients with COPD. |
| **PR review** | Based on valid entries in any of the following indicators: offered pulmonary rehabilitation, referred for pulmonary rehabilitation and assessed the proportion of patients with dyspnoea score of MRC 3+ or mMRC 2+ within 12 months before or after 1 January of each study year who have been offered or referred for pulmonary rehabilitation within 12 months of MRC score. |
| BMI: Body Mass Index; BODE: body-mass index, airflow obstruction, dyspnoea, and exercise capacity index; CAT: COPD Assessment Test; COPD: chronic obstructive pulmonary disease; DOSE: Dyspnoea, Obstruction, Smoking, Exacerbation; EMR: electronic medical record; FEV_1_: forced expiratory volume in one second; FVC: forced vital capacity; MRC: Medical Research Council Dyspnoea Scale; mMRC: Modified Medical Research Council Dyspnoea Scale; OPCRD: Optimum Patient Care Research Database; PN: pneumococcal; PR: pulmonary rehabilitation; QRISK: Cardiovascular Risk Score  * Pre-2007 we searched for any coding of cardiac risk, including Framingham score, Joint British Societies cardiac risk as well as additional evidence of cardiac risk assessments. The data were dominated by QRISK post 2007. QRISK is an algorithm which calculates an individual's 10-year risk of having a heart attack or stroke. Information used to assess this risk include both demographic (age, gender, ethnicity) and clinical variables (e.g., smoking and diabetes status, presence of certain co-morbid conditions (e.g., chronic kidney disease, atrial fibrillation, rheumatoid arthritis, systemic lupus erythematous, mental illness and erectile dysfunction), current treatment(s) (e.g., blood pressure, antipsychotic and/or regular oral steroid treatment), cholesterol/HDL ratio, blood pressure, and body mass index). | |

**S-Table 2: Time course of outcomes assessed for each patient cohort in this article**

| **Outcomes assessed each study year (2000 – 2019)** | **Newly diagnosed** | **Already diagnosed** | **Potential Undiagnosed** |
| --- | --- | --- | --- |
| Record of smoking status in the 12-month period before January 1st |  |  | 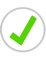 |
| Proportion of patients with **COPD review*** within 6 weeks of respiratory hospitalization in the 12-month period before January 1st |  | 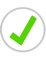 | 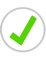 |
| Proportion of patients with a **cardiac risk** assessment 12 months before or after first COPD diagnostic code (newly diagnosed patents) or 12 months before or after 1 January (already/undiagnosed patients) | 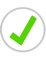 | 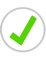 | 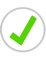 |
| Proportion of patients with **mMRC** recorded in the 12-month period before or after 1 January |  |  | 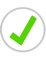 |
| Proportion of patients with **CAT** recorded in the 12-month period before or after first COPD diagnostic code | 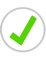 |  |  |
| Proportion of patients with **spirometry** (i.e., FEV_1_, FVC or FEV_1_/FVC) assessed in the 12-month period before first COPD diagnostic code (new diagnosed patients) or in the 12-month period before or after 1 January (already diagnosed) | 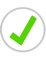 | 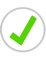 |  |
| Proportion of patients with an **exacerbation history review** 12 months before or after the first COPD diagnostic code (newly diagnosed patients) or before or after 1 January (already diagnosed patents) | 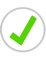 | 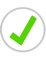 |  |
| Proportion of patients with a **COPD medication review** within 6 months of a treatment change in the 12-month period after first COPD diagnostic code | 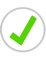 |  |  |
| Proportion of patients with mMRC ≥2 within 12 months before or after first COPD diagnostic code (newly diagnosed patents) or before or after 1 January (already diagnosed) **offered or referred for pulmonary rehabilitation** within 12 months of mMRC score | 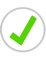 | 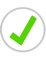 |  |
| Proportion of patients who had ever **received pneumococcal vaccination** before 1 January |  | 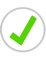 |  |
| Proportion of patients who received the **influenza vaccination** in the 12-month period before or after 1 January |  | 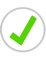 |  |
| **Abbreviations:** CAT: COPD assessment test; COPD: chronic obstructive pulmonary disease; FEV_1_: forced expiratory volume in 1 second; FVC: forced vital capacity; mMRC: modified Medical Research Council * includes assessment of mMRC, CAT, spirometry, annual review, self-management, exacerbation count, medication, inhaler technique and pulmonary rehabilitation | | | |

**S-Figure 1: Study design**


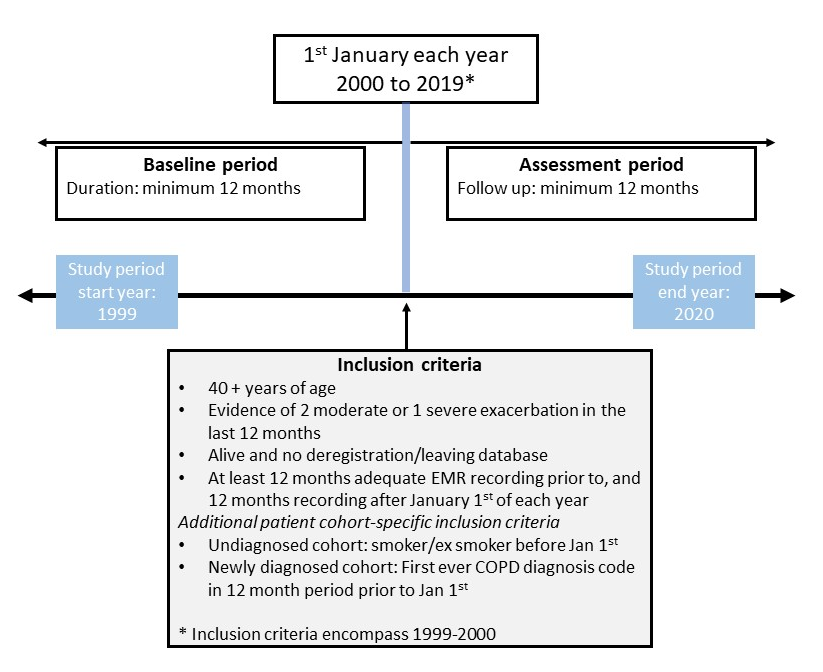


COPD: chronic obstructive pulmonary disease; EMR: electronic medical record

**S-Figure 2: Timeline for outcome assessment relative to index date**

**A: Newly diagnosed cohort**

**
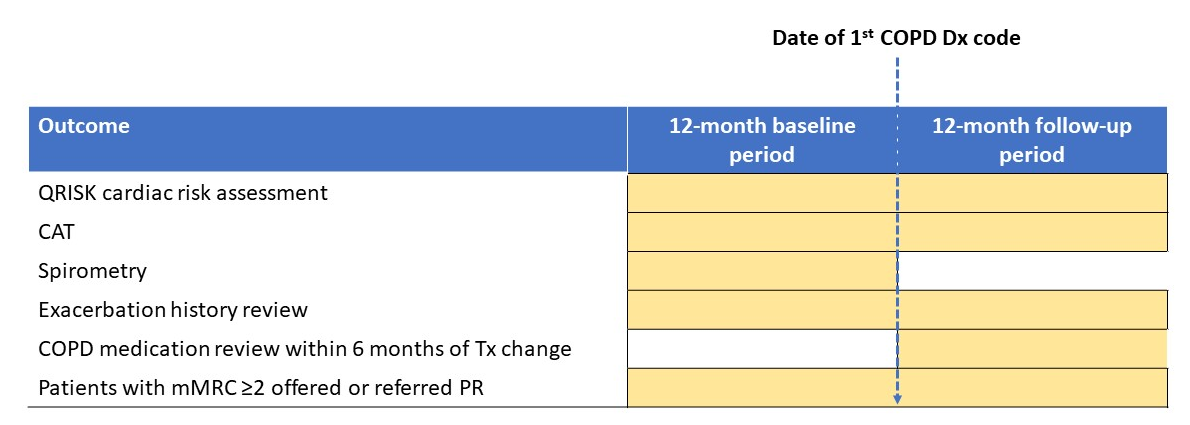
**

**B: Already diagnosed cohort**

**
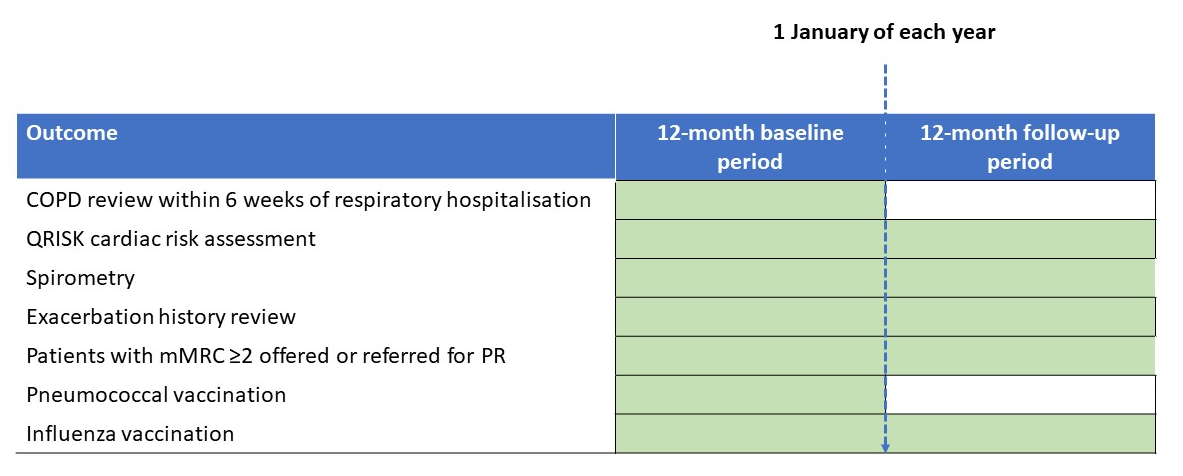
**

**C: Potential undiagnosed cohort**

**
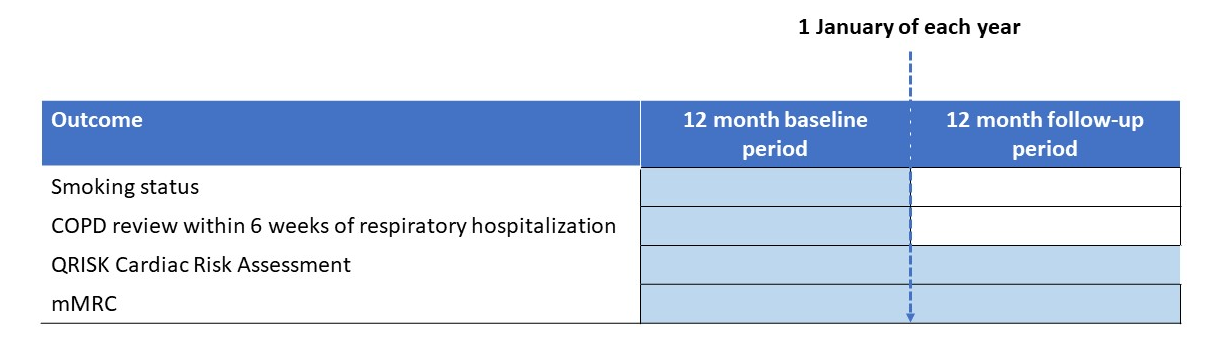
*** COPD review includes assessment of mMRC, CAT, spirometry, annual review, self-management, exacerbation count, medication, inhaler technique and pulmonary rehabilitation. CAT: COPD Assessment Test; COPD: chronic obstructive pulmonary disease; Dx: diagnosis; mMRC: Modified Medical Research Council Dyspnoea Scale; PR: pulmonary rehabilitation; Tx: treatment.

**Results**

**S-Figure 3**: Proportion of eligible patients classified as high-risk COPD over time. N numbers for each patient cohort are provided in **S-Table-3.** Analysis of spirometry data was based on valid entries in any of the following indicators: FEV_1_, FVC, FEV_1_/FVC. Eligible patient: patients aged ≥40 years with a COPD diagnosis, and those who did not have a COPD diagnosis, but had a history of smoking and COPD-like exacerbations. High-risk patients were those with ≥2 moderate or ≥1 severe (hospitalised) exacerbations in the last 12 months.


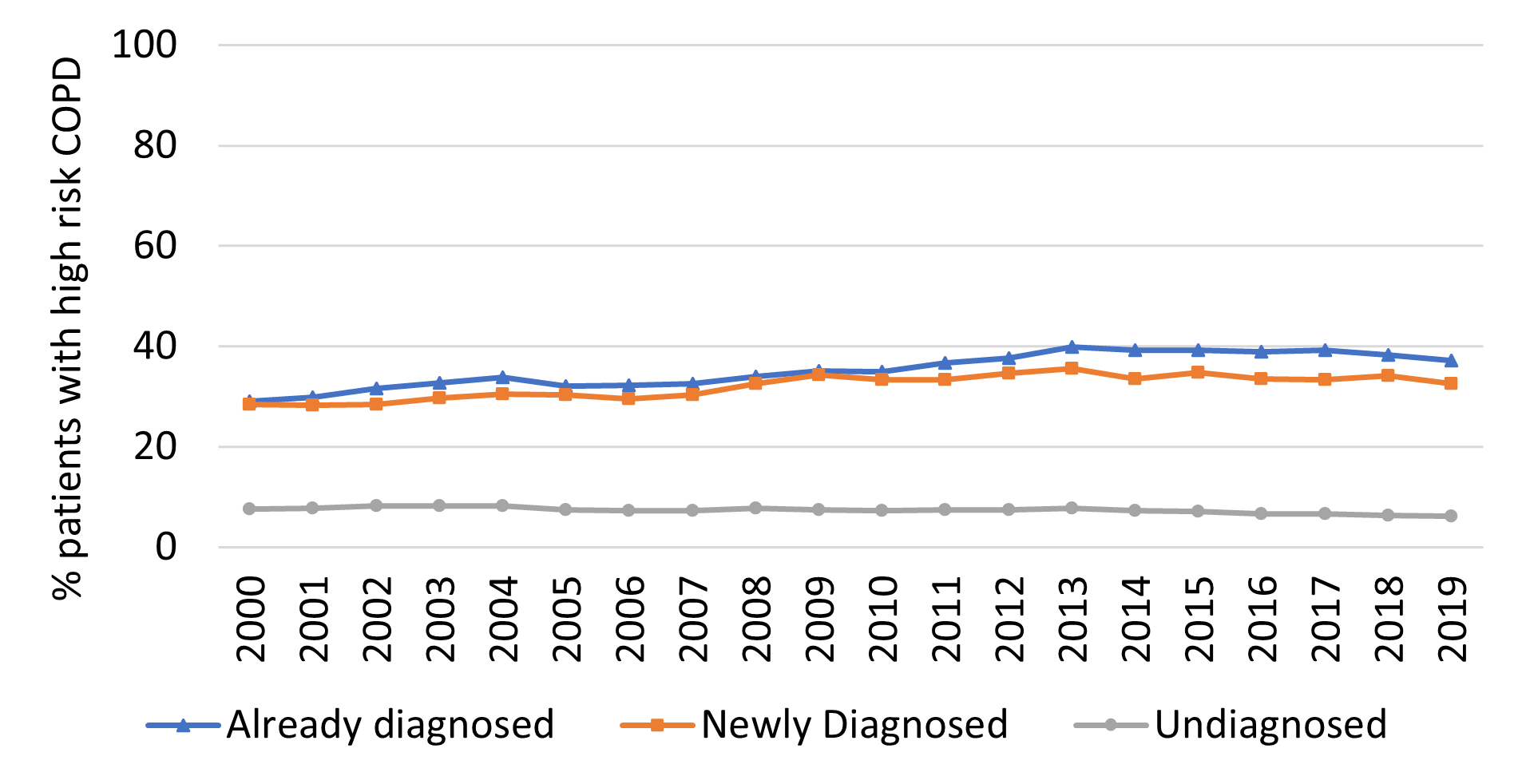


**S-Figure 4: Time point at which patients first meet high-risk criteria (2015-2019)**


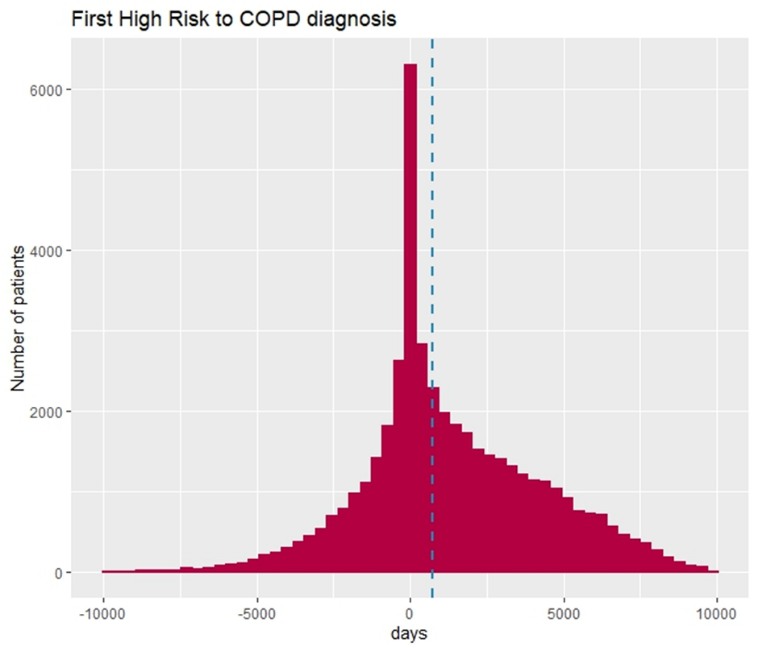


**S-Table 3 Eligible and high-risk patient numbers by year**

| **cohort** | | **2000** | | | **2001** | | **2002** | | **2003** | | **2004** | | **2005** | | **2006** | | **2007** | |  | | **2008** | | **2009** | | **2010** | | **2011** | | **2012** | | **2013** | | **2014** | | **2015** | | **2016** | | **2017** | | **2018** | **2019** |
| --- | --- | --- | --- | --- | --- | --- | --- | --- | --- | --- | --- | --- | --- | --- | --- | --- | --- | --- | --- | --- | --- | --- | --- | --- | --- | --- | --- | --- | --- | --- | --- | --- | --- | --- | --- | --- | --- | --- | --- | --- | --- | --- |
|  | | | **Eligible patients (primary care population)*** | | | | | | | | | | | | | | | | | | | | | | | | | | | | | | | | | | | | | | | |
| **ND** | 2326 | | | 2761 | | 2726 | | 2911 | | 2962 | | 3522 | | 3467 | | 3336 | |  | | 3310 | | 3319 | | 3497 | | 3719 | | 4074 | | 4178 | | 3959 | | 3975 | | 4136 | | 4105 | | 4239 | | 4136 |
| **AD** | 15747 | | | 17803 | | 19990 | | 21662 | | 22634 | | 22031 | | 25252 | | 27733 | |  | | 29680 | | 31601 | | 33438 | | 35120 | | 36807 | | 37827 | | 39965 | | 42280 | | 43731 | | 45184 | | 46918 | | 48063 |
| **PUD** | 212776 | | | 235207 | | 266449 | | 291691 | | 313723 | | 338730 | | 358353 | | 375381 | |  | | 393878 | | 410577 | | 425421 | | 435970 | | 445644 | | 451209 | | 459881 | | 468749 | | 476963 | | 484924 | | 496353 | | 506894 |
|  | | | **High risk patients (analysis sample)†** | | | | | | | | | | | | | | | | | | | | | | | | | | | | | | | | | | | | | | | |
| **ND** | 664 | | | 780 | | 776 | | 865 | | 902 | | 1071 | | 1026 | | 1011 | |  | | 1079 | | 1140 | | 1165 | | 1241 | | 1409 | | 1486 | | 1328 | | 1384 | | 1384 | | 1371 | | 1448 | | 1343 |
| **%‡** | 28.5 | | | 28.3 | | 28.5 | | 29.7 | | 30.5 | | 30.4 | | 29.6 | | 30.3 | |  | | 32.6 | | 34.3 | | 33.3 | | 33.4 | | 34.6 | | 35.6 | | 33.5 | | 34.8 | | 33.5 | | 33.4 | | 34.2 | | 32.5 |
| **AD** | 4585 | | | 5297 | | 6322 | | 7097 | | 7677 | | 7064 | | 8156 | | 9045 | |  | | 10084 | | 11085 | | 11693 | | 12902 | | 13854 | | 15111 | | 15713 | | 16607 | | 17049 | | 17757 | | 17966 | | 17858 |
| **%‡** | 29.1 | | | 29.8 | | 31.6 | | 32.8 | | 33.9 | | 32.1 | | 32.3 | | 32.6 | |  | | 34.0 | | 35.1 | | 35.0 | | 36.7 | | 37.6 | | 39.9 | | 39.3 | | 39.3 | | 39.0 | | 39.3 | | 38.3 | | 37.2 |
| **PUD** | 16253 | | | 18073 | | 21997 | | 23864 | | 25644 | | 25165 | | 26068 | | 27246 | |  | | 30172 | | 30931 | | 31105 | | 32809 | | 32833 | | 35060 | | 33489 | | 33510 | | 31795 | | 31883 | | 31323 | | 31205 |
| **%‡** | 7.6 | | | 7.7 | | 8.3 | | 8.2 | | 8.2 | | 7.4 | | 7.3 | | 7.3 | |  | | 7.7 | | 7.5 | | 7.3 | | 7.5 | | 7.4 | | 7.8 | | 7.3 | | 7.1 | | 6.7 | | 6.6 | | 6.3 | | 6.2 |

AD: already diagnosed; ND: newly diagnosed; PUD: potential undiagnosed

* patients aged ≥40 years who already had a COPD diagnosis, and those who did not have a COPD diagnosis, but had a history of smoking and COPD-like exacerbations (i.e., exacerbations of potential COPD). †Patients with COPD (or potential COPD) who have had 2 or more moderate, or 1 or more severe exacerbations in the last 12 months. ‡ of eligible population;

**S-Table 4: High risk patient characteristics (2000-2004)**

|  | **Newly diagnosed** | | **Already diagnosed** | **Undiagnosed** | | **p value** | |
| --- | --- | --- | --- | --- | --- | --- | --- |
| **N** | 3987 | 30978 | | | 105831 | |  |
| **Age by index date, mean (SD)** | 68.2 (10.6) | 70.8 (10.4) | | | 59.0 (12.5) | | <0.001 |
| **Age by index date, n (%)** | | | | | | | |
| 40 - 49 years | 196 (4.9) | 941 (3.0) | | | 28758 (27.2) | | <0.001 |
| 50 - 59 years | 652 (16.4) | 3647 (11.8) | | | 29372 (27.8) | |  |
| 60 - 69 years | 1240 (31.1) | 8274 (26.7) | | | 24272 (22.9) | |  |
| 70+ years | 1899 (47.6) | 18116 (58.5) | | | 23429 (22.1) | |  |
| **Female, n (%)** | 1910 (47.9) | 14785 (47.7) | | | 61978 (58.6) | | <0.001 |
| **Ethnicity, n (%)** | | | | | | | |
| White | 1557 (39.1) | 11111 (35.9) | | | 55561 (52.5) | | <0.001 |
| Mixed/ Multiple ethnic groups | <5 | 8 (0.0) | | | 204 (0.2) | |  |
| Asian / Asian British | 24 (0.6) | 171 (0.6) | | | 1118 (1.1) | |  |
| Black/ African/ Caribbean/ Black British | 6 (0.2) | 12 (0.04) | | | 203 (0.2) | |  |
| Other ethnic group | 312 (7.8) | 2219 (7.2) | | | 5487 (5.2) | |  |
| Missing ethnicity | 2085 (52.3) | 17457 (56.4) | | | 43258 (40.9) | |  |
| **Smoking, n (%)** | | | | | | | |
| Never-smoker | 260 (6.5) | 2244 (7.2) | | | 0 (0.0) | | <0.001 |
| Current smoker | 1746 (43.8) | 11502 (37.1) | | | 76532 (72.3) | |  |
| Former smoker | 1461 (36.6) | 14268 (46.1) | | | 29299 (27.7) | |  |
| Missing smoking status | 520 (13.04) | 2964 (9.6) | | | 0 (0.0) | |  |
| **BMI (within 5 years of index date), n (%)** | | | | | | | |
| Underweight (<18.5) | 191 (4.8) | 1748 (5.6) | | | 1977 (1.9) | | <0.001 |
| Normal weight (18.5-24) | 1310 (32.9) | 9887 (31.9) | | | 27516 (26.0) | |  |
| Overweight (25-29) | 1019 (25.6) | 7756 (25.04) | | | 31092 (29.4) | |  |
| Obese (30.0+) | 706 (17.7) | 5639 (18.2) | | | 25573 (24.2) | |  |
| Missing BMI | 761 (19.1) | 5948 (19.2) | | | 19673 (18.6) | |  |
| **BEC within 5 years of index date, mean (SD) count 10^9/L** | 0.2 (0.2) | 0.2 (0.2) | | | 0.2 (0.2) | | <0.001 |
| **BEC within 5 years of index date - highest recorded; n (%)** | | | | | | | |
| No BEC recorded in period | 943 (23.7) | 6440 (20.8) | | | 24715 (23.4) | | <0.001 |
| <0.15 (10^9/L) | 1180 (29.6) | 9257 (29.9) | | | 31038 (29.3) | |  |
| 0.15 <0.30 (10^9/L) | 959 (24.1) | 7863 (25.4) | | | 28190 (26.6) | |  |
| 0.30 <0.45 (10^9/L) | 591 (14.8) | 4999 (16.1) | | | 16332 (15.4) | |  |
| >= 0.45 (10^9/L) | 314 (7.9) | 2419 (7.8) | | | 5556 (5.3) | |  |
| **Number of moderate exacerbations in baseline 12m, Mean (SD)** | 3.2 (2.1) | 3.7 (2.5) | | | 2.7 (1.7) | | <0.001 |
| OCS prescriptions | 0.6 (1.7) | 0.9 (2.1) | | | 0.3 (1.3) | | <0.001 |
| Antibiotic prescriptions | 2.2 (1.7) | 2.2 (1.9) | | | 2.3 (1.5) | | <0.001 |
| OCS and Antibiotic prescriptions | 0.4 (0.8) | 0.6 (1.1) | | | 0.1 (0.3) | | <0.001 |
| **Moderate exacerbations in baseline 12m, n (%)** | | | | | | | |
| 0 | 78 (2.0) | 451 (1.5) | | | 949 (0.9) | | <0.001 |
| 1 | 270 (6.8) | 1733 (5.6) | | | 11607 (11.0) | |  |
| 2 | 1518 (38.1) | 11235 (36.3) | | | 57306 (54.2) | |  |
| 3 | 965 (24.2) | 6223 (20.1) | | | 18866 (17.8) | |  |
| 4+ | 1156 (29.0) | 11336 (36.6) | | | 17103 (16.2) | |  |
| **Number of severe exacerbations (hospital admittance for respiratory reason) in baseline 12m, mean (SD)** | 0.1 (0.3) | 0.1 (0.3) | | | 0.02 (0.1) | | <0.001 |
| **Severe exacerbations in baseline 12m, n (%)** | | | | | | | |
| 0 | 3742 (93.9) | 29455 (95.1) | | | 104185 (98.4) | | <0.001 |
| 1 | 231 (5.8) | 1375 (4.4) | | | 1613 (1.5) | |  |
| 2+ | 14 (0.4) | 148 (0.5) | | | 33 (0.03) | |  |
| **Number of moderate exacerbations in follow-up 12m, Mean (SD)** | 2.4 (2.6) | 3.1 (3.0) | | | 1.5 (2.2) | | <0.001 |
| OCS prescriptions | 0.6 (1.8) | 0.8 (2.2) | | | 0.2 (1.2) | | <0.001 |
| Antibiotic prescriptions | 1.5 (1.8) | 1.8 (2.1) | | | 1.3 (1.8) | | <0.001 |
| OCS and Antibiotic prescriptions | 0.3 (0.8) | 0.5 (1.1) | | | 0.1 (0.3) | | <0.001 |
| **Moderate exacerbations in follow-up 12m, n (%)** | | | | | | | |
| 0 | 963 (24.2) | 5530 (17.9) | | | 42780 (40.4) | | <0.001 |
| 1 | 925 (23.2) | 5930 (19.1) | | | 27028 (25.5) | |  |
| 2 | 670 (16.8) | 5089 (16.4) | | | 14940 (14.1) | |  |
| 3 | 456 (11.4) | 3988 (12.9) | | | 8002 (7.6) | |  |
| 4+ | 973 (24.4) | 10441 (33.7) | | | 13081 (12.4) | |  |
| **Number of severe exacerbations (hospital admittance for respiratory reason) in follow-up 12m, mean (SD)** | 0.03 (0.2) | 0.1 (0.3) | | | 0.01 (0.1) | | <0.001 |
| **Severe exacerbations in follow-up 12m, n (%)** | | | | | | | |
| 0 | 3871 (97.1) | 29805 (96.2) | | | 105294 (99.5) | | <0.001 |
| 1 | 109 (2.7) | 1005 (3.2) | | | 516 (0.5) | |  |
| 2+ | 7 (0.2) | 168 (0.5) | | | 21 (0.02) | |  |
| **Number of rescue inhaler prescriptions in baseline 12m, mean (SD)** | 5.2 (7.6) | 9.7 (10.0) | | | 1.0 (3.3) | | <0.001 |
| SABA | 3.6 (5.0) | 5.8 (6.0) | | | 0.8 (2.6) | | <0.001 |
| SAMA | 0.9 (2.9) | 2.5 (4.6) | | | 0.1 (0.9) | | <0.001 |
| SABA/SAMA | 0.7 (2.6) | 1.4 (3.7) | | | 0.1 (0.8) | | <0.001 |
| **Number of rescue inhaler prescriptions in follow-up 12m, mean (SD)** | 7.9 (9.01) | 10.0 (10.2) | | | 1.1 (3.6) | | <0.001 |
| SABA | 4.8 (5.4) | 5.9 (6.1) | | | 0.9 (2.8) | | <0.001 |
| SAMA | 1.7 (3.9) | 2.6 (4.7) | | | 0.1 (1.02) | | <0.001 |
| SABA/SAMA | 1.3 (3.5) | 1.4(3.8) | | | 0.1 (0.1) | | <0.001 |
| **Major cardiac events in baseline 12m, n(%)** | | | | | | | |
| New diagnosis for heart failure | 175 (4.4) | 1192 (3.9) | | | 1231 (1.2) | | <0.001 |
| Hospitalisation for heart failure | 511 (12.8) | 4320 (14.0) | | | 8909 (8.4) | | <0.001 |
| Revascularization | 12 (0.3) | 49 (0.2) | | | 296 (0.3) | | <0.01 |
| Myocardial Infarction | 61 (1.5) | 449 (1.5) | | | 1014 (1.0) | | <0.001 |
| Stroke | 43 (1.1) | 500 (1.6) | | | 903 (0.9) | | <0.001 |
| Any of above | 714 (17.9) | 5767 (18.6) | | | 11307 (10.7) | | <0.001 |
| **Major cardiac events in follow-up 12m, n (%)** | | | | | | | |
| New diagnosis for heart failure | 241 (6.0) | 1174 (3.8) | | | 1244 (1.2) | | <0.001 |
| Hospitalisation for heart failure | 535 (13.4) | 4738 (15.3) | | | 8735 (8.3) | | <0.001 |
| Revascularization | 7 (0.2) | 57 (0.2) | | | 218 (0.2) | | 0.70 |
| Myocardial Infarction | 71 (1.8) | 472 (1.5) | | | 946 (0.9) | | <0.001 |
| Stroke | 52 (1.3) | 568 (1.8) | | | 1073 (1.01) | | <0.001 |
| Any of above | 778 (19.5) | 6189 (20.0) | | | 11217 (10.6) | | <0.001 |
| **MRC dyspnoea score recorded in 12 months before index date, mean (SD)** | 2.5 (0.9) | 2.5 (0.9) | | | 2.3 (0.9) | | <0.001 |
| No MRC score, N (%) | 3401 (85.3) | 27503 (88.8) | | | 102740 (97.1) | | <0.001 |
| **MRC dyspnoea score recorded in 12 months before index date, n (%)** | | | | | | | |
| 1-2 | 402 (68.6) | 2382 (68.6) | | | 2328 (75.3) | | <0.001 |
| 3-5 | 184 (31.4) | 1093 (31.5) | | | 763 (24.7) | |  |
| **Spirometry values recorded in 12 months before index date, mean (SD)** | | | | | | | |
| FEV_1_ % predicted | 56.5 (17.2) | 50.1 (20.2) | | | 80.3 (21.3) | | <0.001 |
| FEV_1_; litres | 1.5 (0.6) | 1.2 (0.6) | | | 2.2 (0.8) | | <0.001 |
| FVC; litres | 2.5 (0.8) | 2.2 (0.8) | | | 2.9 (0.9) | | <0.001 |
| FEV_1_/FVC | 0.6 (0.1) | 0.5 (0.2) | | | 0.7 (0.1) | | <0.001 |
| % No spirometry recorded | 3309 (83.0) | 28313 (91.4) | | | 105054 (99.3) | | <0.001 |
| **COPD therapy in baseline 12m, n (%)** | | | | | | | |
| No COPD therapy | 901 (22.6) | 4245 (13.7) | | | 83635 (79.03) | | <0.001 |
| Reliever only (SABA, SAMA and combinations) | 874 (21.9) | 3973 (12.8) | | | 8296 (7.8) | |  |
| ICS only (mono) | 1455 (36.5) | 11826 (38.2) | | | 10027 (9.5) | |  |
| LABA only (mono) | 75 (1.9) | 1060 (3.4) | | | 290 (0.3) | |  |
| LAMA only (mono) | 12 (0.3) | 82 (0.3) | | | 14 (0.01) | |  |
| LABA-ICS (Dual) | 428 (10.7) | 5884 (19.0) | | | 2092 (2.0) | |  |
| LABA-ICS fixed (Dual) | 175 (4.4) | 2538 (8.2) | | | 1156 (1.1) | |  |
| LABA-LAMA (dual) | <5 | 51 (0.2) | | | 0 (0.0) | |  |
| LABA-LAMA fixed (Dual) | 0 (0.0) | 0 (0.0) | | | 0 (0.0) | |  |
| LAMA-ICS (dual) | 8 (0.2) | 163 (0.5) | | | 11 (0.01) | |  |
| LABA-LAMA-ICS (triple) | 13 (0.3) | 529 (1.7) | | | 17 (0.02) | |  |
| LABA-LAMA-ICS fixed (triple) | 0 (0.0) | 0 (0.0) | | | 0 (0.0) | |  |
| **Clinically diagnosed comorbidities (ever), n(%)** | | | | | | | |
| **Steroid related** | | | | | | | |
| Diabetes type 2 | 232 (5.8) | 2216 (7.2) | | | 8220 (7.8) | | <0.001 |
| Osteoporosis | 168 (4.2) | 1798 (5.8) | | | 2150 (2.0) | | <0.001 |
| Hypertension | 1087 (27.3) | 8754 (28.3) | | | 24559 (23.2) | | <0.001 |
| Chronic kidney disease | 46 (1.2) | 415 (1.3) | | | 993 (0.9) | | <0.001 |
| Depression/Anxiety | 885 (22.2) | 8297 (26.8) | | | 30142 (28.5) | | <0.001 |
| Obesity | 680 (17.1) | 6164 (19.9) | | | 25953 (24.5) | | <0.001 |
| **Other** | | | | | | | |
| CVD (ischaemic or coronary) | 802 (20.1) | 7353 (23.7) | | | 13749 (13.0) | | <0.001 |
| Prior asthma | 949 (23.8) | 11967 (38.6) | | | 13284 (12.6) | | <0.001 |
| OSA | 17 (0.4) | 172 (0.6) | | | 402 (0.4) | | <0.001 |
| GERD | 320 (8.03) | 3517 (11.4) | | | 9248 (8.7) | | <0.001 |
| Lung cancer | 19 (0.5) | 186 (0.6) | | | 205 (0.2) | | <0.001 |
| Anaemia | 104 (2.6) | 1126 (3.6) | | | 2707 (2.6) | | <0.001 |
| **Cambridge multimorbidity score, mean (SD)** | 2.3 (1.1) | 2.5 (1.2) | | | 0.7 (0.9) | | <0.001 |
| **Hospital admission for any condition, mean (SD)** | | | | | | | |
| In baseline 12m | 0.2 (0.6) | 0.2 (0.7) | | | 0.1 (0.5) | | <0.001 |
| In follow-up 12m | 0.2 (0.6) | 0.3 (0.8) | | | 0.1 (0.5) | | <0.001 |

BEC: blood eosinophil count; BMI: body mass index; COPD: chronic obstructive pulmonary disease; CVD: cardiovascular disease; FEV_1_: forced expiratory volume in one second; FVC: forced vital capacity; GERD: gastroesophageal reflux disease; ICS: inhaled corticosteroid; LABA: long-acting β2-agonist; LAMA: long-acting muscarinic antagonist; MRC: Medical Research Council; OCS: oral corticosteroid; OSA: obstructive sleep apnoea; SABA: short-acting β_2_-agonist; SAMA: short-acting muscarinic antagonist; SD: standard deviation

The baseline/follow up periods refer to 12 months pre/post index date. The 5-year periods were generated by aggregating all the records in each individual year and treating them as separate observations. Patients in individual years (2000-2004) were added together into one combined data set covering the whole 5-year period. Patients could be present in multiple years during that period and contribute multiple records to the aggregated analysis. Each patient index date combination was treated as an independent observation.

**S-Table 5: Patient characteristics (2005-2009)**

|  | **Newly diagnosed** | **Already diagnosed** | **Undiagnosed** | **p value** |
| --- | --- | --- | --- | --- |
| **N** | 5327 | 45434 | 139582 |  |
| **Age by index date, mean (SD)** | 68.3 (11.0) | 71.4 (10.6) | 59.6 (13.0) | <0.001 |
| **Age by index date, n (%)** | | | | |
| 40 - 49 years | 267 (5.0) | 1302 (2.9) | 38436 (27.5) | <0.001 |
| 50 - 59 years | 888 (16.7) | 4735 (10.4) | 35072 (25.1) |  |
| 60 - 69 years | 1631 (30.6) | 12735 (28.0) | 32522 (23.3) |  |
| 70+ years | 2541 (47.7) | 26662 (58.7) | 33552 (24.0) |  |
| **Female, n (%)** | 2580 (48.4) | 22582 (49.7) | 79803 (57.2) | <0.001 |
| **Ethnicity, n (%)** | | | | |
| White | 3066 (57.6) | 25381 (55.9) | 81234 (58.2) | <0.001 |
| Mixed/ Multiple ethnic groups | <5 | 33 (0.1) | 406 (0.3) |  |
| Asian / Asian British | 55 (1.0) | 407 (0.9) | 3108 (2.2) |  |
| Black/ African/ Caribbean/ Black British | 6 (0.1) | 45 (0.1) | 413 (0.3) |  |
| Other ethnic group | 399 (7.5) | 2887 (6.4) | 7760 (5.6) |  |
| Missing ethnicity | 1797 (33.7) | 16681 (36.7) | 46661 (33.4) |  |
| **Smoking, n (%)** | | | | |
| Never-smoker | 277 (5.2) | 2660 (5.9) | 0.0(0.0) | <0.001 |
| Current smoker | 2193 (41.2) | 13517 (29.8) | 70722 (50.7) |  |
| Former smoker | 2731 (51.3) | 28561 (62.9) | 68860 (49.3) |  |
| Missing smoking status | 126 (2.4) | 696 (1.5) | 0.0 (0.0) |  |
| **BMI (within 5 years of index date), n (%)** | | | | |
| Underweight (<18.5) | 252 (4.7) | 2866 (6.3) | 2463 (1.8) | <0.001 |
| Normal weight (18.5-24) | 1816 (34.1) | 15440 (34.0) | 35148 (25.2) |  |
| Overweight (25-29) | 1636 (30.7) | 13558 (29.8) | 43401 (31.1) |  |
| Obese (30.0+) | 1324 (24.9) | 10649 (23.4) | 41002 (29.4) |  |
| Missing BMI | 299 (5.6) | 2921 (6.4) | 17568 (12.6) |  |
| **BEC within 5 years of index date, mean (SD) count 10^9/L** | 0.2 (0.2) | 0.2 (0.2) | 0.2 (0.2) | <0.001 |
| **BEC within 5 years of index date - highest recorded; n (%)** | | | | |
| No BEC recorded in period | 376 (7.1) | 2885 (6.4) | 13510 (9.7) | <0.001 |
| <0.15 (10^9/L) | 1692 (31.8) | 14961 (32.9) | 46009 (33.0) |  |
| 0.15 <0.30 (10^9/L) | 1639 (30.8) | 14454 (31.8) | 45735 (32.8) |  |
| 0.30 <0.45 (10^9/L) | 1139 (21.4) | 8903 (19.6) | 25493 (18.3) |  |
| ≥ 0.45 (10^9/L) | 481 (9.0) | 4231 (9.3) | 8835 (6.3) |  |
| **Number of moderate exacerbations in baseline 12m, Mean (SD)** | 2.8 (1.8) | 3.57 (2.5) | 2.7 (1.8) | <0.001 |
| OCS prescriptions | 0.3 (1.2) | 0.6 (1.8) | 0.2 (1.2) | <0.001 |
| Antibiotic prescriptions | 2.2 (1.6) | 2.2 (1.9) | 2.4 (1.6) | <0.001 |
| OCS and Antibiotic prescriptions | 0.3 (0.7) | 0.8 (1.3) | 0.1 (0.3) | <0.001 |
| **Moderate exacerbations in baseline 12m, n (%)** | | | | |
| 0 | 202 (3.8) | 1089 (2.4) | 1776 (1.3) | <0.001 |
| 1 | 432 (8.1) | 2692 (5.9) | 15471 (11.1) |  |
| 2 | 2309 (43.4) | 16307 (35.9) | 76221 (54.6) |  |
| 3 | 1215 (22.8) | 9201 (20.3) | 23877 (17.1) |  |
| 4+ | 1169 (21.9) | 16145 (35.5) | 22237 (15.9) |  |
| **Number of severe exacerbations (hospital admittance for respiratory reason) in baseline 12m, mean (SD)** | 0.1 (0.3) | 0.1 (0.3) | 0.02 (0.2) | <0.001 |
| **Severe exacerbations in baseline 12m, n (%)** | | | | |
| 0 | 4832 (90.7) | 41926 (92.3) | 136448 (97.8) | <0.001 |
| 1 | 472 (8.9) | 3181 (7.0) | 3064 (2.2) |  |
| 2+ | 23 (0.4) | 327 (0.7) | 70 (0.1) |  |
| **Number of moderate exacerbations in follow-up 12m, mean (SD) [removed duplicates in same 7-day period]** | 1.9 (2.2) | 3.0 (3.0) | 1.5 (2.3) | <0.001 |
| OCS prescriptions | 0.3 (1.2) | 0.6 (1.8) | 0.2 (1.2) | <0.001 |
| Antibiotic prescriptions | 1.3 (1.7) | 1.7 (2.1) | 1.3 (2.0) | <0.001 |
| OCS and Antibiotic prescriptions | 0.3 (0.8) | 0.7 (1.4) | 0.04 (0.3) | <0.001 |
| **Moderate exacerbations in follow-up 12m, n (%)** | | | | |
| 0 | 1606 (30.2) | 8210 (18.1) | 58392 (41.8) | <0.001 |
| 1 | 1371 (25.7) | 8842 (19.5) | 35425 (25.4) |  |
| 2 | 875 (16.4) | 7595 (16.7) | 18810 (13.5) |  |
| 3 | 558 (10.5) | 5808 (12.8) | 9765 (7.0) |  |
| 4+ | 917 (17.2) | 14979 (33.0) | 17190 (12.3) |  |
| **Number of severe exacerbations (hospital admittance for respiratory reason) in follow-up 12m, mean (SD)** | 0.04 (0.2) | 0.1 (0.3) | 0.01 (0.1) | <0.001 |
| **Severe exacerbations in follow-up 12m, n (%)** | | | | |
| 0 | 5129 (96.3) | 43023 (94.7) | 138586 (99.3) | <0.001 |
| 1 | 187 (3.5) | 2089 (4.6) | 960 (0.7) |  |
| 2+ | 11 (0.2) | 322 (0.7) | 36 (0.03) |  |
| **Number of rescue inhaler prescriptions in baseline 12m, mean (SD)** | 3.3 (5.2) | 8.8 (8.8) | 0.6 (2.2) | <0.001 |
| SABA | 2.5 (3.8) | 6.1 (5.8) | 0.5 (1.9) | <0.001 |
| SAMA | 0.4 (1.7) | 1.6 (3.6) | 0.03 (0.5) | <0.001 |
| SABA/SAMA | 0.4 (1.7) | 1.2 (3.2) | 0.03 (0.5) | <0.001 |
| **Number of rescue inhaler prescriptions in follow-up 12m, mean (SD)** | 5.8 (6.9) | 8.4 (8.5) | 0.7 (2.5) | <0.001 |
| SABA | 4.03 (4.6) | 6.0 (5.8) | 0.6 (2.1) | <0.001 |
| SAMA | 0.9 (2.7) | 1.6 (3.6) | 0.1 (0.6) | <0.001 |
| SABA/SAMA | 0.8 (2.5) | 0.8 (2.8) | 0.04 (0.5) | <0.001 |
| **Major cardiac events in baseline 12m, n (%)** | | | | |
| New diagnosis for heart failure | 178 (3.3) | 1266 (2.8) | 1392 (1.0) | <0.001 |
| Hospitalisation for heart failure | 950 (17.8) | 8934 (19.7) | 17419 (12.5) | <0.001 |
| Revascularization | 11 (0.2) | 62 (0.1) | 305 (0.2) | <0.01 |
| Myocardial Infarction | 77 (1.5) | 626 (1.4) | 1235 (0.9) | <0.001 |
| Stroke | 78 (1.5) | 751 (1.7) | 1381 (1.0) | <0.001 |
| Any of above | 1143 (21.5) | 10533 (23.2) | 20060 (14.4) | <0.001 |
| **Major cardiac events in follow-up 12m, n (%)** | | | | |
| New diagnosis for heart failure | 213 (4.0) | 1308 (2.9) | 1466 (1.1) | <0.001 |
| Hospitalisation for heart failure | 938 (17.6) | 9659 (21.3) | 17423 (12.5) | <0.001 |
| Revascularization | 8 (0.2) | 46 (0.1) | 197 (0.1) | 0.12 |
| Myocardial Infarction | 74 (1.4) | 609 (1.3) | 1162 (0.8) | <0.001 |
| Stroke | 76 (1.4) | 735 (1.6) | 1415 (1.01) | <0.001 |
| Any of above | 1163 (21.8) | 11213 (24.7) | 20103 (14.4) | <0.001 |
| **MRC dyspnoea score recorded in 12 months before index date, mean (SD)** | 2.4 (0.9) | 2.7 (1.03) | 2.2 (0.8) | <0.001 |
| No MRC score, n (%) | 3646 (68.4) | 31247 (68.8) | 133921 (95.9) | <0.001 |
| **MRC dyspnoea score recorded in 12 months before index date, n (%)** | | | | |
| 1-2 | 1097 (65.3) | 7165 (50.5) | 4267 (75.4) | <0.001 |
| 3-5 | 584 (34.7) | 7022 (49.5) | 1394 (24.6) |  |
| **Spirometry values recorded in 12 months before index date, mean (SD)** | | | | |
| FEV_1_ % predicted | 59.71 (17.6) | 53.2 (19.6) | 82.7 (20.1) | <0.001 |
| FEV_1_; litres | 1.55 (0.6) | 1.3 (0.6) | 2.2 (0.8) | <0.001 |
| FVC; litres | 2.59 (0.9) | 2.3 (0.9) | 3.02 (0.9) | <0.001 |
| FEV_1_/FVC | 0.6 (0.1) | 0.6 (0.2) | 0.7 (0.1) | <0.001 |
| % No spirometry recorded | 1998 (37.5) | 20442 (45.0) | 135228 (96.9) | <0.001 |
| **COPD therapy in baseline 12m, n (%)** | | | | |
| No COPD therapy | 1276 (24.0) | 4482 (9.9) | 116344 (83.4) | <0.001 |
| Reliever only (SABA, SAMA and combinations) | 1506 (28.3) | 4641 (10.2) | 11683 (8.4) |  |
| ICS only (mono) | 988 (18.6) | 6191 (13.6) | 7012 (5.02) |  |
| LABA only (mono) | 99 (1.9) | 1078 (2.4) | 188 (0.1) |  |
| LAMA only (mono) | 165 (3.1) | 1126 (2.5) | 116 (0.1) |  |
| LABA-ICS (Dual) | 209 (3.9) | 3570 (7.9) | 879 (0.6) |  |
| LABA-ICS fixed (Dual) | 748 (14.04) | 11195 (24.6) | 2849 (2.04) |  |
| LABA-LAMA (dual) | 13 (0.2) | 435 (1.0) | 9 (0.01) |  |
| LABA-LAMA fixed (Dual) | 0 (0.0) | 0 (0.0) | 0 (0.0) |  |
| LAMA-ICS (dual) | 73 (1.4) | 1101 (2.4) | 59 (0.04) |  |
| LABA-LAMA-ICS (triple) | 227 (4.3) | 11253 (24.8) | 225 (0.2) |  |
| LABA-LAMA-ICS fixed (triple) | 0 (0.0) | 0 (0.0) | 0 (0.0) |  |
| **Clinically diagnosed comorbidities (ever), n (%)** | | | | |
| **Steroid related** | | | | |
| Diabetes type 2 | 552 (10.4) | 5497 (12.1) | 16285 (11.7) | <0.001 |
| Osteoporosis | 280 (5.3) | 3834 (8.4) | 3879 (2.8) | <0.001 |
| Hypertension | 2021 (37.9) | 17661 (38.9) | 42449 (30.4) | <0.001 |
| Chronic kidney disease | 363 (6.8) | 4242 (9.3) | 7787 (5.6) | <0.001 |
| Depression/Anxiety | 1578 (29.6) | 15961 (35.1) | 50242 (36.0) | <0.001 |
| Obesity | 1477 (27.7) | 13588 (29.9) | 44769 (32.1) | <0.001 |
| **Other** | | | | |
| CVD (ischaemic or coronary) | 1232 (23.1) | 12025 (26.5) | 21007 (15.1) | <0.001 |
| Prior asthma | 811 (15.2) | 19001 (41.8) | 13545 (9.7) | <0.001 |
| OSA | 31 (0.6) | 585 (1.3) | 1122 (0.8) | <0.001 |
| GERD | 609 (11.4) | 6973 (15.4) | 16770 (12.01) | <0.001 |
| Lung cancer | 28 (0.5) | 373 (0.8) | 305 (0.2) | <0.001 |
| Anaemia | 218 (4.1) | 2514 (5.5) | 5286 (3.8) | <0.001 |
| **Cambridge multimorbidity Score, mean (SD)** | 2.5 (1.1) | 2.7 (1.3) | 0.8 (1.02) | <0.001 |
| **Hospital admission for any condition, mean (SD)** | | | | |
| In baseline 12m | 0.3 (0.7) | 0.3 (0.8) | 0.2 (0.6) | <0.001 |
| In follow-up 12m | 0.3 (0.8) | 0.4 (0.9) | 0.2 (0.6) | <0.001 |

BEC: blood eosinophil count; BMI: body mass index; COPD: chronic obstructive pulmonary disease; CVD: cardiovascular disease; FEV_1_: forced expiratory volume in one second; FVC: forced vital capacity; GERD: gastroesophageal reflux disease; ICS: inhaled corticosteroid; LABA: long-acting β2-agonist; LAMA: long-acting muscarinic antagonist; MRC: Medical Research Council; OCS: oral corticosteroid; OSA: obstructive sleep apnoea; SABA: short-acting β_2_-agonist; SAMA: short-acting muscarinic antagonist; SD: standard deviation

The baseline/follow up periods refer to 12 months pre/post index date. The 5-year periods were generated by aggregating all the records in each individual year and treating them as separate observations Patients in individual years (2005-2009) were added together into one combined data set covering the whole 5-year period. Patients could be present in multiple years during that period and contribute multiple records to the aggregated analysis. Each patient index date combination was treated as an independent observation.

**S-Table 6: Patient characteristics (2010-2014)**

|  | **Newly diagnosed** | **Already diagnosed** | **Undiagnosed** | **p value** |
| --- | --- | --- | --- | --- |
| **N** | 6629 | 69273 | 165296 |  |
| **Age by index date, mean (SD)** | 67.65 (11.3 | 71.5 (10.5) | 60.4 (13.4) | <0.001 |
| **Age by index date, n (%)** | | | | |
| 40 - 49 years | 404 (6.1) | 1858 (2.7) | 43354 (26.2) | <0.001 |
| 50 - 59 years | 1178 (17.8) | 7230 (10.4) | 40059 (24.2) |  |
| 60 - 69 years | 2134 (32.2) | 20068 (29.0) | 38955 (23.6) |  |
| 70+ years | 2913 (43.9) | 40117 (57.9) | 42928 (26.0) |  |
| **Female, n (%)** | 3293 (49.7 | 34946 (50.5) | 93683 (56.7) | <0.001 |
| **Ethnicity, n (%)** | | | | |
| White | 4448 (67.1) | 47557 (68.7) | 106520 (64.4) | <0.001 |
| Mixed/ Multiple ethnic groups | 17 (0.3) | 107 (0.2) | 647 (0.4) |  |
| Asian / Asian British | 124 (1.9) | 830 (1.2) | 4985 (3.02) |  |
| Black/ African/ Caribbean/ Black British | 8 (0.1) | 88 (0.1) | 673 (0.4) |  |
| Other ethnic group | 395 (6.0) | 3720 (5.4) | 8595 (5.2) |  |
| Missing ethnicity | 1637 (24.7) | 16971 (24.5) | 43876 (26.5) |  |
| **Smoking, n (%)** | | | | |
| Never-smoker | 298 (4.5) | 3162 (4.6) | 0.0 (0.0) | <0.001 |
| Current smoker | 3062 (46.2) | 22879 (33.03) | 74182 (44.9) |  |
| Former smoker | 3190 (48.1) | 42467 (61.3) | 91114 (55.1) |  |
| Missing smoking status | 79 (1.2) | 765 (1.1) | 0.0 (0.0) |  |
| **BMI (within 5 years of index date), n (%)** | | | | |
| Underweight (<18.5) | 303 (4.6) | 3840 (5.5) | 2847 (1.7) | <0.001 |
| Normal weight (18.5-24) | 2058 (31.1) | 22252 (32.1) | 40318 (24.4) |  |
| Overweight (25-29) | 2118 (32.0) | 21804 (31.5) | 52229 (31.6) |  |
| Obese (30.0+) | 1946 (29.4) | 19227 (27.8) | 54157 (32.8) |  |
| Missing BMI | 204 (3.1) | 2150 (3.1) | 15745 (9.5) |  |
| **BEC within 5 years of index date, mean (SD) count 10^9^/L** | 0.2 (0.2) | 0.2 (0.2) | 0.2 (0.1) | <0.001 |
| **BEC within 5 years of index date - highest recorded; n (%)** | | | | |
| No BEC recorded in period | 293 (4.4) | 2336 (3.4) | 9970 (6.03) | <0.001 |
| <0.15 (10^9^/L) | 2100 (31.7) | 23621 (34.1) | 56619 (34.3) |  |
| 0.15 <0.30 (10^9^/L) | 2250 (33.9) | 23118 (33.4) | 56503 (34.2) |  |
| 0.30 <0.45 (10^9^/L) | 1359 (20.5) | 14152 (20.4) | 31321 (19.0) |  |
| ≥ 0.45 (10^9^/L) | 627 (9.5) | 6046 (8.7) | 10883 (6.6) |  |
| **Number of moderate exacerbations in baseline 12m, Mean (SD)** | 2.8 (1.8) | 3.6 (2.6) | 2.7 (2.02) | <0.001 |
| OCS prescriptions | 0.3 (1.01) | 0.5 (1.5) | 0.3 (1.3) | <0.001 |
| Antibiotic prescriptions | 2.2 (1.6) | 2.02 (2.1) | 2.4 (1.8) | <0.001 |
| OCS and Antibiotic prescriptions | 0.4 (0.8) | 1.1 (1.6) | 0.1 (0.4) | <0.001 |
| **Moderate exacerbations in baseline 12m, n (%)** | | | | |
| 0 | 291 (4.4) | 2385 (3.4) | 3138 (1.9) | <0.001 |
| 1 | 540 (8.2) | 5216 (7.5) | 18765 (11.4) |  |
| 2 | 2814 (42.5) | 23111 (33.4) | 87241 (52.8) |  |
| 3 | 1560 (23.5) | 13544 (19.6) | 27628 (16.7) |  |
| 4+ | 1424 (21.5) | 25017 (36.1) | 28524 (17.3) |  |
| **Number of severe exacerbations (hospital admittance for respiratory reason) in baseline 12m, mean (SD)** | 0.1 (0.4) | 0.1 (0.4) | 0.03 (0.2) | <0.001 |
| **Severe exacerbations in baseline 12m, n (%)** | | | | |
| 0 | 5804 (87.6) | 62670 (90.5) | 159729 (96.6) | <0.001 |
| 1 | 778 (11.7) | 5942 (8.6) | 5435 (3.3) |  |
| 2+ | 47 (0.7) | 661 (1.0) | 132 (0.1) |  |
| **Number of moderate exacerbations in follow-up 12m, mean (SD) [removed duplicates in same 7-day period]** | 1.9 (2.3) | 3.2 (3.0) | 1.6 (2.5) | <0.001 |
| OCS prescriptions | 0.2 (1.01) | 0.5 (1.5) | 0.2 (1.3) | <0.001 |
| Antibiotic prescriptions | 1.3 (1.8) | 1.7 (2.2) | 1.4 (2.1) | <0.001 |
| OCS and Antibiotic prescriptions | 0.5 (1.0) | 1.03 (1.7) | 0.1 (0.4) | <0.001 |
| **Moderate exacerbations in follow-up 12m, n (%)** | | | | |
| 0 | 1984 (29.9) | 12091 (17.5) | 68785 (41.6) | <0.001 |
| 1 | 1676 (25.3) | 13108 (18.9) | 40996 (24.8) |  |
| 2 | 1108 (16.7) | 11418 (16.5) | 21614 (13.1) |  |
| 3 | 722 (10.9) | 8865 (12.8) | 11331 (6.9) |  |
| 4+ | 1139 (17.2) | 23791 (34.3) | 22570 (13.7) |  |
| **Number of severe exacerbations (hospital admittance for respiratory reason) in follow-up 12m, mean (SD)** | 0.1 (0.3) | 0.1 (0.3) | 0.01 (0.1) | <0.001 |
| **Severe exacerbations in follow-up 12m, n (%)** | | | | |
| 0 | 6270 (94.6) | 64229 (92.7) | 163439 (98.9) | <0.001 |
| 1 | 330 (5.0) | 4383 (6.3) | 1751 (1.1) |  |
| 2+ | 29 (0.4) | 661 (1.0) | 106 (0.1) |  |
| **Number of rescue inhaler prescriptions in baseline 12m, mean (SD)** | 2.5 (3.8) | 6.9 (6.6) | 0.6 (2.0) | <0.001 |
| SABA | 2.3 (3.4) | 6.0 (5.4) | 0.5 (1.9) | <0.001 |
| SAMA | 0.2 (1.2) | 0.9 (2.9) | 0.03 (0.5) | <0.001 |
| SABA/SAMA | 0.01 (0.1) | 0 (0.1) | 0 (0.01) | <0.001 |
| **Number of rescue inhaler prescriptions in follow-up 12m, mean (SD)** | 4.4 (4.9) | 6.9 (6.5) | 0.6 (2.3) | <0.001 |
| SABA | 4.02 (4.5) | 6.1 (5.5) | 0.6 (2.1) | <0.001 |
| SAMA | 0.4 (1.6) | 0.8 (2.8) | 0.03 (0.5) | <0.001 |
| SABA/SAMA | 0 (0.02) | 0 (0.02) | 0 (0.01) | <0.001 |
| **Major cardiac events in baseline 12m, n (%)** | | | | |
| New diagnosis for heart failure | 171 (2.6) | 1817 (2.6) | 1834 (1.1) | <0.001 |
| Hospitalisation for heart failure | 1700 (25.6) | 19810 (28.6) | 34738 (21.02) | <0.001 |
| Revascularization | 17 (0.3) | 64 (0.1) | 198 (0.1) | <0.01 |
| Myocardial Infarction | 72 (1.1) | 820 (1.2) | 1384 (0.8) | <0.001 |
| Stroke | 94 (1.4) | 981 (1.4) | 1560 (0.9) | <0.001 |
| Any of above | 1870 (28.2) | 21502 (31.0) | 37026 (22.4) | <0.001 |
| **Major cardiac events in follow-up 12m, n (%)** | | | | |
| New diagnosis for heart failure | 197 (3.0) | 1892 (2.7) | 1951 (1.2) | <0.001 |
| Hospitalisation for heart failure | 1677 (25.3) | 21429 (30.9) | 34257 (20.7) | <0.001 |
| Revascularization | 8 (0.1) | 51 (0.1) | 133 (0.1) | 0.42 |
| Myocardial Infarction | 83 (1.3) | 848 (1.2) | 1253 (0.8) | <0.001 |
| Stroke | 88 (1.3) | 1021 (1.5) | 1570 (1.0) | <0.001 |
| Any of above | 1866 (28.2) | 22948 (33.1) | 36545 (22.1) | <0.001 |
| **MRC dyspnoea score recorded in 12 months before index date, mean (SD)** | 2.2 (0.9) | 2.8 (1.1) | 2.2 (0.8) | <0.001 |
| No MRC score, n (%) | 3002 (45.3) | 19002 (27.4) | 157998 (95.6) | <0.001 |
| **MRC dyspnoea score recorded in 12 months before index date, n (%)** | | | | |
| 1-2 | 2501 (69.0) | 22693 (45.1) | 5402 (74.0) | <0.001 |
| 3-5 | 1126 (31.0) | 27578 (54.9) | 1896 (26.0) |  |
| **Spirometry values recorded in 12 months before index date, mean (SD)** | | | | |
| FEV_1_ % predicted | 64.2 (17.8) | 56.7 (19.5) | 85.2 (19.3) | <0.001 |
| FEV_1_; litres | 1.7 (0.6) | 1.4 (0.6) | 2.3 (0.7) | <0.001 |
| FVC; litres | 2.7 (0.9) | 2.5 (0.9) | 3.1 (0.9) | <0.001 |
| FEV_1_/FVC | 0.6 (0.1) | 0.6 (0.2) | 0.8 (0.1) | <0.001 |
| % No spirometry recorded | 2000 (30.2) | 24970 (36.1) | 158923 (96.1) | <0.001 |
| **COPD therapy in baseline 12m, n (%)** | | | | |
| No COPD therapy | 1373 (20.7) | 5067 (7.3) | 135139 (81.8) | <0.001 |
| Reliever only (SABA, SAMA and combinations) | 1840 (27.8) | 4981 (7.2) | 16022 (9.7) |  |
| ICS only (mono) | 704 (10.6) | 3333 (4.8) | 7108 (4.3) |  |
| LABA only (mono) | 126 (1.9) | 975 (1.4) | 136 (0.1) |  |
| LAMA only (mono) | 695 (10.5 | 4410 (6.4) | 459 (0.3) |  |
| LABA-ICS (Dual) | 81 (1.2) | 1189 (1.7) | 606 (0.4) |  |
| LABA-ICS fixed (Dual) | 989 (14.9) | 13285 (19.2) | 4851 (2.9) |  |
| LABA-LAMA (dual) | 29 (0.4) | 1013 (1.5) | 28 (0.02) |  |
| LABA-LAMA fixed (Dual) | 0 (0.0) | 0 (0.0) | 0 (0.0) |  |
| LAMA-ICS (dual) | 172 (2.6) | 1626 (2.4) | 118 (0.1) |  |
| LABA-LAMA-ICS (triple) | 607 (9.2) | 33195 (47.9) | 583 (0.4) |  |
| LABA-LAMA-ICS fixed (triple) | 0 (0.0) | 0 (0.0) | 0 (0.0) |  |
| **Clinically diagnosed comorbidities (ever), n (%)** | | | | |
| **Steroid related** | | | | |
| Diabetes type 2 | 892 (13.5) | 10587 (15.3) | 23300 (14.1) | <0.001 |
| Osteoporosis | 376 (5.7) | 6733 (9.7) | 5936 (3.6) | <0.001 |
| Hypertension | 2722 (41.1) | 30570 (44.1) | 54902 (33.2) | <0.001 |
| Chronic kidney disease | 916 (13.8) | 11165 (16.1) | 17129 (10.4) | <0.001 |
| Depression/Anxiety | 2490 (37.6) | 28908 (41.7) | 71490 (43.3) | <0.001 |
| Obesity | 2353 (35.5) | 26574 (38.4) | 64496 (39.02) | <0.001 |
| **Other** | | | | |
| CVD (ischaemic or coronary) | 1423 (21.5) | 17998 (26.0) | 24319 (14.7) | <0.001 |
| Prior asthma | 855 (12.9) | 28104 (40.6) | 17622 (10.7) | <0.001 |
| OSA | 94 (1.4) | 1493 (2.2) | 2624 (1.6) | <0.001 |
| GERD | 1014 (15.3) | 13717 (19.8) | 26184 (15.8) | <0.001 |
| Lung cancer | 57 (0.9) | 764 (1.1) | 480 (0.3) | <0.001 |
| Anaemia | 346 (5.2) | 4919 (7.1) | 9101 (5.5) | <0.001 |
| **Cambridge multimorbidity score, mean (SD)** | 2.6 (1.2) | 2.9 (1.4) | 0.9 (1.1) | <0.001 |
| **Hospital admission for any condition, mean (SD)** | | | | |
| In baseline 12m | 0.4 (0.9) | 0.5 (1.01) | 0.3 (0.8) | <0.001 |
| In follow-up 12m | 0.4 (0.9) | 0.6 (1.1) | 0.3 (0.8) | <0.001 |

BEC: blood eosinophil count; BMI: body mass index; COPD: chronic obstructive pulmonary disease; CVD: cardiovascular disease; FEV_1_: forced expiratory volume in one second; FVC: forced vital capacity; GERD: gastroesophageal reflux disease; ICS: inhaled corticosteroid; LABA: long-acting β_2_-agonist; LAMA: long-acting muscarinic antagonist; MRC: Medical Research Council; OCS: oral corticosteroid; OSA: obstructive sleep apnoea; SABA: short-acting β_2_-agonist; SAMA: short-acting muscarinic antagonist; SD: standard deviation

The baseline/follow up periods refer to 12 months pre/post index date. The 5-year periods were generated by aggregating all the records in each individual year and treating them as separate observations. Patients in individual years (2010-2014) were added together into one combined data set covering the whole 5-year period. Patients could be present in multiple years during that period and contribute multiple records to the aggregated analysis. Each patient index date combination was treated as an independent observation.

**S-Table 7: Patient characteristics (2015-2019)**

|  | **Newly diagnosed** | **Already diagnosed** | **Undiagnosed** | **p value** |
| --- | --- | --- | --- | --- |
| **N** | 6930 | 87237 | 159716 |  |
| **Age by index date, mean (SD)** | 68.2 (11.4) | 71.8 (10.5) | 61.9 (13.6) | <0.001 |
| **Age by index date, n (%)** | | | | |
| 40 - 49 years | 415 (6.0) | 1984 (2.3) | 35778 (22.4) | <0.001 |
| 50 - 59 years | 1190 (17.2) | 9523 (10.9) | 40050 (25.1) |  |
| 60 - 69 years | 2040 (29.4) | 22899 (26.3) | 35493 (22.2) |  |
| 70+ years | 3285 (47.4) | 52831 (60.6) | 48395 (30.3) |  |
| **Female, n (%)** | 3351 (48.4) | 44262 (50.7) | 89483 (56.0) | <0.001 |
| **Ethnicity, n (%)** | | | | |
| White | 4849 (70.0) | 63113 (72.4) | 107797 (67.5) | <0.001 |
| Mixed/ Multiple ethnic groups | 18 (0.3) | 167 (0.2) | 733 (0.5) |  |
| Asian / Asian British | 152 (2.2) | 1247 (1.4) | 5653 (3.5) |  |
| Black/ African/ Caribbean/ Black British | 17 (0.3) | 161 (0.2) | 762 (0.5) |  |
| Other ethnic group | 316 (4.6) | 4143 (4.8) | 7043 (4.4) |  |
| Missing ethnicity | 1578 (22.8) | 18406 (21.1) | 37728 (23.6) |  |
| **Smoking, n (%)** | | | | |
| Never-smoker | 324 (4.7) | 3610 (4.1) | 0.0 (0.0) | <0.001 |
| Current smoker | 3278 (47.3) | 30858 (35.4) | 66236 (41.5) |  |
| Former smoker | 3301 (47.6) | 52515 (60.2) | 93480 (58.5) |  |
| Missing smoking status | 27 (0.4) | 254 (0.3) | 0.0 (0.0) |  |
| **BMI (within 5 years of index date), n (%)** | | | | |
| Underweight (<18.5) | 263 (3.8) | 4674 (5.4) | 2757 (1.7) | <0.001 |
| Normal weight (18.5-24) | 2073 (29.9) | 27557 (31.6) | 37308 (23.4) |  |
| Overweight (25-29) | 2191 (31.6) | 26820 (30.7) | 49128 (30.8) |  |
| Obese (30.0+) | 2188 (31.6) | 26155 (30.0) | 54529 (34.1) |  |
| Missing BMI | 215 (3.1) | 2031 (2.3) | 15994 (10.0) |  |
| **BEC within 5 years of index date, mean (SD) count 10^9/L** | 0.2 (0.2) | 0.2 (0.2) | 0.2 (0.2) | <0.001 |
| **BEC within 5 years of index date - highest recorded; n (%)** | | | | |
| No BEC recorded in period | 271 (3.9) | 2705 (3.1) | 9265 (5.8) | <0.001 |
| <0.15 (10^9/L) | 2272 (32.8) | 30811 (35.3) | 57263 (35.9) |  |
| 0.15 <0.30 (10^9/L) | 2299 (33.2) | 28911 (33.1) | 53976 (33.8) |  |
| 0.30 <0.45 (10^9/L) | 1382 (19.9) | 17087 (19.6) | 28559 (17.9) |  |
| ≥ 0.45 (10^9/L) | 706 (10.2) | 7723 (8.9) | 10653 (6.7) |  |
| **Number of moderate exacerbations in baseline 12m, Mean (SD)** | 2.7 (2.0) | 3.7 (2.8) | 2.7 (2.3) | <0.001 |
| OCS prescriptions | 0.3 (1.0) | 0.5 (1.4) | 0.3 (1.4) | <0.001 |
| Antibiotic prescriptions | 1.9 (1.7) | 1.9 (2.3) | 2.3 (2.1) | <0.001 |
| OCS and Antibiotic prescriptions | 0.5 (0.9) | 1.4 (1.8) | 0.1 (0.4) | <0.001 |
| **Moderate exacerbations in baseline 12m, n (%)** | | | | |
| 0 | 560 (8.1) | 4279 (4.9) | 11060 (6.9) | <0.001 |
| 1 | 695 (10.0) | 7556 (8.7) | 19341 (12.1) |  |
| 2 | 2787 (40.2) | 26912 (30.9) | 76406 (47.8) |  |
| 3 | 1437 (20.7) | 16213 (18.6) | 23643 (14.8) |  |
| 4+ | 1451 (20.9) | 32277 (37) | 29266 (18.3) |  |
| **Number of severe exacerbations (hospital admittance for respiratory reason) in baseline 12m, mean (SD)** | 0.2 (0.5) | 0.2 (0.4) | 0.1 (0.3) | <0.001 |
| **Severe exacerbations in baseline 12m, n (%)** | | | | |
| 0 | 5569 (80.4) | 76129 (87.3) | 142388 (89.2) | <0.001 |
| 1 | 1252 (18.1) | 9778 (11.2) | 16469 (10.3) |  |
| 2+ | 109 (1.6) | 1330 (1.5) | 859 (0.5) |  |
| **Number of moderate exacerbations in follow-up 12m, mean (SD) [removed duplicates in same 7-day period]** | 2.0 (2.4) | 3.2 (3.2) | 1.7 (2.7) | <0.001 |
| Oral corticosteroid prescriptions | 0.3 (1.1) | 0.4 (1.4) | 0.3 (1.4) | <0.001 |
| Antibiotic prescriptions | 1.1 (1.8) | 1.6 (2.4) | 1.4 (2.3) | <0.001 |
| OCS and Antibiotic prescriptions | 0.6 (1.2) | 1.2 (1.8) | 0.1 (0.4) | <0.001 |
| **Moderate exacerbations in follow-up 12m, n (%)** | | | | |
| 0 | 2186 (31.5) | 16181 (18.6) | 72172 (45.2) | <0.001 |
| 1 | 1759 (25.4) | 16637 (19.1) | 36643 (22.9) |  |
| 2 | 1097 (15.8) | 14083 (16.1) | 18019 (11.3) |  |
| 3 | 644 (9.3) | 10649 (12.2) | 9595 (6.0) |  |
| 4+ | 1244 (18.0) | 29687 (34.0) | 23287 (14.6) |  |
| **Number of severe exacerbations (hospital admittance for respiratory reason) in follow-up 12m, mean (SD)** | 0.1 (0.3) | 0.1 (0.4) | 0.04 (0.2) | <0.001 |
| **Severe exacerbations in follow-up 12m, n (%)** | | | | |
| 0 | 6374 (92.0) | 78719 (90.2) | 154740 (96.9) | <0.001 |
| 1 | 481 (6.9) | 7072 (8.1) | 4429 (2.8) |  |
| 2+ | 75 (1.1) | 1446 (1.7) | 547 (0.3) |  |
| **Number of rescue inhaler prescriptions in baseline 12m, mean (SD)** | 2.5 (3.6) | 6.5 (5.9) | 0.6 (2.0) | <0.001 |
| SABA | 2.4 (3.4) | 6.2 (5.4) | 0.6 (2.0) | <0.001 |
| SAMA | 0.1 (0.8) | 0.4 (1.8) | 0.01 (0.4) | <0.001 |
| SABA/SAMA | 0.0 (0.0) | 0.0 (0.0) | 0.0 (0.0) | <0.001 |
| **Number of rescue inhaler prescriptions in follow-up 12m, mean (SD)** | 4.3 (4.8) | 6.4 (5.8) | 0.6 (2.2) | <0.001 |
| SABA | 4.1 (4.6) | 6.1 (5.3) | 0.6 (2.1) | <0.001 |
| SAMA | 0.1 (1.03) | 0.3 (1.8) | 0.01 (0.4) | <0.001 |
| SABA/SAMA | 0.0 (0.0) | 0.0 (0.0) | 0.0 (0.0) | <0.001 |
| **Major cardiac events in baseline 12m, n (%)** | | | | |
| New diagnosis for heart failure | 249 (3.6) | 2583 (3.0) | 2575 (1.6) | <0.001 |
| Hospitalisation for heart failure | 2678 (38.6) | 33863 (38.8) | 53963 (33.8) | <0.001 |
| Revascularization | 9 (0.1) | 44 (0.1) | 136 (0.1) | <0.01 |
| Myocardial Infarction | 84 (1.2) | 944 (1.1) | 1557 (1.0) | <0.05 |
| Stroke | 103 (1.5) | 1268 (1.5) | 1823 (1.1) | <0.001 |
| Any of above | 2783 (40.2) | 35147 (40.3) | 55463 (34.7) | <0.001 |
| **Major cardiac events in follow-up 12m, n (%)** | | | |  |
| New diagnosis for heart failure | 267 (3.9) | 2935 (3.4) | 2650 (1.7) | <0.001 |
| Hospitalisation for heart failure | 2508 (36.2) | 34091 (39.1) | 46181 (28.9) | <0.001 |
| Revascularization | <5 | 37 (0.04) | 85 (0.1) | 0.49 |
| Myocardial Infarction | 68 (1.0) | 985 (1.1) | 1164 (0.7) | <0.001 |
| Stroke | 97 (1.4) | 1318 (1.5) | 1620 (1.01) | <0.001 |
| Any of above | 2640 (38.1) | 35535 (40.7) | 48004 (30.1) | <0.001 |
| **MRC dyspnoea score recorded in 12 months before index date, mean (SD)** | 2.3 (0.9) | 2.8 (1.1) | 2.2 (0.9) | <0.001 |
| No MRC score, N (%) | 3608 (52.1) | 20397 (23.4) | 151127 (94.6) | <0.001 |
| **MRC dyspnoea score recorded in 12 months before index date, n (%) [If multiple values present, closest value to index date used].** | | | | |
| **1-2** | 2156 (64.9) | 29085 (43.5) | 6109 (71.1) | <0.001 |
| **3-5** | 1166 (35.1) | 37755 (56.5) | 2480 (28.9) |  |
| **Spirometry values recorded in 12 months before index date, mean (SD)** | | | | |
| FEV_1_ % predicted | 66.9 (18.8) | 59.3 (20.2) | 85.5 (19.3) | <0.001 |
| FEV_1_; litres | 1.8 (0.7) | 1.5 (0.6) | 2.3 (0.8) | <0.001 |
| FVC; litres | 2.8 (0.9) | 2.5 (0.9) | 3.1 (0.9) | <0.001 |
| FEV_1_/FVC | 0.6 (0.1) | 0.6 (0.2) | 0.8 (0.1) | <0.001 |
| % No spirometry recorded | 2463 (35.5) | 30491 (35.0) | 153369 (96.0) | <0.001 |
| **COPD therapy in baseline 12m, N (%)** | | | | |
| No COPD therapy | 1382 (19.9) | 5930 (6.8) | 129316 (81.0) | <0.001 |
| Reliever only (SABA, SAMA and combinations) | 1742 (25.1) | 5048 (5.8) | 16362 (10.2) |  |
| ICS only (mono) | 536 (7.7) | 1803 (2.1) | 6008 (3.8) |  |
| LABA only (mono) | 130 (1.9) | 1102 (1.3) | 122 (0.1) |  |
| LAMA only (mono) | 1026 (14.8) | 7145 (8.2) | 734 (0.5) |  |
| LABA-ICS (Dual) | 31 (0.5) | 475 (0.5) | 277 (0.2) |  |
| LABA-ICS fixed (Dual) | 910 (13.1) | 12737 (14.6) | 5349 (3.4) |  |
| LABA-LAMA (dual) | 23 (0.3) | 1282 (1.5) | 20 (0.01) |  |
| LABA-LAMA fixed (Dual) | 247 (3.6) | 3711 (4.3) | 132 (0.1) |  |
| LAMA-ICS (dual) | 200 (2.9) | 1047 (1.2) | 125 (0.1) |  |
| LABA-LAMA-ICS (triple) | 678 (9.8) | 44866 (51.4) | 872 (0.6) |  |
| LABA-LAMA-ICS fixed (triple) | 17 (0.3) | 1963 (2.3) | 23 (0.01) |  |
| **Clinically diagnosed comorbidities (ever), n(%)** | | | | |
| **Steroid related** | | | | |
| Diabetes type 2 | 1089 (15.7) | 16399 (18.8) | 26489 (16.6) | <0.001 |
| Osteoporosis | 461 (6.7) | 9347 (10.7) | 7361 (4.6) | <0.001 |
| Hypertension | 2932 (42.3) | 40849 (46.8) | 57755 (36.2) | <0.001 |
| Chronic kidney disease | 971 (14.01) | 14288 (16.4) | 17970 (11.3) | <0.001 |
| Depression/Anxiety | 2858 (41.2) | 40659 (46.6) | 76771 (48.1) | <0.001 |
| Obesity | 2833 (40.9) | 37834 (43.4) | 70725 (44.3) | <0.001 |
| **Other** | | | | |
| CVD (ischaemic or coronary) | 1431 (20.7) | 21520 (24.7) | 24104 (15.1) | <0.001 |
| Prior asthma | 760 (11.0) | 31088 (35.6) | 18032 (11.3) | <0.001 |
| OSA | 162 (2.3) | 2811 (3.2) | 4074 (2.6) | <0.001 |
| GERD | 1239 (17.9) | 19879 (22.8) | 30231 (18.9) | <0.001 |
| Lung cancer | 79 (1.1) | 1318 (1.5) | 699 (0.4) | <0.001 |
| Anaemia | 438 (6.3) | 7555 (8.7) | 11681 (7.3) | <0.001 |
| **Cambridge multimorbidity score, mean (SD)** | 2.7 (1.3) | 3.0 (1.4) | 1.08 (1.2) | <0.001 |
| **Hospital admission for any condition, mean (SD)** | | | | |
| In baseline 12m | 0.7 (1.1) | 0.7 (1.2) | 0.56 (1.04) | <0.001 |
| In follow-up 12m | 0.6 (1.1) | 0.7 (1.3) | 0.48 (1.0) | <0.001 |

BEC: blood eosinophil count; BMI: body mass index; COPD: chronic obstructive pulmonary disease; CVD: cardiovascular disease; FEV_1_: forced expiratory volume in one second; FVC: forced vital capacity; GERD: gastroesophageal reflux disease; ICS: inhaled corticosteroid; LABA: long-acting β_2_-agonist; LAMA: long-acting muscarinic antagonist; MRC: Medical Research Council; OCS: oral corticosteroid; OSA: obstructive sleep apnoea; SABA: short-acting β_2_-agonist; SAMA: short-acting muscarinic antagonist; SD: standard deviation

The baseline/follow up periods refer to 12 months pre/post index date. The 5-year periods were generated by aggregating all the records in each individual year and treating them as separate observations. Patients in individual years (2015-2019) were added together into one combined data set covering the whole 5-year period. Patients could be present in multiple years during that period and contribute multiple records to the aggregated analysis. Each patient index date combination was treated as an independent observation.

**S-Figure 5 Spirometry status by year in the already diagnosed cohort.**


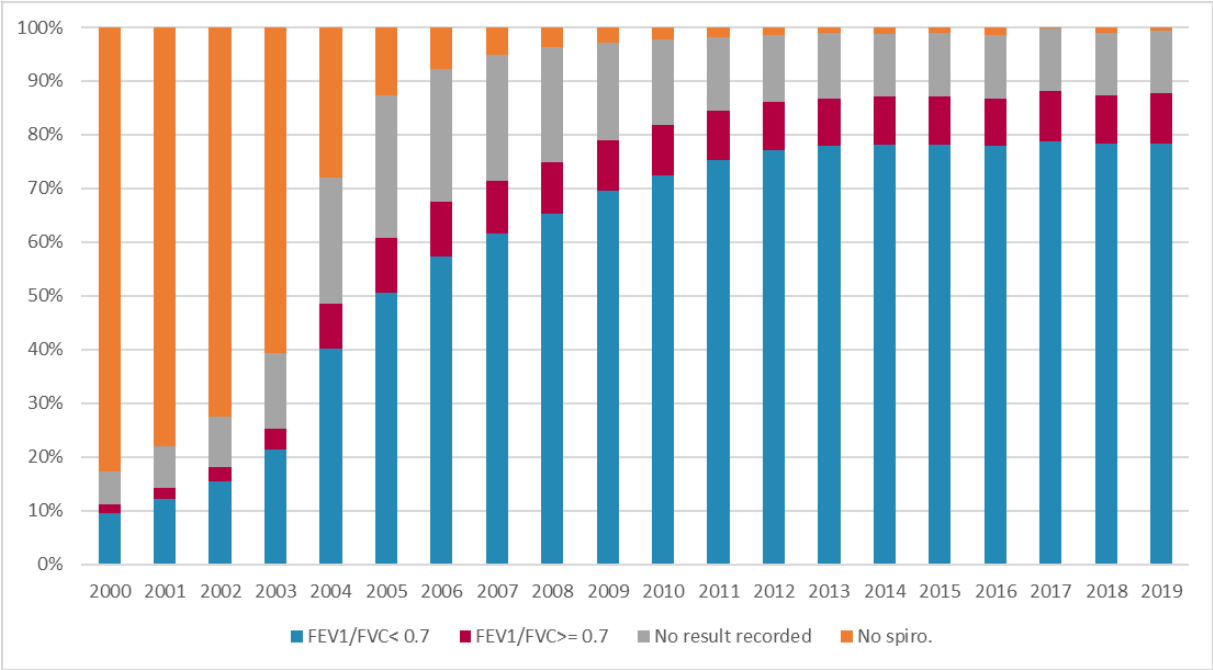


FEV_1_: forced expiratory volume in one second; FVC: forced vital capacity; no spiro.: no spirometry

S-Table 8: Percentage of high-risk **newly diagnosed patients with COPD** who met relevant CONQUEST quality standards from 2000-2019

| Year | 2000 | 2001 | 2002 | 2003 | 2004 | 2005 | 2006 | 2007 | 2008 | 2009 | 2010 | 2011 | 2012 | 2013 | 2014 | 2015 | 2016 | 2017 | 2018 | 2019 |
| --- | --- | --- | --- | --- | --- | --- | --- | --- | --- | --- | --- | --- | --- | --- | --- | --- | --- | --- | --- | --- |
| N | 664 | 780 | 776 | 865 | 902 | 1071 | 1026 | 1011 | 1079 | 1140 | 1165 | 1241 | 1409 | 1486 | 1328 | 1384 | 1384 | 1371 | 1448 | 1343 |
| **Spirometry*** | 10.8 | 11.8 | 12.2 | 18.6 | 28.6 | 51.3 | 63.9 | 64.6 | 65.5 | 67.0 | 66.4 | 67.2 | 71.6 | 70.9 | 72.2 | 69.7 | 67.2 | 62.9 | 63.3 | 59.0 |
| **Spirometry†** | 17.3 | 17.1 | 20.0 | 29.6 | 55.9 | 81.5 | 85.8 | 87.3 | 86.5 | 87.5 | 86.3 | 88.1 | 89.3 | 88.5 | 88.4 | 88.1 | 86.8 | 85.5 | 84.9 | 80.9 |
| **Exacerbation history†** | 0.0 | 0.0 | 0.0 | 0.0 | 0.1 | 0.0 | 1.3 | 2.6 | 3.7 | 6.3 | 12.1 | 20.1 | 24.7 | 23.7 | 28.3 | 31.0 | 31.4 | 34.8 | 37.5 | 41.8 |
| **Cardiac risk†§** | 1.4 | 3.5 | 4.5 | 8.0 | 8.0 | 8.9 | 8.5 | 10.0 | 8.6 | 11.8 | 18.5 | 19.5 | 23.5 | 21.1 | 23.2 | 24.1 | 22.3 | 23.1 | 19.8 | 19.1 |
| **CAT†** | 0.2 | 0.0 | 0.0 | 0.0 | 0.0 | 0.0 | 0.1 | 0.2 | 0.0 | 0.0 | 0.2 | 2.9 | 10.0 | 13.6 | 16.0 | 12.4 | 15.7 | 17.8 | 22.4 | 25.5 |
| **mMRC†** | 18.7 | 19.7 | 19.3 | 21.2 | 28.6 | 40.1 | 42.3 | 47.2 | 49.6 | 56.7 | 84.5 | 86.9 | 88.3 | 88.3 | 88.3 | 86.4 | 85.3 | 86.2 | 85.7 | 83.4 |
| **Smoking Status*** | 25.9 | 30.1 | 36.2 | 43.0 | 53.9 | 79.5 | 87.7 | 86.3 | 89.8 | 88.8 | 89.2 | 88.2 | 89.2 | 89.4 | 90.1 | 90.1 | 87.8 | 86.5 | 83.9 | 85.4 |
| **Treatment***  None  Reliever only  Theophylline  ICS  LABA  LAMA  LABA/ICS  LABA/LAMA  LAMA/ICS  LABA/LAMA/ICS | 22.0  21.8  1.2  43.4  1.2  0.0  10.4  0.0  0.0  0.0 | 22.6  18.8  0.9  41.9  1.9  0.0  13.7  0.0  0.0  0.0 | 21.9  22.0  1.0  38.8  1.5  0.0  14.7  0.0  0.0  0.0 | 24.5  21.0  1.5  32.4  2.8  0.2  17.4  0.0  0.1  0.1 | 21.8  25.4  0.8  28.7  1.8  1.1  18.1  0.1  0.8  1.3 | 25.3  27.1  0.3  22.8  2.8  0.8  17.3  0.0  0.7  2.6 | 24.4  26.5  0.4  19.2  1.9  2.0  20.9  0.4  0.8  3.4 | 22.4  30.6  0.4  19.0  1.9  2.7  17.7  0.3  1.0  4.1 | 24.1  28.5  0.1  18.4  1.2  4.0  16.2  0.2  2.3  4.7 | 23.6  28.7  0.4  13.7  1.5  5.7  17.8  0.4  1.9  6.3 | 21.8  28.8  0.2  13.0  1.4  7.8  18.0  0.4  2.1  6.4 | 20.3  27.7  0.2  12.6  1.3  8.9  16.5  0.2  2.9  9.3 | 19.4  28.3  0.1  11.1  1.7  10.3  16.3  0.2  2.6  9.9 | 21.1  27.2  0.0  9.6  2.4  12.7  14.0  0.8  2.6  9.6 | 21.0  27.0  0.0  7.4  2.6  12.1  16.4  0.5  2.8  10.2 | 19.5  24.6  0.0  9.8  2.5  15.0  15.9  0.5  3.0  9.0 | 18.7  26.8  0.1  9.2  2.6  14.7  15.3  0.8  2.6  9.2 | 22.2  24.7  0.0  7.1  1.8  14.3  12.4  3.9  2.9  10.0 | 18.4  24.2  0.1  6.2  1.3  15.8  12.5  6.6  3.2  11.3 | 21.0  25.4  0.0  6.3  1.1  14.1  11.6  6.4  2.8  10.7 |
| **Median time to start of new therapy post exacerbation (days)** | 1975 | 1729 | 1608 | 1405 | 1249 | 1056 | 721 | 702 | 557 | 381 | 314 | 356 | 224 | 316 | 368 | 427 | 463 | 287 | 489 | 584 |
| **N** | 184 | 191 | 232 | 281 | 354 | 436 | 459 | 477 | 535 | 619 | 653 | 688 | 835 | 872 | 734 | 809 | 770 | 804 | 849 | 783 |
| **Medication review‡** | 2.7 | 4.2 | 6.9 | 13.2 | 37.9 | 59.2 | 61.9 | 67.7 | 63.2 | 64.1 | 65.7 | 65.0 | 66.3 | 60.9 | 61.0 | 55.0 | 59.1 | 59.1 | 53.4 | 55.0 |
| **N** | 44 | 55 | 50 | 65 | 102 | 179 | 175 | 178 | 211 | 277 | 395 | 413 | 461 | 477 | 452 | 501 | 499 | 472 | 534 | 498 |
| **Pulmonary rehabilitation¶** | 0.0 | 0.0 | 0.0 | 0.0 | 2.0 | 1.7 | 1.7 | 5.6 | 1.9 | 4.3 | 5.8 | 6.3 | 11.1 | 18.0 | 19.9 | 22.4 | 23.8 | 27.8 | 25.3 | 33.7 |

* assessed in the 12-month period before Jan 1^st^; †12-month either side of COPD diagnosis; ‡ COPD medication review within 6 months of treatment change; ¶patients with mMRC ≥2 either side of 1^st^ January each year offered or referred for pulmonary rehabilitation within 12 months of mMRC score; § Pre-2007 we searched for any coding of cardiac risk, including Framingham score, Joint British Societies cardiac risk as well as additional evidence of cardiac risk assessments. The data were dominated by QRISK post 2007.

CAT: COPD Assessment Test; CONQUEST: The COllaboratioN on QUality improvement initiative for achieving Excellence in STandards of COPD care; COPD: chronic obstructive pulmonary disease; ICS: inhaled corticosteroid; LABA: long-acting β2-agonist; LAMA: long-acting muscarinic antagonist; mMRC: modified Medical Research Council

S-Table 9: Percentage of high-risk **already diagnosed patients with COPD** who met relevant CONQUEST quality standards from 2000-2019

| Year | 2000 | 2001 | 2002 | 2003 | 2004 | 2005 | 2006 | 2007 | 2008 | 2009 | 2010 | 2011 | 2012 | 2013 | 2014 | 2015 | 2016 | 2017 | 2018 | 2019 |
| --- | --- | --- | --- | --- | --- | --- | --- | --- | --- | --- | --- | --- | --- | --- | --- | --- | --- | --- | --- | --- |
| N | 4585 | 5297 | 6322 | 7097 | 7677 | 7064 | 8156 | 9045 | 10084 | 11085 | 11693 | 12902 | 13854 | 15111 | 15713 | 16607 | 17049 | 17757 | 17966 | 17858 |
| **Spirometry*** | 5.1 | 5.2 | 5.9 | 8.3 | 15.5 | 43.5 | 45.4 | 58.1 | 60.3 | 62.1 | 62.9 | 63.3 | 64.0 | 63.6 | 65.5 | 64.4 | 66.2 | 65.7 | 64.6 | 64.3 |
| **Spirometry†** | 9.1 | 9.0 | 11.8 | 20.3 | 51.0 | 67.8 | 72.0 | 79.3 | 79.3 | 80.1 | 81.2 | 81.2 | 81.8 | 81.1 | 80.6 | 80.9 | 81.3 | 81.0 | 81.2 | 78.9 |
| **Exacerbation history†** | 0.0 | 0.0 | 0.1 | 0.0 | 0.1 | 0.3 | 2.0 | 4.6 | 6.9 | 13.7 | 23.6 | 32.8 | 38.4 | 40.8 | 42.3 | 46.3 | 49.7 | 52.1 | 57.3 | 61.4 |
| **Cardiac risk†§** | 1.6 | 3.6 | 6.0 | 7.8 | 8.0 | 7.4 | 7.4 | 7.8 | 7.2 | 14.3 | 18.7 | 17.5 | 17.9 | 19.5 | 19.4 | 19.5 | 19.6 | 18.0 | 17.9 | 18.2 |
| **mMRC†** | 15.9 | 16.0 | 18.2 | 21.3 | 27.8 | 35.2 | 39.3 | 46.2 | 51.2 | 74.7 | 87.5 | 88.7 | 89.2 | 89.2 | 89.1 | 89.4 | 90.4 | 90.4 | 91.3 | 90.9 |
| **Smoking status*** | 19.2 | 23.3 | 28.3 | 32.4 | 48.8 | 82.5 | 82.7 | 85.5 | 86.3 | 86.3 | 84.1 | 82.6 | 83.4 | 83.9 | 85.5 | 84.5 | 85.6 | 84.7 | 84.3 | 85.1 |
| **Pn vaccination‡** | 40.5 | 39.5 | 41.0 | 42.2 | 43.4 | 44.3 | 50.2 | 51.0 | 51.4 | 51.9 | 51.8 | 50.7 | 50.8 | 51.4 | 51.6 | 51.5 | 51.8 | 51.6 | 52.0 | 51.4 |
| **Flu vaccination†** | 50.9 | 53.5 | 54.3 | 52.9 | 55.2 | 55.8 | 57.2 | 55.3 | 55.1 | 57.5 | 59.2 | 57.6 | 59.5 | 60.6 | 62.7 | 66.9 | 72.1 | 75.2 | 72.8 | 70.7 |
| **Treatment***  None  Reliever only  Theophylline  ICS  LABA  LAMA  LABA/ICS  LABA/LAMA  LAMA/ICS  LABA/LAMA/ICS | 16.8  12.6  2.6  48.7  2.1  0.0  17.1  0.0  0.0  0.0 | 15.1  12.6  2.1  44.6  3.1  0.0  22.4  0.0  0.0  0.0 | 14.2  13.4  1.9  39.2  3.7  0.0  27.4  0.0  0.0  0.0 | 11.9  12.8  1.7  36.5  3.8  0.2  31.6  0.2  0.4  0.9 | 12.1  12.8  1.4  28.1  3.9  0.9  32.4  0.5  1.8  6.1 | 12.0  11.5  1.1  21.4  3.3  1.5  34.0  0.8  2.2  12.0 | 10.6  10.9  0.7  17.0  3.1  1.5  34.6  0.8  2.6  18.0 | 9.7  10.7  0.7  13.1  2.4  2.1  34.4 0.9  2.3  23.6 | 9.8  9.6  0.6  10.7  1.9  2.8  32.5  1.0  2.3  28.7 | 8.1  9.0  0.3  9.2  1.7  3.9  28.4  1.2  2.7  35.3 | 8.0  8.5  0.3  7.6  1.7  4.5  25.4  1.2  2.6  40.1 | 7.5  7.7  0.2  5.8  1.3  5.5  22.5  1.3  2.8  45.2 | 7.2  7.1  0.2  4.6  1.2  6.2  20.3  1.5  2.4  49.3 | 7.1  6.6  0.2  3.6  1.3  7.2  19.9  1.6  2.2  50.2 | 6.9  6.5  0.1  3.2  1.5  7.8  17.8  1.7  1.9  52.5 | 7.1  6.3  0.1  2.9  1.5  7.7  17.8  2.0  1.8  52.9 | 7.1  5.7  0.0  2.3  1.5  8.1  16.5  3.3  1.3  53.9 | 6.7  5.7  0.1  2.1  1.3  8.2  15.2  4.9  1.2  54.6 | 6.4  5.5  0.1  1.8  1.0  8.4  13.8  7.9  1.0  53.9 | 6.6  5.7  0.0  1.3  1.1  8.5  12.7  10.2  0.8  53.1 |
| **Median time to start of new therapy post exacerbation (days)** | 1036 | 870 | 778 | 603 | 481 | 467 | 500 | 501 | 503 | 481 | 404 | 395 | 414 | 442 | 418 | 404 | 342 | 302 | 231 | 148 |
| **N** | 232 | 270 | 358 | 508 | 927 | 1263 | 1752 | 2304 | 2797 | 4911 | 6464 | 7265 | 7849 | 8491 | 8916 | 9547 | 10048 | 10557 | 10811 | 10725 |
| **Pulmonary rehabilitation¶** | 0.0 | 0.0 | 0.0 | 0.4 | 2.6 | 3.1 | 4.2 | 4.2 | 5.4 | 7.2 | 10.1 | 14. 2 | 19.8 | 26.5 | 29.6 | 28.1 | 30.7 | 31.8 | 34.0 | 53.9 |
| **N** | 843 | 1044 | 1327 | 1656 | 1893 | 1759 | 2172 | 2337 | 2597 | 3050 | 3291 | 4060 | 4549 | 4880 | 5598 | 6307 | 6549 | 6848 | 7167 | 7361 |
| **COPD review§** | 15.3 | 16.4 | 21.3 | 31.0 | 54.3 | 62.3 | 64.0 | 63.7 | 63.3 | 65.2 | 67.2 | 66.9 | 65.1 | 64.8 | 62.4 | 61.2 | 59.6 | 59.9 | 58.6 | 58.9 |

* assessed in the 12-month period before Jan 1^st^; †: in the 12 months before or after 1 January; ‡ Before 1 January; ¶patients with mMRC ≥2 before or after 1 January each year offered or referred for pulmonary rehabilitation within 12 months of mMRC score; §within 6 weeks of respiratory hospitalization;
§ Pre-2007 we searched for any coding of cardiac risk, including Framingham score, Joint British Societies cardiac risk as well as additional evidence of cardiac risk assessments. The data were dominated by QRISK post 2007.

CONQUEST: The COllaboratioN on QUality improvement initiative for achieving Excellence in STandards of COPD care; COPD: chronic obstructive pulmonary disease; Flu: influenzae; ICS: inhaled corticosteroid; LABA: long-acting β2-agonist; LAMA: long-acting muscarinic antagonist; mMRC: modified Medical Research Council; PN: pneumococcal

S-Table 10: Percentage of high-risk **undiagnosed patients with COPD** who met relevant CONQUEST quality standards from 2000-2019

| Year | 2000 | 2001 | 2002 | 2003 | 2004 | 2005 | 2006 | 2007 | 2008 | 2009 | 2010 | 2011 | 2012 | 2013 | 2014 | 2015 | 2016 | 2017 | 2018 | 2019 |
| --- | --- | --- | --- | --- | --- | --- | --- | --- | --- | --- | --- | --- | --- | --- | --- | --- | --- | --- | --- | --- |
| N | 16253 | 18073 | 21997 | 23864 | 25644 | 25165 | 26068 | 27246 | 30172 | 30931 | 31105 | 32809 | 32833 | 35060 | 33489 | 33510 | 31795 | 31883 | 31323 | 31205 |
| **New diagnosis*** | 2.4 | 2.2 | 1.9 | 2.3 | 2.6 | 2.4 | 2.0 | 2.3 | 1.9 | 2.0 | 2.1 | 2.4 | 2.4 | 2.1 | 2.1 | 2.2 | 2.4 | 2.4 | 2.3 | 2.4 |
| **Spirometry†** | 0.4 | 0.5 | 0.6 | 0.8 | 1.1 | 2.7 | 3.0 | 2.9 | 3.3 | 3.5 | 3.5 | 3.8 | 3.9 | 4.1 | 3.9 | 4.1 | 3.9 | 4.0 | 4.1 | 3.8 |
| **Spirometry¶** | 1.2 | 1.5 | 1.6 | 2.6 | 5.3 | 7.1 | 6.7 | 6.9 | 7.4 | 7.8 | 7.7 | 8.5 | 8.6 | 8.5 | 8.1 | 8.2 | 8.2 | 8.5 | 8.1 | 7.9 |
| **Smoking status†** | 21.8 | 26.6 | 31.9 | 34.3 | 45.6 | 67.8 | 63.1 | 69.1 | 72.7 | 71.4 | 70.9 | 67.6 | 67.3 | 68.6 | 69.9 | 65.0 | 62.4 | 61.3 | 60.3 | 60.5 |
| **Treatment†**  None  Reliever only  Theophylline  ICS  LABA  LAMA  LABA/ICS  LABA/LAMA  LAMA/ICS  LABA/LAMA/ICS | 79.0  7.3  0.2  10.5  0.2  0.0  2.7  0.0  0.0  0.0 | 79.4  7.0  0.3  10.3  0.3  0.0  2.7  0.0  0.0  0.0 | 79.0  7.7  0.3  9.7  0.3  0.0  2.9  0.0  0.0  0.0 | 78.7  8.0  0.2  9.4  0.3  0  3.3  0.0  0.0  0.0 | 79.1  8.7  0.2  8.1  0.3  0.1  3.5  0.0  0.0  0.1 | 83.0  8.1  0.1  5.8  0.2  0.0  2.6  0.0  0.0  0.1 | 83.9  8.2  0.1  4.9  0.2  0.1  2.5  0.0  0.0  0.1 | 84.1  8.1  0.1  4.8  0.1  0.1  2.5  0.0  0.0  0.1 | 83.5  8.3  0.1  4.9  0.1  0.1  2.7  0.0  0.0  0.2 | 82.3  9.1  0.1  4.9  0.1  0.1  3.0  0.0  0.1  0.3 | 82.6  8.8  0  4.5  0.1  0.2  3.3  0.0  0.1  0.3 | 81.2  10.1  0.0  4.6  0.1  0.2  3.2  0.0  0.1  0.3 | 81.9  9.3  0.0  4.4  0.1  0.3  3.4  0.0  0.1  0.4 | 81.4  10.1  0.0  4.2  0.1  0.3  3.4  0.0  0.1  0.3 | 81.8  10.1  0.0  3.9  0.1  0.3  3.1  0.0  0.1  0.4 | 80.7  10.4  0.0  4.1  0.1  0.4  3.6  0.0  0.1  0.5 | 81.1  10.2  0.0  3.8  0.1  0.4  3.5  0.0  0.1  0.6 | 80.5  10.7  0.0  3.9  0.1  0.4  3.5  0.1  0.1  0.5 | 81.4  9.8  0.0  3.6  0.1  0.6  3.5  0.1  0.1  0.6 | 81.2  10.0  0.0  3.4  0.1  0.6  3.5  0.2  0.1  0.6 |
| N | 2051 | 2087 | 2886 | 3570 | 4070 | 4038 | 4327 | 4644 | 5276 | 5889 | 5909 | 6558 | 7306 | 7782 | 8481 | 9041 | 9062 | 9121 | 9363 | 9649 |
| **COPD review‡** | 5.3 | 5.5 | 8.4 | 13.5 | 33.2 | 38.0 | 40.3 | 40.1 | 38.9 | 41.0 | 41.7 | 42.4 | 41.9 | 39.6 | 38.0 | 36.7 | 36.8 | 36.5 | 35.4 | 34.3 |
| N | 16253 | 18073 | 21997 | 23864 | 25644 | 25165 | 26068 | 27246 | 30172 | 30931 | 31105 | 32809 | 32833 | 35060 | 33489 | 33510 | 31795 | 31883 | 31323 | 31205 |
| **Cardiac risk¶§** | 2.0 | 4.2 | 6.9 | 9.0 | 9.7 | 8.6 | 8.7 | 8.7 | 9.0 | 17.0 | 22.2 | 20.6 | 20.9 | 22.7 | 21.9 | 22.2 | 21.0 | 20.9 | 21.0 | 20.4 |
| **mMRC¶** | 3.8 | 4.2 | 4.9 | 6.1 | 6.9 | 7.0 | 7.0 | 7.0 | 6.9 | 7.7 | 8.2 | 8.6 | 8.5 | 8.3 | 8.0 | 8.5 | 9.0 | 9.5 | 10.3 | 10.9 |

* in the 12-month period after 1 January; †in the 12-month period before 1 January; ‡within 6 weeks post respiratory hospitalization; ¶ within 12 months before or after 1 January of each year; § Pre-2007 we searched for any coding of cardiac risk, including Framingham score, Joint British Societies cardiac risk as well as additional evidence of cardiac risk assessments. The data were dominated by QRISK post 2007.

CONQUEST: The COllaboratioN on QUality improvement initiative for achieving Excellence in STandards of COPD care; COPD: chronic obstructive pulmonary disease; ICS: inhaled corticosteroid; LABA: long-acting β2-agonist; LAMA: long-acting muscarinic antagonist; mMRC: modified Medical Research Council

# Data based on specific definition of exacerbations requiring a respiratory-related code within 3 days of a steroid/antibiotic prescription

**S-Figure 6A: Median time to initiation of new therapy after an exacerbation (newly diagnosed cohort - based on specific definition of exacerbations)**


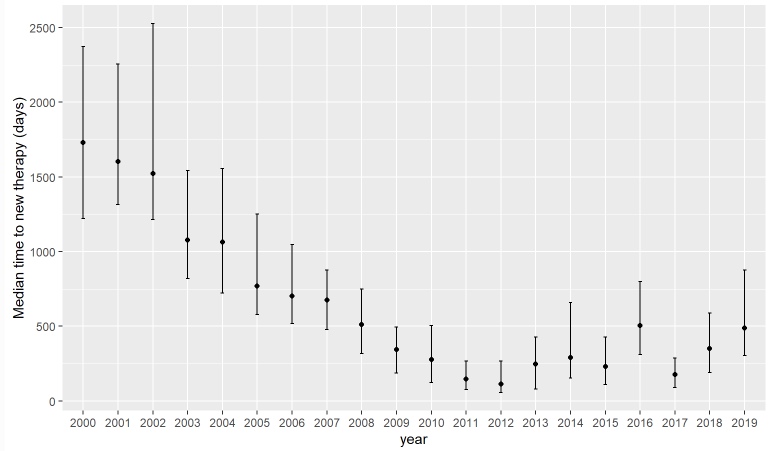
Data are presented as median and upper/lower 95% confidence interval**-**

**S-Figure 6B: Median time to initiation of new therapy after an exacerbation (already diagnosed cohort - based on specific definition of exacerbations)**


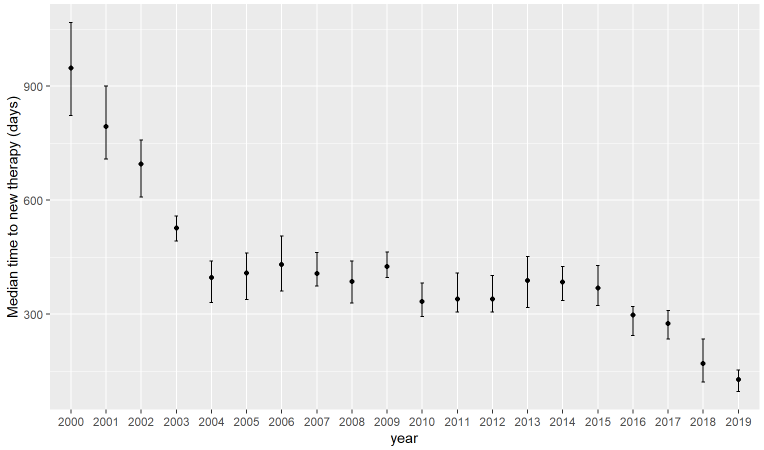


Data are presented as median and upper/lower 95% confidence interval

**S-Table 11 Eligible and high-risk patient numbers by year (based on specific definition of exacerbations)**

| **cohort** | | **2000** | | | **2001** | | **2002** | | **2003** | | **2004** | | **2005** | | **2006** | | **2007** | |  | | **2008** | | **2009** | | **2010** | | **2011** | | **2012** | | **2013** | | **2014** | | **2015** | | **2016** | | **2017** | | **2018** | **2019** |
| --- | --- | --- | --- | --- | --- | --- | --- | --- | --- | --- | --- | --- | --- | --- | --- | --- | --- | --- | --- | --- | --- | --- | --- | --- | --- | --- | --- | --- | --- | --- | --- | --- | --- | --- | --- | --- | --- | --- | --- | --- | --- | --- |
|  | | | **Eligible patients (primary care population)*** | | | | | | | | | | | | | | | | | | | | | | | | | | | | | | | | | | | | | | | |
| **ND** | 2326 | | | 2761 | | 2726 | | 2911 | | 2962 | | 3522 | | 3467 | | 3336 | |  | | 3310 | | 3319 | | 3497 | | 3719 | | 4074 | | 4178 | | 3959 | | 3975 | | 4136 | | 4105 | | 4239 | | 4136 |
| **AD** | 15747 | | | 17803 | | 19990 | | 21662 | | 22634 | | 22031 | | 25252 | | 27733 | |  | | 29680 | | 31601 | | 33438 | | 35120 | | 36807 | | 37827 | | 39965 | | 42280 | | 43731 | | 45184 | | 46918 | | 48063 |
| **PUD** | 212776 | | | 235207 | | 266449 | | 291691 | | 313723 | | 338730 | | 358353 | | 375381 | |  | | 393878 | | 410577 | | 425421 | | 435970 | | 445644 | | 451209 | | 459881 | | 468749 | | 476963 | | 484924 | | 496353 | | 506894 |
|  | | | **High risk patients (analysis sample)†** | | | | | | | | | | | | | | | | | | | | | | | | | | | | | | | | | | | | | | | |
| **ND** | 157 | | | 194 | | 205 | | 232 | | 277 | | 404 | | 414 | | 429 | |  | | 462 | | 502 | | 544 | | 537 | | 663 | | 676 | | 644 | | 685 | | 667 | | 735 | | 790 | | 782 |
| **%‡** | 6.7 | | | 7.0 | | 7.5 | | 8.0 | | 9.4 | | 11.5 | | 11.9 | | 12.9 | |  | | 14.0 | | 15.1 | | 15.6 | | 14.4 | | 16.3 | | 16.2 | | 16.3 | | 17.2 | | 16.1 | | 17.9 | | 18.6 | | 18.9 |
| **AD** | 1360 | | | 1594 | | 2147 | | 2770 | | 3265 | | 3434 | | 4005 | | 4591 | |  | | 5129 | | 5613 | | 6116 | | 6820 | | 7425 | | 7851 | | 8170 | | 8647 | | 9200 | | 9982 | | 10343 | | 10636 |
| **%‡** | 8.6 | | | 9.0 | | 10.7 | | 12.8 | | 14.4 | | 15.6 | | 15.9 | | 16.6 | |  | | 17.3 | | 17.8 | | 18.3 | | 19.4 | | 20.2 | | 20.8 | | 20.4 | | 20.5 | | 21.0 | | 22.1 | | 22.0 | | 22.1 |
| **PUD** | 1590 | | | 1777 | | 2333 | | 2861 | | 3378 | | 3534 | | 3746 | | 3986 | |  | | 4614 | | 4902 | | 4701 | | 5274 | | 5216 | | 5872 | | 5577 | | 6131 | | 6462 | | 7711 | | 8592 | | 10095 |
| **%‡** | 0.7 | | | 0.8 | | 0.9 | | 1.0 | | 1.1 | | 1.0 | | 1.0 | | 1.1 | |  | | 1.2 | | 1.2 | | 1.1 | | 1.2 | | 1.2 | | 1.3 | | 1.2 | | 1.3 | | 1.4 | | 1.6 | | 1.7 | | 2.0 |

AD: already diagnosed; ND: newly diagnosed; PUD: potential undiagnosed

* patients ≥40 years old who already had a COPD diagnosis, and those who did not have a COPD diagnosis, but had a history of smoking and COPD-like exacerbations (i.e., exacerbations of potential COPD). †Patients with COPD (or potential COPD) who have had 2 or more moderate, or 1 or more severe exacerbations in the last 12 months. ‡ of eligible population;

**S-Table 12: High risk patient characteristics (2000-2004) (based on specific definition of exacerbations)**

|  | **Newly diagnosed** | **Already diagnosed** | **Undiagnosed** |
| --- | --- | --- | --- |
| **N** | 1065 | 11136 | 11939 |
| **Age by index date, mean (SD)** | 68.0 (10.5) | 71.4 (10.0) | 61.6 (12.6) |
| **Age by index date, n (%)** | | | |
| **40 - 49 years** | 51 (4.8) | 253 (2.3) | 2387 (20.0) |
| **50 - 59 years** | 162 (15.2) | 1175 (10.6) | 3081 (25.8) |
| **60 - 69 years** | 358 (33.6) | 3017 (27.1) | 3095 (25.9) |
| **70+ years** | 494 (46.4) | 6691 (60.1) | 3376 (28.3) |
| **Female, n (%)** | 516 (48.5) | 5256 (47.2) | 6729 (56.4) |
| **Ethnicity, n (%)** | | | |
| White | 377 (35.4) | 3442 (30.9) | 5577 (46.7) |
| Mixed/ Multiple ethnic groups | 1 (0.1) | 1 (0.01) | 20 (0.2) |
| Asian / Asian British | 5 (0.5) | 47 (0.4) | 111 (0.9) |
| Black/ African/ Caribbean/ Black British | 1 (0.1) | 11 (0.1) | 19 (0.2) |
| Other ethnic group | 127 (11.9) | 1156 (10.4) | 1125 (9.4) |
| Missing ethnicity | 554 (52.0) | 6479 (58.2) | 5087 (42.6) |
| **Smoking, n (%)** | | | |
| Never-smoker | 61 (5.7) | 687 (6.2) | 0 (0.0) |
| Current smoker | 505 (47.4) | 4188 (37.6) | 8294 (69.5) |
| Former smoker | 416 (39.1) | 5507 (49.5) | 3643 (30.5) |
| Missing smoking status | 83 (7.8) | 754 (6.8) | 0 (0.0) |
| **BMI (within 5 years of index date), n (%)** | | | |
| Underweight (<18.5) | 48 (4.5) | 715 (6.4) | 294 (2.5) |
| Normal weight (18.5-24) | 353 (33.2) | 3590 (32.2) | 3058 (25.6) |
| Overweight (25-29) | 292 (27.4) | 2754 (24.7) | 3600 (30.2) |
| Obese (30.0+) | 202 (19.0) | 1983 (17.8) | 2961 (24.8) |
| Missing BMI | 170 (16.0) | 2094 (18.8) | 2026 (17.0) |
| **BEC within 5 years of index date, mean (SD) count 10^9/L** | - | - | - |
| **Blood eosinophil count (BEC) within 5 years of index date - highest recorded; n (%)** | | | |
| No BEC recorded in period | 1065 (100.0) | 11136 (100.0) | 11939 (100.0) |
| <0.15 (10^9/L) | - | - | - |
| 0.15 <0.30 (10^9/L) | - | - | - |
| 0.30 <0.45 (10^9/L) | - | - | - |
| >= 0.45 (10^9/L) | - | - | - |
| **Number of moderate exacerbations in baseline 12m, Mean (SD)** | 3.2 (2.1) | 3.9 (2.5) | 2.6 (1.5) |
| Oral corticosteroid prescriptions | 0.4 (1.2) | 0.8 (1.8) | 0.1 (0.7) |
| Antibiotic prescriptions | 2.5 (1.7) | 2.3 (1.9) | 2.4 (1.4) |
| OCS and Antibiotic prescriptions | 0.4 (0.8) | 0.9 (1.3) | 0.1 (0.4) |
| **Moderate exacerbations in baseline 12m, n (%)** | | | |
| 0 | 78 (7.3) | 439 (3.9) | 900 (7.5) |
| 1 | 49 (4.6) | 455 (4.1) | 459 (3.8) |
| 2 | 304 (28.5) | 2815 (25.3) | 5487 (46.0) |
| 3 | 275 (25.8) | 2435 (21.9) | 2907 (24.4) |
| 4+ | 359 (33.7) | 4992 (44.8) | 2186 (18.3) |
| **Number of severe exacerbations (hospital admittance for respiratory reason) in baseline 12m, mean (SD)** | 0.2 (0.4) | 0.2 (0.4) | 0.1 (0.4) |
| **Severe exacerbations in baseline 12m, n (%)** | | | |
| 0 | 860 (80.8) | 9616 (86.4) | 10295 (86.2) |
| 1 | 191 (17.9) | 1372 (12.3) | 1611 (13.5) |
| 2+ | 14 (1.3) | 148 (1.3) | 33 (0.3) |
| **Number of moderate exacerbations in follow-up 12m, mean (SD)** | 2.2 (2.3) | 3.3 (3.0) | 1.4 (1.8) |
| Oral corticosteroid prescriptions | 0.4 (1.3) | 0.8 (1.9) | 0.1 (0.7) |
| Antibiotic prescriptions | 1.5 (1.6) | 1.8 (2) | 1.2 (1.5) |
| OCS and Antibiotic prescriptions | 0.4 (0.9) | 0.7 (1.3) | 0.1 (0.4) |
| **Moderate exacerbations in follow-up 12m, n (%)** | | | |
| 0 | 264 (24.8) | 1746 (15.7) | 4474 (37.5) |
| 1 | 246 (23.1) | 1927 (17.3) | 3251 (27.2) |
| 2 | 192 (18.0) | 1741 (15.6) | 1954 (16.4) |
| 3 | 145 (13.6) | 1523 (13.7) | 1064 (8.9) |
| 4+ | 218 (20.5) | 4199 (37.7) | 1196 (10.0) |
| **Number of severe exacerbations (hospital admittance for respiratory reason) in follow-up 12m, mean (SD)** | 0.1 (0.3) | 0.1 (0.4) | 0.02 (0.2) |
| **Severe exacerbations in follow-up 12m, n (%)** | | | |
| 0 | 1007 (94.6) | 10392 (93.3) | 11746 (98.4) |
| 1 | 54 (5.1) | 620 (5.6) | 183 (1.5) |
| 2+ | 4 (0.4) | 124 (1.1) | 10 (0.1) |
| **Number of rescue inhaler prescriptions in baseline 12m, mean (SD)** | 4.3 (7.1) | 11.2 (10.8) | 1.4 (3.7) |
| SABA | 3.1 (4.6) | 6.5 (6.4) | 1.2 (2.9) |
| SAMA | 0.7 (2.6) | 3.0 (4.9) | 0.1 (1.0) |
| SABA/SAMA | 0.6 (2.3) | 1.7 (4.2) | 0.1 (1.0) |
| **Number of rescue inhaler prescriptions in follow-up 12m, mean (SD)** | 7.7 (9.3) | 11.3 (10.9) | 1.6 (4.3) |
| SABA | 4.8 (5.5) | 6.5 (6.5) | 1.3 (3.2) |
| SAMA | 1.6 (3.9) | 3.0 (5.0) | 0.2 (1.2) |
| SABA/SAMA | 1.3 (3.4) | 1.7 (4.2) | 0.2 (1.2) |
| **Major cardiac events in baseline 12m, n (%)** | | | |
| New diagnosis for heart failure | 65 (6.1) | 627 (5.6) | 361 (3.0) |
| Hospitalisation for heart failure | 234 (22.0) | 2356 (21.2) | 1922 (16.1) |
| Revascularization | 1 (0.1) | 14 (0.1) | 39 (0.3) |
| Myocardial Infarction | 12 (1.1) | 204 (1.8) | 158 (1.3) |
| Stroke | 3 (0.3) | 73 (0.7) | 41 (0.3) |
| Any of above | 274 (25.7) | 2874 (25.8) | 2238 (18.8) |
| **Major cardiac events in follow-up 12m, n (%)** | | | |
| New diagnosis for heart failure | 71 (6.7) | 598 (5.4) | 307 (2.6) |
| Hospitalisation for heart failure | 195 (18.3) | 2175 (19.5) | 1472 (12.3) |
| Revascularization | 2 (0.2) | 18 (0.2) | 37 (0.3) |
| Myocardial Infarction | 18 (1.7) | 194 (1.7) | 153 (1.3) |
| Stroke | 7 (0.7) | 83 (0.8) | 57 (0.5) |
| Any of above | 256 (24.0) | 2699 (24.2) | 1833 (15.4) |
| **MRC dyspnoea score recorded in 12 months before index date, mean (SD)** | 2.4 (0.8) | 2.5 (0.9) | 2.3 (0.8) |
| No MRC score, N(%) | 772 (72.5) | 8930 (80.2) | 10551 (88.4) |
| **MRC dyspnoea score recorded in 12 months before index date, n (%)** | | | |
| 1-2 | 217 (74.1) | 1618 (73.4) | 1063 (76.6) |
| 3-5 | 76 (25.9) | 588 (26.7) | 325 (23.4) |
| **Spirometry values recorded in 12 months before index date, mean (SD)** | | | |
| FEV1 % predicted | 58.8 (19.7) | 50.3 (21.1) | 83.6 (20.8) |
| FEV1; litres | 1.5 (0.6) | 1.2 (0.6) | 2.3 (0.8) |
| FVC; litres | 2.5 (0.9) | 2.2 (0.8) | 3.0 (0.9) |
| FEV1/FVC | 0.6 (0.2) | 0.5 (0.2) | 0.8 (0.1) |
| % No spirometry recorded | 853 (80.1) | 9830 (88.3) | 11725 (98.2) |
| **COPD therapy in baseline 12m, n (%)** | | | |
| No COPD therapy | 258 (24.2) | 1117 (10.0) | 7908 (66.2) |
| Reliever only (SABA, SAMA and combinations) | 255 (23.9) | 1372 (12.3) | 1653 (13.9) |
| ICS only (mono) | 385 (36.2) | 4050 (36.4) | 1747 (14.6) |
| LABA only (mono) | 17 (1.6) | 446 (4.0) | 43 (0.4) |
| LAMA only (mono) | 4 (0.4) | 45 (0.4) | 2 (0.02) |
| LABA-ICS (Dual) | 83 (7.8) | 2340 (21.0) | 328 (2.8) |
| LABA-ICS fixed (Dual) | 39 (3.7) | 1142 (10.3) | 191 (1.6) |
| LABA-LAMA (dual) | 1 (0.1) | 29 (0.3) | 0 (0.0) |
| LABA-LAMA fixed (Dual) | 0 (0.0) | 0 (0.0) | 0 (0.0) |
| LAMA-ICS (dual) | 4 (0.4) | 72 (0.7) | 4 (0.03) |
| LABA-LAMA-ICS (triple) | 5 (0.5) | 297 (2.7) | 5 (0.04) |
| LABA-LAMA-ICS fixed (triple) | 0 (0.0) | 0 (0.0) | 0 (0.0) |
| **Clinically diagnosed comorbidities (ever), n (%)** | | | |
| **Steroid related** | | | |
| Diabetes type 2 | 73 (6.9) | 803 (7.2) | 982 (8.2) |
| Osteoporosis | 48 (4.5) | 762 (6.8) | 353 (3.0) |
| Hypertension | 319 (30.0) | 3220 (28.9) | 3235 (27.1) |
| Chronic kidney disease | 11 (1.0) | 182 (1.6) | 151 (1.3) |
| Depression/Anxiety | 311 (29.2) | 3537 (31.8) | 4376 (36.7) |
| Obesity | 201 (18.9) | 2208 (19.8) | 3036 (25.4) |
| **Other** | | | |
| CVD | 240 (22.5) | 2708 (24.3) | 2184 (18.3) |
| Asthma | 268 (25.2) | 4800 (43.1) | 2131 (17.9) |
| OSA | 4 (0.4) | 65 (0.6) | 49 (0.4) |
| GERD | 98 (9.2) | 1573 (14.1) | 1536 (12.9) |
| Lung cancer | 3 (0.3) | 29 (0.3) | 20 (0.2) |
| Anaemia | 35 (3.3 | 508 (4.6) | 482 (4.0) |
| **Cambridge multimorbidity score, mean (SD)** | 2.3 (1.2) | 2.5 (1.2) | 0.8 (1.0) |
| **Hospital admission for any condition, mean (SD)** | | | |
| In baseline 12m | 0.4 (0.8) | 0.4 (0.9) | 0.3 (0.7) |
| In follow-up 12m | 0.3 (0.8) | 0.3 (0.9) | 0.2 (0.7) |

BEC: blood eosinophil count; BMI: body mass index; COPD: chronic obstructive pulmonary disease; CVD: cardiovascular disease; FEV_1_: forced expiratory volume in one second; FVC: forced vital capacity; GERD: gastroesophageal reflux disease; ICS: inhaled corticosteroid; LABA: long-acting β2-agonist; LAMA: long-acting muscarinic antagonist; MRC: Medical Research Council; OCS: oral corticosteroid; OSA: obstructive sleep apnoea; SABA: short-acting β_2_-agonist; SAMA: short-acting muscarinic antagonist; SD: standard deviation

The baseline/follow up periods refer to 12 months pre/post index date. The 5-year periods were generated by aggregating all the records in each individual year and treating them as separate observations. Patients in individual years (2000-2004) were added together into one combined data set covering the whole 5-year period. Patients could be present in multiple years during that period and contribute multiple records to the aggregated analysis. Each patient index date combination was treated as an independent observation.

**S-Table 13: Patient characteristics (2005-2009) (based on specific definition of exacerbations)**

|  | **Newly diagnosed** | **Already diagnosed** | **Undiagnosed** |
| --- | --- | --- | --- |
| **N** | 2211 | 22772 | 20782 |
| **Age by index date, mean (SD)** | 68.8 (10.8) | 71.6 (10.2) | 62.4 (12.8) |
| **Age by index date, n (%)** | | | |
| **40 - 49 years** | 90 (4.1 | 501 (2.2) | 4034 (19.4) |
| **50 - 59 years** | 339 (15.3) | 2285 (10.0) | 4864 (23.4) |
| **60 - 69 years** | 705 (31.9) | 6430 (28.2) | 5604 (27.0) |
| **70+ years** | 1077 (48.7) | 13556 (59.5) | 6280 (30.2) |
| **Female, n (%)** | 1052 (47.6) | 11192 (49.2) | 11595 (55.8) |
| **Ethnicity, n (%)** | | | |
| White | 1336 (60.4) | 13106 (57.6) | 12419 (59.8) |
| Mixed/ Multiple ethnic groups | 2 (0.1) | 16 (0.1) | 59 (0.3) |
| Asian / Asian British | 19 (0.9) | 165 (0.7) | 482 (2.3) |
| Black/ African/ Caribbean/ Black British | 2 (0.1) | 25 (0.1) | 55 (0.3) |
| Other ethnic group | 180 (8.1) | 1566 (6.9) | 1306 (6.3) |
| Missing ethnicity | 672 (30.4) | 7894 (34.7) | 6461 (31.1) |
| **Smoking, n (%)** | | | |
| Never-smoker | 119 (5.4) | 1130 (5.0) | 0 (0.0) |
| Current smoker | 923 (41.8) | 6812 (29.9) | 10333 (49.7) |
| Former smoker | 1122 (50.8) | 14556 (63.9) | 10445 (50.3) |
| Missing smoking status | 47 (2.1) | 274 (1.2) | 0 (0.0) |
| **BMI (within 5 years of index date), n (%)** | | | |
| Underweight (<18.5) | 94 (4.3) | 1588 (7.0) | 485 (2.3) |
| Normal weight (18.5-24) | 773 (35.0) | 7893 (34.7) | 5227 (25.2) |
| Overweight (25-29) | 684 (31.0) | 6746 (29.6) | 6592 (31.7) |
| Obese (30.0+) | 528 (23.9) | 5187 (22.8) | 6466 (31.1) |
| Missing BMI | 132 (6.0) | 1358 (6.0) | 2012 (9.7) |
| **BEC within 5 years of index date, mean (SD) count 10^9/L** | - | - | - |
| **Blood eosinophil count (BEC) within 5 years of index date - highest recorded; n (%)** | | | |
| No BEC recorded in period | 2211 (100.0) | 22772 (100.0) | 20782 (100.0) |
| <0.15 (10^9/L) | - | - | - |
| 0.15 <0.30 (10^9/L) | - | - | - |
| 0.30 <0.45 (10^9/L) | - | - | - |
| >= 0.45 (10^9/L) | - | - | - |
| **Number of moderate exacerbations in baseline 12m, mean (SD)** | 2.8 (1.8) | 3.8 (2.5) | 2.5 (1.5) |
| Oral corticosteroid prescriptions | 0.2 (0.8) | 0.6 (1.6) | 0.1 (0.6) |
| Antibiotic prescriptions | 2.3 (1.6) | 2.2 (1.9) | 2.3 (1.4) |
| OCS and Antibiotic prescriptions | 0.3 (0.7) | 1.1 (1.5) | 0.1 (0.4) |
| **Moderate exacerbations in baseline 12m, n (%)** | | | |
| 0 | 202 (9.1) | 1068 (4.7) | 1704 (8.2) |
| 1 | 125 (5.7) | 1044 (4.6) | 881 (4.2) |
| 2 | 752 (34.0) | 5857 (25.7) | 9913 (47.7) |
| 3 | 561 (25.4) | 4927 (21.6) | 4839 (23.3) |
| 4+ | 571 (25.8) | 9876 (43.4) | 3445 (16.6) |
| **Number of severe exacerbations (hospital admittance for respiratory reason) in baseline 12m, mean (SD)** | 0.2 (0.4) | 0.2 (0.4) | 0.2 (0.4) |
| **Severe exacerbations in baseline 12m, n (%)** | | | |
| 0 | 1750 (79.2) | 19271 (84.6) | 17648 (84.9) |
| 1 | 438 (19.8) | 3174 (13.9) | 3064 (14.7) |
| 2+ | 23 (1.0) | 327 (1.4) | 70 (0.3) |
| **Number of moderate exacerbations in follow-up 12m, mean (SD)** | 1.9 (2.2) | 3.2 (2.9) | 1.3 (1.8) |
| Oral corticosteroid prescriptions | 0.2 (1.0) | 0.6 (1.7) | 0.1 (0.7) |
| Antibiotic prescriptions | 1.3 (1.7) | 1.7 (2.0) | 1.2 (1.6) |
| OCS and Antibiotic prescriptions | 0.3 (0.8) | 0.9 (1.5) | 0.1 (0.4) |
| **Moderate exacerbations in follow-up 12m, n (%)** | | | |
| 0 | 637 (28.8) | 3613 (15.9) | 8301 (39.9) |
| 1 | 592 (26.8) | 4054 (17.8) | 5782 (27.8) |
| 2 | 401 (18.1) | 3728 (16.4 | 3127 (15.1) |
| 3 | 209 (9.5) | 3072 (13.5) | 1668 (8.0) |
| 4+ | 372 (16.8) | 8305 (36.5) | 1904 (9.2) |
| **Number of severe exacerbations (hospital admittance for respiratory reason) in follow-up 12m, mean (SD)** | 0.1 (0.2) | 0.1 (0.4) | 0.02 (0.2) |
| **Severe exacerbations in follow-up 12m, n (%)** | | | |
| 0 | 2101 (95.0) | 21029 (92.4) | 20408 (98.2) |
| 1 | 102 (4.6) | 1476 (6.5) | 358 (1.7) |
| 2+ | 8 (0.4) | 267 (1.2) | 16 (0.1) |
| **Number of rescue inhaler prescriptions in baseline 12m, mean (SD)** | 3.2 (4.8) | 9.9 (9.0) | 1.0 (2.9) |
| SABA | 2.5 (3.7) | 6.8 (6.0) | 0.9 (2.4) |
| SAMA | 0.4 (1.7) | 1.8 (3.8) | 0.1 (0.7) |
| SABA/SAMA | 0.4 (1.4) | 1.3 (3.4) | 0.1 (0.6) |
| **Number of rescue inhaler prescriptions in follow-up 12m, mean (SD)** | 5.8 (6.7) | 9.3 (8.7) | 1.2 (3.3) |
| SABA | 4.1 (4.5) | 6.7 (6.0) | 1.0 (2.7) |
| SAMA | 1.0 (2.7) | 1.7 (3.8) | 0.1 (0.9) |
| SABA/SAMA | 0.8 (2.4) | 0.9 (2.9) | 0.1 (0.7) |
| **Major cardiac events in baseline 12m, n(%)** | | | |
| New diagnosis for heart failure | 74 (3.4) | 760 (3.3) | 490 (2.4) |
| Hospitalisation for heart failure | 512 (23.2) | 5535 (24.3) | 3815 (18.4) |
| Revascularization | 9 (0.4) | 31 (0.1) | 79 (0.4) |
| Myocardial Infarction | 32 (1.5) | 364 (1.6) | 302 (1.5) |
| Stroke | 11 (0.5) | 146 (0.6) | 107 (0.5) |
| Any of above | 560 (25.3) | 6249 (27.4) | 4278 (20.6) |
| **Major cardiac events in follow-up 12m, n (%)** | | | |
| New diagnosis for heart failure | 94 (4.3) | 786 (3.5) | 474 (2.3) |
| Hospitalisation for heart failure | 452 (20.4) | 5277 (23.2) | 3126 (15.0) |
| Revascularization | 6 (0.3) | 24 (0.1) | 47 (0.2) |
| Myocardial Infarction | 38 (1.7) | 353 (1.6) | 227 (1.1) |
| Stroke | 10 (0.5) | 136 (0.6) | 99 (0.5) |
| Any of above | 536 (24.2) | 6030 (26.5) | 3626 (17.5) |
| **MRC dyspnoea score recorded in 12 months before index date, mean (SD)** | 2.4 (0.9) | 2.7 (1.0) | 2.3 (0.7) |
| No MRC score, N(%) | 1332 (60.2) | 13830 (60.7) | 18049 (86.9) |
| **MRC dyspnoea score recorded in 12 months before index date, n (%)** | | | |
| 1-2 | 607 (69.1) | 4614 (51.6) | 2116 (77.4) |
| 3-5 | 272 (30.9) | 4328 (48.4) | 617 (22.6) |
| **Spirometry values recorded in 12 months before index date, mean (SD)** | | | |
| FEV1 % predicted | 60.1 (17.8) | 52.3 (19.6) | 82.9 (19.6) |
| FEV1; litres | 1.6 (0.6) | 1.3 (0.6) | 2.2 (0.7) |
| FVC; litres | 2.6 (0.9) | 2.3 (0.9) | 3.0 (0.9) |
| FEV1/FVC | 0.6 (0.1) | 0.6 (0.2) | 0.8 (0.1) |
| % No spirometry recorded | 815 (36.9) | 9150 (40.2) | 19118 (92.0) |
| **COPD therapy in baseline 12m, N (%)** | | | |
| No COPD therapy | 516 (23.3) | 1376 (6.0) | 14254 (68.6) |
| Reliever only (SABA, SAMA and combinations) | 651 (29.4) | 2042 (9.0) | 3415 (16.4) |
| ICS only (mono) | 405 (18.3) | 2806 (12.3) | 1910 (9.2) |
| LABA only (mono) | 46 (2.1) | 542 (2.4) | 59 (0.3) |
| LAMA only (mono) | 76 (3.4) | 537 (2.4) | 40 (0.2) |
| LABA-ICS (Dual) | 86 (3.9) | 1871 (8.2) | 192 (0.9) |
| LABA-ICS fixed (Dual) | 310 (14.0) | 5897 (25.9) | 773 (3.7) |
| LABA-LAMA (dual) | 5 (0.2) | 230 (1.0) | 2 (0.01) |
| LABA-LAMA fixed (Dual) | 0 (0.0) | 0 (0.0) | 0 (0.0) |
| LAMA-ICS (dual) | 31 (1.4) | 575 (2.5) | 24 (0.1) |
| LABA-LAMA-ICS (triple) | 81 (3.7) | 6729 (29.6) | 69 (0.3) |
| LABA-LAMA-ICS fixed (triple) | 0 (0.0) | 0 (0.0) | 0 (0.0) |
| **Clinically diagnosed comorbidities (ever), n(%)** | | | |
| **Steroid related** | | | |
| Diabetes type 2 | 205 (9.3) | 2718 (11.9) | 2738 (13.2) |
| Osteoporosis | 119 (5.4) | 2118 (9.3) | 768 (3.7) |
| Hypertension | 845 (38.2) | 8693 (38.2) | 7205 (34.7) |
| Chronic kidney disease | 160 (7.2) | 2094 (9.2) | 1474 (7.1) |
| Depression/Anxiety | 705 (31.9) | 8609 (37.8) | 8526 (41.0) |
| Obesity | 603 (27.3) | 6648 (29.2) | 7112 (34.2) |
| **Other** | | | |
| CVD | 542 (24.5) | 6161 (27.1) | 4207 (20.2) |
| Asthma | 356 (16.1) | 10239 (45.0) | 2827 (13.6) |
| OSA | 12 (0.5) | 300 (1.3) | 184 (0.9) |
| GERD | 276 (12.5) | 3811 (16.7) | 3181 (15.3) |
| Lung cancer | 6 (0.3) | 70 (0.3) | 39 (0.2) |
| Anaemia | 103 (4.7) | 1395 (6.1) | 981 (4.7) |
| **Cambridge multimorbidity score, mean (SD)** | 2.4 (1.1) | 2.7 (1.2) | 1.0 (1.1) |
| **Hospital admission for any condition, mean (SD)** | | | |
| In baseline 12m | 0.4 (0.8) | 0.4 (0.9) | 0.3 (0.7) |
| In follow-up 12m | 0.3 (0.8) | 0.4 (1.0) | 0.2 (0.7) |

BEC: blood eosinophil count; BMI: body mass index; COPD: chronic obstructive pulmonary disease; CVD: cardiovascular disease; FEV_1_: forced expiratory volume in one second; FVC: forced vital capacity; GERD: gastroesophageal reflux disease; ICS: inhaled corticosteroid; LABA: long-acting β2-agonist; LAMA: long-acting muscarinic antagonist; MRC: Medical Research Council; OCS: oral corticosteroid; OSA: obstructive sleep apnoea; SABA: short-acting β_2_-agonist; SAMA: short-acting muscarinic antagonist; SD: standard deviation

The baseline/follow up periods refer to 12 months pre/post index date. The 5-year periods were generated by aggregating all the records in each individual year and treating them as separate observations Patients in individual years (2005-2009) were added together into one combined data set covering the whole 5-year period. Patients could be present in multiple years during that period and contribute multiple records to the aggregated analysis. Each patient index date combination was treated as an independent observation.

**S-Table 14: Patient characteristics (2010-2014) (based on specific definition of exacerbations)**

|  | **Newly diagnosed** | **Already diagnosed** | **Undiagnosed** |
| --- | --- | --- | --- |
| **N** | 3064 | 36382 | 26640 |
| **Age by index date, mean (SD)** | 67.9 (11.4) | 71.4 (10.3) | 62.8 (13.4) |
| **Age by index date, n (%)** | | | |
| **40 - 49 years** | 177 (5.8) | 821 (2.3) | 5216 (19.6) |
| **50 - 59 years** | 517 (16.9) | 3883 (10.7) | 6064 (22.8) |
| **60 - 69 years** | 1003 (32.7) | 10704 (29.4) | 6880 (25.8) |
| **70+ years** | 1367 (44.6) | 20974 (57.7) | 8480 (31.8) |
| **Female, n (%)** | 1511 (49.3) | 18353 (50.5) | 14547 (54.6) |
| **Ethnicity, n (%)** | | | |
| White | 2115 (69.0) | 25991 (71.4) | 18047 (67.7) |
| Mixed/ Multiple ethnic groups | 9 (0.3) | 51 (0.1) | 102 (0.4) |
| Asian / Asian British | 53 (1.7) | 368 (1.0) | 905 (3.4) |
| Black/ African/ Caribbean/ Black British | 5 (0.2) | 49 (0.1) | 120 (0.5) |
| Other ethnic group | 156 (5.1) | 1803 (5.0) | 1406 (5.3) |
| Missing ethnicity | 726 (23.7) | 8120 (22.3) | 6060 (22.8) |
| **Smoking, n (%)** | | | |
| Never-smoker | 120 (3.9) | 1384 (3.8) | 0 (0.0) |
| Current smoker | 1326 (43.3) | 11400 (31.3) | 11842 (44.5) |
| Former smoker | 1568 (51.2) | 23162 (63.7) | 14784 (55.5) |
| Missing smoking status | 50 (1.6) | 436 (1.2) | 0 (0.0) |
| **BMI (within 5 years of index date), n (%)** | | | |
| Underweight (<18.5) | 129 (4.2) | 2142 (5.9) | 552 (2.1) |
| Normal weight (18.5-24) | 949 (31.0) | 11955 (32.9) | 6296 (23.6) |
| Overweight (25-29) | 995 (32.5) | 11408 (31.4) | 8466 (31.8) |
| Obese (30.0+) | 905 (29.5) | 9988 (27.5) | 9412 (35.3) |
| Missing BMI | 86 (2.8) | 889 (2.4) | 1914 (7.2) |
| **BEC within 5 years of index date, mean (SD) count 10^9/L** | - | - | - |
| **Blood eosinophil count (BEC) within 5 years of index date - highest recorded; n (%)** | | | |
| No BEC recorded in period | 3064 (100.0) | 36382 (100.0) | 26640 (100.0) |
| <0.15 (10^9/L) | - | - | - |
| 0.15 <0.30 (10^9/L) | - | - | - |
| 0.30 <0.45 (10^9/L) | - | - | - |
| >= 0.45 (10^9/L) | - | - | - |
| **Number of moderate exacerbations in baseline 12m, Mean (SD)** | 2.8 (1.9) | 3.7 (2.6) | 2.4 (1.6) |
| Oral corticosteroid prescriptions | 0.2 (0.8) | 0.5 (1.3) | 0.1 (0.7 |
| Antibiotic prescriptions | 2.2 (1.6) | 1.9 (2.0) | 2.2 (1.5) |
| OCS and Antibiotic prescriptions | 0.5 (0.9) | 1.4 (1.7) | 0.2 (0.5) |
| **Moderate exacerbations in baseline 12m, n (%)** | | | |
| 0 | 291 (9.5) | 2343 (6.4) | 3057 (11.5) |
| 1 | 223 (7.3) | 2899 (8.0) | 1583 (5.9) |
| 2 | 991 (32.3) | 8666 (23.8) | 11785 (44.2) |
| 3 | 768 (25.1) | 7204 (19.8) | 5810 (21.8) |
| 4+ | 791 (25.8) | 15270 (42.0) | 4405 (16.5) |
| **Number of severe exacerbations (hospital admittance for respiratory reason) in baseline 12m, mean (SD)** | 0.3 (0.5) | 0.2 (0.5) | 0.2 (0.4) |
| **Severe exacerbations in baseline 12m, n (%)** | | | |
| 0 | 2296 (74.9) | 29818 (82.0) | 21078 (79.1) |
| 1 | 721 (23.5) | 5903 (16.2) | 5430 (20.4) |
| 2+ | 47 (1.5) | 661 (1.8) | 132 (0.5) |
| **Number of moderate exacerbations in follow-up 12m, Mean (SD)** | 1.9 (2.2) | 3.3 (3.0) | 1.3 (1.8) |
| Oral corticosteroid prescriptions | 0.2 (0.9) | 0.5 (1.4) | 0.1 (0.7) |
| Antibiotic prescriptions | 1.3 (1.7) | 1.6 (2.1) | 1.1 (1.6) |
| OCS and Antibiotic prescriptions | 0.5 (1.1) | 1.2 (1.8) | 0.1 (0.5) |
| **Moderate exacerbations in follow-up 12m, n (%)** | | | |
| 0 | 924 (30.2) | 5850 (16.1) | 11050 (41.5) |
| 1 | 728 (23.8) | 6452 (17.7) | 7063 (26.5) |
| 2 | 536 (17.5) | 5887 (16.2) | 3974 (14.9) |
| 3 | 355 (11.6) | 4793 (13.2) | 2043 (7.7) |
| 4+ | 521 (17.0) | 13400 (36.8) | 2510 (9.4) |
| **Number of severe exacerbations (hospital admittance for respiratory reason) in follow-up 12m, mean (SD)** | 0.1 (0.3) | 0.1 (0.4) | 0.03 (0.2) |
| **Severe exacerbations in follow-up 12m, n (%)** | | | |
| 0 | 2863 (93.4) | 32769 (90.1) | 25928 (97.3) |
| 1 | 186 (6.1) | 3086 (8.5) | 651 (2.4) |
| 2+ | 15 (0.5) | 527 (1.5) | 61 (0.2) |
| **Number of rescue inhaler prescriptions in baseline 12m, mean (SD)** | 2.6 (3.7) | 7.5 (6.7) | 1.0 (2.5) |
| SABA | 2.4 (3.3) | 6.6 (5.6) | 0.9 (2.4) |
| SAMA | 0.2 (1.3) | 1.0 (3.0) | 0.1 (0.6) |
| SABA/SAMA | 0.01 (0.2) | 0.0 (0.1 | 0.0 (0.02) |
| **Number of rescue inhaler prescriptions in follow-up 12m, mean (SD)** | 4.4 (4.9) | 7.5 (6.7) | 1.1 (2.9) |
| SABA | 4.0 (4.3) | 6.6 (5.6) | 1.03 (2.7) |
| SAMA | 0.4 (1.7) | 0.8 (2.8) | 0.1 (0.7) |
| SABA/SAMA | 0.0 (0.0) | 0.0 (0.02) | 0.0 (0.0) |
| **Major cardiac events in baseline 12m, n(%)** | | | |
| New diagnosis for heart failure | 102 (3.3) | 1148 (3.2) | 691 (2.6) |
| Hospitalisation for heart failure | 995 (32.5) | 12389 (34.1) | 8295 (31.1) |
| Revascularization | 8 (0.3) | 39 (0.1) | 60 (0.2) |
| Myocardial Infarction | 42 (1.4) | 521 (1.4) | 396 (1.5) |
| Stroke | 17 (0.6) | 194 (0.5) | 132 (0.5) |
| Any of above | 1056 (34.5) | 13186 (36.2) | 8715 (32.7) |
| **Major cardiac events in follow-up 12m, n(%)** | | | |
| New diagnosis for heart failure | 112 (3.7) | 1135 (3.1) | 650 (2.4) |
| Hospitalisation for heart failure | 872 (28.5) | 12561 (34.5) | 6896 (25.9) |
| Revascularization | 4 (0.1) | 37 (0.1) | 36 (0.1) |
| Myocardial Infarction | 46 (1.5) | 508 (1.4) | 272 (1.0) |
| Stroke | 12 (0.4) | 200 (0.6) | 118 (0.4) |
| Any of above | 956 (31.2) | 13273 (36.5) | 7375 (27.7) |
| **MRC dyspnoea score recorded in 12 months before index date, mean (SD)** | 2.2 (0.9) | 2.8 (1.1) | 2.2 (0.8) |
| No MRC score, N(%) | 1303 (42.5) | 7352 (20.2) | 23039 (86.5) |
| **MRC dyspnoea score recorded in 12 months before index date, n (%)** | | | |
| 1-2 | 1221 (69.3) | 12570 (43.3) | 2742 (76.2) |
| 3-5 | 540 (30.7) | 16460 (56.7) | 859 (23.8) |
| **Spirometry values recorded in 12 months before index date, mean (SD)** | | | |
| FEV1 % predicted | 65.3 (18.3) | 56.3 (20.0) | 84.5 (19.3) |
| FEV1; litres | 1.7 (0.6) | 1.4 (0.6) | 2.3 (0.8) |
| FVC; litres | 2.7 (0.9) | 2.5 (0.9) | 3.1 (1.0) |
| FEV1/FVC | 0.6 (0.1) | 0.6 (0.2) | 0.8 (0.1) |
| % No spirometry recorded | 939 (30.7) | 11220 (30.84) | 24223 (90.9) |
| **COPD therapy in baseline 12m, N (%)** | | | |
| No COPD therapy | 636 (20.8) | 1645 (4.5) | 17893 (67.2) |
| Reliever only (SABA, SAMA and combinations) | 815 (26.6) | 2207 (6.1) | 4920 (18.5) |
| ICS only (mono) | 327 (10.7) | 1482 (4.1) | 1910 (7.2) |
| LABA only (mono) | 63 (2.1) | 492 (1.4) | 27 (0.1) |
| LAMA only (mono) | 309 (10.1) | 2204 (6.1) | 157 (0.6) |
| LABA-ICS (Dual) | 41 (1.3) | 550 (1.5) | 147 (0.6) |
| LABA-ICS fixed (Dual) | 469 (15.3) | 6606 (18.2) | 1290 (4.8) |
| LABA-LAMA (dual) | 12 (0.4) | 589 (1.6) | 9 (0.03) |
| LABA-LAMA fixed (Dual) | 0 (0.0) | 0 (0.0) | 0 (0.00) |
| LAMA-ICS (dual) | 74 (2.4) | 815 (2.2) | 46 (0.2) |
| LABA-LAMA-ICS (triple) | 311 (10.2) | 19695 (54.1) | 200 (0.8) |
| LABA-LAMA-ICS fixed (triple) | 0 (0.0) | 0 (0.0) | 0 (0.0) |
| **Clinically diagnosed comorbidities (ever), n(%)** | | | |
| **Steroid related** |  |  |  |
| Diabetes type 2 | 403 (13.2) | 5476 (15.1) | 4446 (16.7) |
| Osteoporosis | 180 (5.9) | 3874 (10.7) | 1161 (4.4) |
| Hypertension | 1274 (41.6) | 15901 (43.7) | 10005 (37.6) |
| Chronic kidney disease | 433 (14.1) | 5658 (15.6) | 3240 (12.2) |
| Depression/Anxiety | 1193 (38.9) | 15873 (43.6) | 12501 (46.9) |
| Obesity | 1105 (36.1) | 13838 (38.0) | 11263 (42.3) |
| **Other** |  |  |  |
| CVD | 698 (22.8) | 9499 (26.1) | 5178 (19.4) |
| Asthma | 414 (13.5) | 15248 (41.9) | 3553 (13.3) |
| OSA | 50 (1.6) | 796 (2.2) | 490 (1.8) |
| GERD | 497 (16.2) | 7528 (20.7) | 5284 (19.8) |
| Lung cancer | 14 (0.5) | 146 (0.4) | 48 (0.2) |
| Anaemia | 174 (5.7) | 2787 (7.7) | 1833 (6.9) |
| **Cambridge multimorbidity score, mean (SD)** | 2.5 (1.3) | 2.8 (1.3) | 1.2 (1.3) |
| **Hospital admission for any condition, mean (SD)** |  |  |  |
| In baseline 12m | 0.5 (0.9) | 0.6 (1.1) | 0.5 (1.0) |
| In follow-up 12m | 0.5 (1.0) | 0.6 (1.2) | 0.4 (0.9) |

BEC: blood eosinophil count; BMI: body mass index; COPD: chronic obstructive pulmonary disease; CVD: cardiovascular disease; FEV_1_: forced expiratory volume in one second; FVC: forced vital capacity; GERD: gastroesophageal reflux disease; ICS: inhaled corticosteroid; LABA: long-acting β_2_-agonist; LAMA: long-acting muscarinic antagonist; MRC: Medical Research Council; OCS: oral corticosteroid; OSA: obstructive sleep apnoea; SABA: short-acting β_2_-agonist; SAMA: short-acting muscarinic antagonist; SD: standard deviation

The baseline/follow up periods refer to 12 months pre/post index date. The 5-year periods were generated by aggregating all the records in each individual year and treating them as separate observations. Patients in individual years (2010-2014) were added together into one combined data set covering the whole 5-year period. Patients could be present in multiple years during that period and contribute multiple records to the aggregated analysis. Each patient index date combination was treated as an independent observation.

**S-Table 15: Patient characteristics (2015-2019) (based on specific definition of exacerbations)**

|  | **Newly diagnosed** | **Already diagnosed** | **Undiagnosed** |
| --- | --- | --- | --- |
| **N** | 3659 | 48808 | 38991 |
| **Age by index date, mean (SD)** | 68.6 (11.4) | 71.8 (10.4) | 64.1(14.0) |
| **Age by index date, n (%)** | | | |
| **40 - 49 years** | 225 (6.2) | 1033 (2.1) | 7029 (18.0) |
| **50 - 59 years** | 559 (15.3) | 5384 (11.0) | 8945 (22.9) |
| **60 - 69 years** | 1069 (29.2) | 12894 (26.4) | 8796 (22.6) |
| **70+ years** | 1806 (49.4) | 29497 (60.4) | 14221 (36.5) |
| **Female, n (%)** | 1792 (49.0) | 24907 (51.0) | 20967 (53.8) |
| **Ethnicity, n (%)** | | | |
| White | 2653 (72.5) | 36446 (74.7) | 27475 (70.5) |
| Mixed/ Multiple ethnic groups | 14 (0.4) | 96 (0.2) | 173 (0.4) |
| Asian / Asian British | 70 (1.9) | 690 (1.4) | 1471 (3.8) |
| Black/ African/ Caribbean/ Black British | 5 (0.1) | 90 (0.2) | 187 (0.5) |
| Other ethnic group | 142 (3.9) | 2219 (4.6) | 1555 (4.0) |
| Missing ethnicity | 775 (21.2) | 9267 (19.0) | 8130 (20.9) |
| **Smoking, n (%)** | | | |
| Never-smoker | 166 (4.5) | 1785 (3.7) | 0 (0.0) |
| Current smoker | 1548 (42.3) | 15437 (31.6) | 14805 (38.0) |
| Former smoker | 1930 (52.8) | 31414 (64.4) | 24159 (62.0) |
| Missing smoking status | 15 (0.4) | 172 (0.4) | 0 (0.0) |
| **BMI (within 5 years of index date), n (%)** | | | |
| Underweight (<18.5) | 141 (3.9) | 2771 (5.7) | 803 (2.1) |
| Normal weight (18.5-24) | 1072 (29.3) | 15729 (32.2) | 9271 (23.8) |
| Overweight (25-29) | 1159 (31.7) | 14954 (30.6) | 11909 (30.5) |
| Obese (30.0+) | 1180 (32.3) | 14434 (29.6) | 13805 (35.4) |
| Missing BMI | 107 (2.9) | 920 (1.9) | 3203 (8.2) |
| **BEC within 5 years of index date, mean (SD) count 10^9/L** | - | - | - |
| **Blood eosinophil count (BEC) within 5 years of index date - highest recorded; n (%)** | | | |
| No BEC recorded in period | 3659 (100.0) | 48808 (100.0) | 38991 (100.0) |
| <0.15 (10^9/L) | - | - | - |
| 0.15 <0.30 (10^9/L) | - | - | - |
| 0.30 <0.45 (10^9/L) | - | - | - |
| >= 0.45 (10^9/L) | - | - | - |
| **Number of moderate exacerbations in baseline 12m, Mean (SD)** | 2.6 (2.0) | 3.6 (2.8) | 1.9 (1.8) |
| Oral corticosteroid prescriptions | 0.2 (0.8) | 0.4 (1.2) | 0.1 (0.7) |
| Antibiotic prescriptions | 1.8 (1.7) | 1.7 (2.1) | 1.7 (1.6) |
| OCS and Antibiotic prescriptions | 1.6 (1.8) | 0.6 (1.0) | 0.2 (0.5) |
| **Moderate exacerbations in baseline 12m, n (%)** | | | |
| 0 | 560 (15.3) | 4242 (8.7) | 10949 (28.1) |
| 1 | 375 (10.3) | 5049 (10.3) | 4190 (10.8) |
| 2 | 1135 (31.0) | 11002 (22.5) | 13200 (33.9) |
| 3 | 752 (20.6) | 8926 (18.3) | 5886 (15.1) |
| 4+ | 837 (22.9) | 19589 (40.1) | 4766 (12.2) |
| **Number of severe exacerbations (hospital admittance for respiratory reason) in baseline 12m, mean (SD)** | 0.4 (0.6) | 0.3 (0.5) | 0.5 (0.6) |
| **Severe exacerbations in baseline 12m, n (%)** | | | |
| 0 | 2365 (64.6) | 37737 (77.3) | 21672 (55.6) |
| 1 | 1185 (32.4) | 9741 (20.0) | 16460 (42.2) |
| 2+ | 109 (3.0) | 1330 (2.7) | 859 (2.2) |
| **Number of moderate exacerbations in follow-up 12m, Mean (SD)** | 1.9 (2.3) | 3.2 (3.1) | 1.1 (1.8) |
| Oral corticosteroid prescriptions | 0.2 (0.9) | 0.4 (1.3) | 0.1 (0.8) |
| Antibiotic prescriptions | 1.0 (1.6) | 1.5 (2.2) | 0.9 (1.6) |
| OCS and Antibiotic prescriptions | 0.6 (1.3) | 1.3 (1.9) | 0.1 (0.5) |
| **Moderate exacerbations in follow-up 12m, n (%)** | | | |
| 0 | 1230 (33.6) | 8888 (18.2) | 19956 (51.2) |
| 1 | 888 (24.3) | 9203 (18.9) | 9534 (24.5) |
| 2 | 582 (15.9) | 7744 (15.9) | 4390 (11.3) |
| 3 | 356 (9.7) | 6036 (12.4) | 2170 (5.6) |
| 4+ | 603 (16.5) | 16937 (34.7) | 2941 (7.5) |
| **Number of severe exacerbations (hospital admittance for respiratory reason) in follow-up 12m, mean (SD)** | 0.1 (0.4) | 0.2 (0.5) | 0.1 (0.3) |
| **Severe exacerbations in follow-up 12m, n (%)** | | | |
| 0 | 3303 (90.3) | 42720 (87.5) | 36503 (93.6) |
| 1 | 307 (8.4) | 4894 (10.0) | 2140 (5.5) |
| 2+ | 49 (1.3) | 1194 (2.5) | 348 (0.9) |
| **Number of rescue inhaler prescriptions in baseline 12m, mean (SD)** | 2.6 (3.7) | 7.0 (6.0) | 0.8 (2.3) |
| SABA | 2.5 (3.4) | 6.6 (5.5) | 0.8 (2.3) |
| SAMA | 0.1 (0.9) | 0.4 (1.8) | 0.02 (0.4) |
| SABA/SAMA | 0.0 (0.0) | 0.0 (0.0) | 0.0 (0.0) |
| **Number of rescue inhaler prescriptions in follow-up 12m, mean (SD)** | 4.4 (4.8) | 6.8 (6.0) | 0.9 (2.6) |
| SABA | 4.2 (4.7) | 6.5 (5.4) | 0.9 (2.5) |
| SAMA | 0.1 (1.0) | 0.4 (1.9) | 0.02 (0.4) |
| SABA/SAMA | 0.0 (0.0) | 0.0 (0.0) | 0.0 (0.0) |
| **Major cardiac events in baseline 12m, n(%)** | | | |
| New diagnosis for heart failure | 169 (4.6) | 1823 (3.7) | 1466 (3.8) |
| Hospitalisation for heart failure | 1764 (48.2) | 22959 (47.0) | 21755 (55.8) |
| Revascularization | 6 (0.2) | 33 (0.07) | 66 (0.2) |
| Myocardial Infarction | 56 (1.5) | 674 (1.4) | 831 (2.1) |
| Stroke | 18 (0.5) | 316 (0.7) | 265 (0.7) |
| Any of above | 1803 (49.3) | 23548 (48.3) | 22115 (56.7) |
| **Major cardiac events in follow-up 12m, n(%)** | | | |
| New diagnosis for heart failure | 178 (4.9) | 1948 (4.0) | 1268 (3.3) |
| Hospitalisation for heart failure | 1494 (40.8) | 21228 (43.5) | 14131 (36.2) |
| Revascularization | 4 (0.1) | 21 (0.04) | 40 (0.1) |
| Myocardial Infarction | 36 (1.0) | 637 (1.3) | 407 (1.0) |
| Stroke | 24 (0.7) | 297 (0.6) | 214 (0.6) |
| Any of above | 1558 (42.6) | 21957 (45.0) | 14738 (37.8) |
| **MRC dyspnoea score recorded in 12 months before index date, mean (SD)** | 2.3 (0.9) | 2.8 (1.0) | 2.2 (0.8) |
| No MRC score, N(%) | 1783 (48.7) | 8242 (16.9) | 33926 (87.0) |
| **MRC dyspnoea score recorded in 12 months before index date, n (%)** | | | |
| 1-2 | 1224 (65.3) | 16471 (40.6) | 3729 (73.6) |
| 3-5 | 652 (34.8) | 24095 (59.4) | 1336 (26.4) |
| **Spirometry values recorded in 12 months before index date, mean (SD)** | | | |
| FEV1 % predicted | 67.6 (19.3) | 58.8 (20.6) | 85.7 (19.6) |
| FEV1; litres | 1.8 (0.7) | 1.5 (0.6) | 2.3 (0.8) |
| FVC; litres | 2.8 (0.9) | 2.5 (0.9) | 3.0 (0.9) |
| FEV1/FVC | 0.6 (0.1) | 0.6 (0.2) | 0.8 (0.1) |
| % No spirometry recorded | 1333 (36.4) | 15276 (31.3) | 36006 (92.3) |
| **COPD therapy in baseline 12m, N (%)** |  |  |  |
| No COPD therapy | 753 (20.6) | 2437 (5.0) | 28217 (72.4) |
| Reliever only (SABA, SAMA and combinations) | 942 (25.7) | 2320 (4.8) | 6082 (15.6) |
| ICS only (mono) | 259 (7.1) | 846 (1.7) | 2035 (5.2) |
| LABA only (mono) | 63 (1.7) | 533 (1.1) | 47 (0.1) |
| LAMA only (mono) | 538 (14.7) | 3667 (7.5) | 326 (0.8) |
| LABA-ICS (Dual) | 11 (0.3) | 235 (0.5) | 71 (0.2) |
| LABA-ICS fixed (Dual) | 462 (12.6) | 6409 (13.1) | 1629 (4.2) |
| LABA-LAMA (dual) | 12 (0.3) | 690 (1.4) | 8 (0.02) |
| LABA-LAMA fixed (Dual) | 151 (4.1) | 2235 (4.6) | 70 (0.2) |
| LAMA-ICS (dual) | 97 (2.7) | 543 (1.1) | 59 (0.2) |
| LABA-LAMA-ICS (triple) | 355 (9.7) | 27328 (56.0) | 353 (0.9) |
| LABA-LAMA-ICS fixed (triple) | 12 (0.3) | 1504 (318) | 17 (0.04) |
| **Clinically diagnosed comorbidities (ever), n(%)** | | | |
| **Steroid related** |  |  |  |
| Diabetes type 2 | 599 (16.4) | 9274 (19.0) | 7268 (18.6) |
| Osteoporosis | 272 (7.4) | 5987 (12.3) | 2322 (6.0) |
| Hypertension | 1600 (43.7) | 22696 (46.5) | 15753 (40.4) |
| Chronic kidney disease | 539 (14.7) | 8023 (16.4) | 5170 (13.3) |
| Depression/Anxiety | 1524 (41.7) | 23906 (49.0) | 19721 (50.6) |
| Obesity | 1520 (41.5) | 21052 (43.1) | 18019 (46.2) |
| **Other** |  |  |  |
| CVD | 834 (22.8) | 12562 (25.7) | 7943 (20.4) |
| Asthma | 441 (12.1) | 17939 (36.8) | 4948 (12.7) |
| OSA | 92 (2.5) | 1610 (3.3) | 1087 (2.8) |
| GERD | 690 (18.9) | 11633 (23.8) | 8499 (21.8) |
| Lung cancer | 16 (0.4) | 353 (0.7) | 107 (0.3) |
| Anaemia | 280 (7.7) | 4582 (9.4) | 3438 (8.8) |
| **Cambridge multimorbidity score, mean (SD)** | 2.7 (1.4) | 3.0 (1.4) | 1.3 (1.4) |
| **Hospital admission for any condition, mean (SD)** | | | |
| In baseline 12m | 0.9 (1.2) | 0.9 (1.3) | 1.0 (1.3) |
| In follow-up 12m | 0.7 (1.2) | 0.9 (1.4) | 0.7 (1.2) |

BEC: blood eosinophil count; BMI: body mass index; COPD: chronic obstructive pulmonary disease; CVD: cardiovascular disease; FEV_1_: forced expiratory volume in one second; FVC: forced vital capacity; GERD: gastroesophageal reflux disease; ICS: inhaled corticosteroid; LABA: long-acting β_2_-agonist; LAMA: long-acting muscarinic antagonist; MRC: Medical Research Council; OCS: oral corticosteroid; OSA: obstructive sleep apnoea; SABA: short-acting β_2_-agonist; SAMA: short-acting muscarinic antagonist; SD: standard deviation

The baseline/follow up periods refer to 12 months pre/post index date. The 5-year periods were generated by aggregating all the records in each individual year and treating them as separate observations. Patients in individual years (2015-2019) were added together into one combined data set covering the whole 5-year period. Patients could be present in multiple years during that period and contribute multiple records to the aggregated analysis. Each patient index date combination was treated as an independent observation.

S-Table 16: Percentage of high-risk **newly diagnosed patients with COPD** who met relevant CONQUEST quality standards from 2000-2019 (based on specific definition of exacerbations)

| Year | 2000 | 2001 | 2002 | 2003 | 2004 | 2005 | 2006 | 2007 | 2008 | 2009 | 2010 | 2011 | 2012 | 2013 | 2014 | 2015 | 2016 | 2017 | 2018 | 2019 |
| --- | --- | --- | --- | --- | --- | --- | --- | --- | --- | --- | --- | --- | --- | --- | --- | --- | --- | --- | --- | --- |
| N | 157 | 194 | 205 | 232 | 277 | 404 | 414 | 429 | 462 | 502 | 544 | 537 | 663 | 676 | 644 | 685 | 667 | 735 | 790 | 782 |
| **Spirometry*** | 13.4 | 12.4 | 11.2 | 22.4 | 33.2 | 50.2 | 64.5 | 64.8 | 66.9 | 67.5 | 66.7 | 66.5 | 70.4 | 70.6 | 71.6 | 68.0 | 65.7 | 62.2 | 64.4 | 58.3 |
| **Spirometry†** | 25.5 | 34.0 | 32.7 | 49.1 | 72.2 | 86.1 | 90.1 | 87.4 | 88.7 | 89.4 | 86.8 | 89.0 | 91.4 | 91.1 | 90.7 | 89.3 | 87.7 | 88.0 | 87.6 | 83.5 |
| **Exacerbation history†** | 0.0 | 0.0 | 0.0 | 0.0 | 0.0 | 0.0 | 1.9 | 3.0 | 4.3 | 6.6 | 13.2 | 22.5 | 28.4 | 25.7 | 31.4 | 33.3 | 35.8 | 37.3 | 39.9 | 42.2 |
| **Cardiac risk†§** | 0.6 | 2.1 | 5.9 | 6.0 | 7.6 | 7.9 | 7.2 | 7.9 | 8.2 | 11.6 | 18.9 | 20.7 | 20.7 | 19.4 | 24.7 | 21.9 | 21.1 | 23.5 | 19.9 | 20.3 |
| **CAT†** | 0.0 | 0.0 | 0.0 | 0.0 | 0.0 | 0.0 | 0.2 | 0.2 | 0.0 | 0.0 | 0.2 | 3.2 | 10.9 | 12.4 | 15.7 | 12.3 | 17.7 | 19.9 | 26.1 | 26.5 |
| **mMRC†** | 38.9 | 34.5 | 26.8 | 37.1 | 41.5 | 49.0 | 52.4 | 52.4 | 57.6 | 63.9 | 84.6 | 89.0 | 89.4 | 86.7 | 90.4 | 86.0 | 87.0 | 88.0 | 88.5 | 82.7 |
| **Smoking Status*** | 30.6 | 45.9 | 46.3 | 50.4 | 63.5 | 82.2 | 88.4 | 88.6 | 90.5 | 89.4 | 89.7 | 87.0 | 91.3 | 88.6 | 89.0 | 89.8 | 88.2 | 87.5 | 85.2 | 86.4 |
| **Treatment*** |  |  |  |  |  |  |  |  |  |  |  |  |  |  |  |  |  |  |  |  |
| None | 28.0 | 25.3 | 23.9 | 25.4 | 20.6 | 28.5 | 22.0 | 21.4 | 20.8 | 24.3 | 23.0 | 20.7 | 18.1 | 21.2 | 21.3 | 19.6 | 20.2 | 22.7 | 19.0 | 21.4 |
| Reliever only | 26.8 | 20.1 | 20.0 | 23.3 | 28.5 | 29.7 | 27.8 | 30.5 | 30.5 | 28.7 | 27.8 | 28.5 | 27.1 | 25.4 | 24.7 | 27.0 | 26.2 | 24.4 | 25.1 | 26.2 |
| Theophylline | 2.5 | 0.5 | 0.5 | 2.6 | 0.7 | 0.0 | 0.0 | 0.2 | 0.2 | 0.2 | 0.2 | 0.2 | 0.0 | 0.0 | 0.0 | 0.0 | 0.0 | 0.0 | 0.0 | 0.0 |
| ICS | 35.0 | 43.8 | 42.4 | 32.8 | 29.6 | 21.0 | 19.6 | 21.4 | 18.6 | 12.2 | 11.0 | 12.5 | 10.9 | 9.9 | 9.5 | 10.7 | 8.1 | 5.7 | 5.3 | 6.1 |
| LABA | 0.6 | 2.1 | 1.5 | 2.2 | 1.4 | 3.0 | 2.9 | 2.1 | 1.1 | 1.6 | 1.1 | 1.3 | 2.6 | 2.5 | 2.5 | 2.2 | 3.0 | 1.5 | 1.1 | 1.0 |
| LAMA | 0.0 | 0.0 | 0.0 | 0.0 | 1.4 | 1.2 | 1.4 | 3.3 | 4.1 | 6.4 | 7.5 | 7.6 | 10.3 | 11.7 | 12.4 | 13.3 | 14.7 | 14.6 | 16.7 | 14.1 |
| LABA/ICS | 7.0 | 8.2 | 11.8 | 13.4 | 14.4 | 14.6 | 21.7 | 17.7 | 17.7 | 17.7 | 19.3 | 15.5 | 18.3 | 15.6 | 15.0 | 15.6 | 15.2 | 12.0 | 10.9 | 11.5 |
| LABA/LAMA | 0.0 | 0.0 | 0.0 | 0.0 | 0.4 | 0.0 | 0.2 | 0.2 | 0.2 | 0.4 | 0.6 | 0.0 | 0.2 | 0.6 | 0.6 | 0.3 | 0.8 | 5.1 | 7.5 | 7.5 |
| LAMA/ICS | 0.0 | 0.0 | 0.0 | 0.4 | 1.1 | 0.2 | 1.2 | 0.2 | 2.4 | 2.6 | 1.5 | 3.0 | 2.7 | 2.2 | 2.6 | 2.3 | 2.8 | 3.1 | 3.0 | 1.9 |
| LABA/LAMA/ICS | 0.0 | 0.0 | 0.0 | 0.0 | 1.8 | 1.7 | 3.1 | 2.6 | 4.3 | 6.0 | 7.9 | 10.6 | 9.8 | 10.7 | 11.5 | 8.9 | 8.7 | 10.7 | 11.4 | 10.1 |
| **Median time to start of new therapy post exacerbation (days)** | 1729 | 1602 | 1524 | 1076 | 1064 | 770 | 701 | 677 | 513 | 344 | 278 | 147 | 113 | 246 | 291 | 231 | 504 | 176 | 350 | 489 |
| **N** | 56 | 46 | 74 | 76 | 119 | 179 | 183 | 208 | 237 | 274 | 312 | 322 | 394 | 409 | 369 | 421 | 359 | 450 | 483 | 468 |
| **Medication review‡** | 1.8 | 2.2 | 6.8 | 15.8 | 37.8 | 59.2 | 61.7 | 66.8 | 67.9 | 67.5 | 61.9 | 67.4 | 68.3 | 60.6 | 60.7 | 59.1 | 59.9 | 58.0 | 53.6 | 58.3 |
| **N** | 17 | 21 | 16 | 25 | 44 | 76 | 89 | 72 | 108 | 131 | 198 | 188 | 220 | 217 | 237 | 252 | 240 | 261 | 300 | 299 |
| **Pulmonary rehabilitation¶** | 0.0 | 0.0 | 0.0 | 0.0 | 0.0 | 1.3 | 2.2 | 6.9 | 2.8 | 3.1 | 4.5 | 5.9 | 11.4 | 21.7 | 20.7 | 23.8 | 21.7 | 30.7 | 24.3 | 37.8 |

* assessed in the 12-month period before 1^st^ January; †12-month either side of COPD diagnosis; ‡ COPD medication review within 6 months of treatment change; ¶patients with mMRC ≥2 either side of 1^st^ January each year offered or referred for pulmonary rehabilitation within 12 months of mMRC score; § Pre-2007 we searched for any coding of cardiac risk, including Framingham score, Joint British Societies cardiac risk as well as additional evidence of cardiac risk assessments. The data were dominated by QRISK post 2007.

CAT: COPD assessment test; CONQUEST: The COllaboratioN on QUality improvement initiative for achieving Excellence in STandards of COPD care; COPD: chronic obstructive pulmonary disease; ICS: inhaled corticosteroid; LABA: long-acting β_2_-agonist; LAMA: long-acting muscarinic antagonist; mMRC: modified Medical Research Council;

S-Table 17: Percentage of high-risk **already diagnosed patients with COPD** who met relevant CONQUEST quality standards from 2000-2019 (based on specific definition of exacerbations)

| Year | 2000 | 2001 | 2002 | 2003 | 2004 | 2005 | 2006 | 2007 | 2008 | 2009 | 2010 | 2011 | 2012 | 2013 | 2014 | 2015 | 2016 | 2017 | 2018 | 2019 |
| --- | --- | --- | --- | --- | --- | --- | --- | --- | --- | --- | --- | --- | --- | --- | --- | --- | --- | --- | --- | --- |
| N | 1360 | 1594 | 2147 | 2770 | 3265 | 3434 | 4005 | 4591 | 5129 | 5613 | 6116 | 6820 | 7425 | 7851 | 8170 | 8647 | 9200 | 9982 | 10343 | 10636 |
| **Spirometry*** | 6.9 | 7.3 | 8.0 | 11.3 | 18.7 | 49.3 | 51.1 | 62.3 | 64.4 | 66.4 | 67.4 | 68.8 | 69.2 | 68.9 | 71.0 | 69.9 | 70.6 | 69.1 | 67.6 | 66.8 |
| **Spirometry†** | 14.4 | 18.4 | 22.6 | 31.7 | 59.8 | 75.7 | 77.5 | 83.5 | 83.2 | 83.7 | 84.6 | 85.8 | 85.5 | 84.8 | 84.9 | 84.5 | 84.0 | 83.4 | 82.7 | 80.4 |
| **Exacerbation history†** | 0.0 | 0.0 | 0.0 | 0.0 | 0.0 | 0.4 | 2.5 | 5.8 | 9.2 | 17.4 | 30.3 | 40.9 | 47.3 | 51.0 | 52.8 | 56.2 | 59.5 | 60.6 | 64.6 | 67.5 |
| **Cardiac risk†§** | 1.1 | 2.3 | 4.8 | 6.8 | 7.7 | 7.3 | 8.0 | 7.8 | 7.1 | 13.6 | 18.2 | 16.1 | 17.1 | 18.7 | 18.5 | 18.6 | 19.2 | 17.7 | 18.3 | 19.1 |
| **mMRC†** | 28.3 | 27.5 | 29.6 | 31.8 | 35.4 | 44.0 | 47.4 | 53.3 | 59.1 | 80.6 | 91.2 | 92.4 | 93.1 | 92.9 | 93.4 | 92.9 | 93.6 | 93.5 | 94.1 | 93.4 |
| **Smoking status*** | 23.5 | 27.7 | 33.3 | 37.5 | 52.6 | 85.5 | 86.4 | 87.7 | 88.5 | 89.0 | 87.3 | 85.7 | 87.0 | 87.3 | 89.4 | 88.3 | 89.3 | 87.9 | 88.1 | 88.7 |
| **Pn vaccination‡** | 46.9 | 45.1 | 48.0 | 47.3 | 47.2 | 49.8 | 55.4 | 55.2 | 56.9 | 56.6 | 56.7 | 56.1 | 54.1 | 56.0 | 56.0 | 55.4 | 55.5 | 55.2 | 55.4 | 54.2 |
| **Flu vaccination†** | 56.8 | 58.0 | 59.8 | 58.0 | 60.1 | 61.3 | 62.0 | 60.0 | 60.5 | 62.6 | 63.2 | 61.6 | 62.8 | 63.3 | 64.8 | 69.0 | 74.5 | 76.6 | 74.0 | 72.5 |
| **Treatment*** |  |  |  |  |  |  |  |  |  |  |  |  |  |  |  |  |  |  |  |  |
| None | 15.0 | 12.2 | 10.9 | 8.3 | 7.7 | 7.9 | 6.3 | 6.1 | 5.7 | 5.0 | 4.8 | 4.5 | 4.4 | 4.5 | 4.5 | 4.9 | 5.4 | 4.9 | 4.7 | 5.1 |
| Reliever only | 12.5 | 13.1 | 13.1 | 12.2 | 11.5 | 10.5 | 9.4 | 9.4 | 8.1 | 8.1 | 8.0 | 6.5 | 5.8 | 5.4 | 5.2 | 5.1 | 4.7 | 4.6 | 4.5 | 4.9 |
| Theophylline | 3.2 | 2.6 | 1.9 | 1.4 | 1.3 | 1.1 | 0.6 | 0.6 | 0.6 | 0.3 | 0.3 | 0.2 | 0.2 | 0.1 | 0.1 | 0.0 | 0.0 | 0.1 | 0.1 | 0.0 |
| ICS | 48.5 | 42.7 | 39.0 | 35.2 | 27.5 | 19.1 | 16.3 | 11.5 | 9.8 | 8.3 | 6.2 | 4.7 | 4.2 | 3.0 | 2.9 | 2.6 | 2.0 | 1.7 | 1.6 | 1.1 |
| LABA | 2.6 | 3.6 | 4.3 | 4.5 | 4.1 | 3.6 | 3.0 | 2.5 | 1.8 | 1.6 | 1.7 | 1.2 | 1.3 | 1.3 | 1.4 | 1.3 | 1.4 | 1.0 | 0.9 | 1.0 |
| LAMA | 0.0 | 0.0 | 0.0 | 0.2 | 1.2 | 1.4 | 1.6 | 1.8 | 2.6 | 3.7 | 4.3 | 5.3 | 6.1 | 6.8 | 7.3 | 7.4 | 7.2 | 7.5 | 7.9 | 7.5 |
| LABA/ICS | 18.0 | 25.5 | 30.7 | 36.1 | 35.9 | 37.4 | 37.5 | 36.9 | 33.8 | 27.7 | 24.5 | 21.3 | 18.9 | 18.9 | 16.3 | 16.0 | 15.1 | 13.4 | 12.8 | 11.5 |
| LABA/LAMA | 0.0 | 0.0 | 0.0 | 0.2 | 0.7 | 1.0 | 0.8 | 0.9 | 0.9 | 1.3 | 1.5 | 1.4 | 1.7 | 1.5 | 1.9 | 2.0 | 3.6 | 4.9 | 8.1 | 10.2 |
| LAMA/ICS | 0.0 | 0.0 | 0.0 | 0.3 | 1.9 | 2.4 | 2.4 | 2.5 | 2.5 | 2.7 | 2.3 | 2.7 | 2.4 | 2.1 | 1.8 | 1.7 | 1.2 | 1.2 | 0.9 | 0.6 |
| LABA/LAMA/ICS | 0.0 | 0.0 | 0.0 | 1.3 | 8.0 | 15.4 | 22.0 | 27.7 | 33.9 | 41.0 | 46.5 | 52.0 | 55.1 | 56.4 | 58.6 | 58.8 | 59.4 | 60.7 | 58.6 | 58.0 |
| **Median time to start of new therapy post exacerbation (days)** | 947 | 793 | 695 | 526 | 396 | 408 | 431 | 406 | 385 | 425 | 333 | 339 | 340 | 388 | 384 | 369 | 297 | 275 | 170 | 129 |
| **N** | 106 | 121 | 180 | 261 | 467 | 751 | 1040 | 1332 | 1651 | 2770 | 3645 | 4151 | 4531 | 4814 | 5163 | 5463 | 5883 | 6475 | 6724 | 6880 |
| **Pulmonary rehabilitation¶** | 0.0 | 0.0 | 0.0 | 0.0 | 2.8 | 3.1 | 4.2 | 4.6 | 5.9 | 8.4 | 12.1 | 16.4 | 22.6 | 29.7 | 32.7 | 31.5 | 34.3 | 34.7 | 37.3 | 55.7 |
| **N** | 334 | 399 | 566 | 755 | 899 | 980 | 1170 | 1296 | 1417 | 1695 | 1920 | 2375 | 2661 | 2827 | 3310 | 3698 | 3927 | 4279 | 4504 | 4791 |
| **COPD review§** | 19.2 | 19.3 | 29.9 | 36.8 | 57.5 | 67.0 | 67.1 | 66.5 | 66.5 | 68.7 | 71.1 | 70.2 | 67.9 | 68.3 | 65.2 | 64.3 | 62.1 | 62.0 | 61.5 | 61.7 |

* assessed in the 12-month period before 1^st^ January; †: in the 12 months before or after 1^st^ of January; ‡ Before January 1^st^; ¶patients with mMRC ≥2 either side of 1^st^ January each year offered or referred for pulmonary rehabilitation within 12 months of mMRC score; §within 6 weeks of respiratory hospitalization; § Pre-2007 we searched for any coding of cardiac risk, including Framingham score, Joint British Societies cardiac risk as well as additional evidence of cardiac risk assessments. The data were dominated by QRISK post 2007.

CONQUEST: The COllaboratioN on QUality improvement initiative for achieving Excellence in STandards of COPD care; COPD: chronic obstructive pulmonary disease; FLU: influenzae; ICS: inhaled corticosteroid; LABA: long-acting β_2_-agonist; LAMA: long-acting muscarinic antagonist; mMRC: modified Medical Research Council; PN: pneumococcal

S-Table 18: Percentage of high-risk **undiagnosed patients with COPD** who met relevant CONQUEST quality standards from 2000-2019 (based on specific definition of exacerbations)

| Year | 2000 | 2001 | 2002 | 2003 | 2004 | 2005 | 2006 | 2007 | 2008 | 2009 | 2010 | 2011 | 2012 | 2013 | 2014 | 2015 | 2016 | 2017 | 2018 | 2019 |
| --- | --- | --- | --- | --- | --- | --- | --- | --- | --- | --- | --- | --- | --- | --- | --- | --- | --- | --- | --- | --- |
| N | 1590 | 1777 | 2333 | 2861 | 3378 | 3534 | 3746 | 3986 | 4614 | 4902 | 4701 | 5274 | 5216 | 5872 | 5577 | 6131 | 6462 | 7711 | 8592 | 10095 |
| **New diagnosis*** | 4.5 | 4.8 | 3.5 | 4.5 | 4.8 | 4.9 | 4.6 | 5.1 | 4.5 | 4.5 | 4.4 | 5.6 | 5.4 | 4.4 | 4.7 | 4.7 | 4.6 | 4.2 | 3.9 | 3.6 |
| **Spirometry†** | 0.8 | 1.6 | 1.3 | 1.9 | 2.7 | 6.9 | 7.5 | 7.4 | 8.8 | 8.9 | 8.6 | 8.7 | 9.3 | 9.4 | 9.2 | 9.2 | 8.5 | 8.0 | 7.5 | 6.0 |
| **Spirometry¶** | 4.5 | 5.3 | 5.8 | 8.5 | 13.8 | 17.8 | 16.4 | 18.0 | 18.4 | 18.8 | 18.0 | 20.0 | 20.7 | 19.8 | 19.7 | 19.1 | 17.8 | 18.2 | 15.5 | 13.5 |
| **Smoking status†** | 30.0 | 35.2 | 42.5 | 45.8 | 56.4 | 78.9 | 75.8 | 80.3 | 80.6 | 81.3 | 80.0 | 77.1 | 77.2 | 77.8 | 79.1 | 74.9 | 72.5 | 70.7 | 68.6 | 67.0 |
| **Treatment†** |  |  |  |  |  |  |  |  |  |  |  |  |  |  |  |  |  |  |  |  |
| None | 70.8 | 69.2 | 66.1 | 65.8 | 62.9 | 68.7 | 69.4 | 69.7 | 68.1 | 67.5 | 68.6 | 65.5 | 66.4 | 67.5 | 67.9 | 68.2 | 69.7 | 71.1 | 74.3 | 76.0 |
| Reliever only | 12.2 | 11.5 | 13.5 | 13.9 | 16.1 | 15.4 | 16.3 | 16.1 | 16.4 | 17.5 | 16.4 | 19.9 | 18.7 | 18.5 | 18.6 | 17.7 | 17.2 | 16.9 | 14.2 | 13.5 |
| Theophylline | 0.3 | 0.5 | 0.5 | 0.5 | 0.4 | 0.2 | 0.1 | 0.1 | 0.1 | 0.1 | 0.1 | 0.1 | 0.1 | 0.0 | 0.1 | 0.0 | 0.1 | 0.0 | 0.0 | 0.0 |
| ICS | 13.5 | 15.0 | 15.6 | 14.6 | 14.3 | 10.6 | 9.4 | 8.5 | 9.4 | 8.3 | 7.4 | 7.8 | 7.2 | 6.9 | 6.6 | 6.8 | 5.8 | 5.2 | 4.9 | 4.2 |
| LABA | 0.3 | 0.1 | 0.3 | 0.3 | 0.6 | 0.4 | 0.3 | 0.2 | 0.2 | 0.4 | 0.0 | 0.1 | 0.1 | 0.2 | 0.1 | 0.1 | 0.1 | 0.1 | 0.2 | 0.1 |
| LAMA | 0.0 | 0.0 | 0.0 | 0.0 | 0.1 | 0.1 | 0.1 | 0.2 | 0.2 | 0.3 | 0.4 | 0.4 | 0.7 | 0.7 | 0.7 | 0.8 | 0.8 | 0.8 | 0.9 | 0.8 |
| LABA/ICS | 2.7 | 3.6 | 4.0 | 4.7 | 5.4 | 4.4 | 4.0 | 4.6 | 4.8 | 5.1 | 6.2 | 5.4 | 5.5 | 5.2 | 4.9 | 5.1 | 4.9 | 4.3 | 4.1 | 3.9 |
| LABA/LAMA | 0.0 | 0.0 | 0.0 | 0.0 | 0.0 | 0.0 | 0.0 | 0.0 | 0.0 | 0.0 | 0.0 | 0.0 | 0.0 | 0.1 | 0.0 | 0.0 | 0.1 | 0.2 | 0.2 | 0.3 |
| LAMA/ICS | 0.0 | 0.0 | 0.0 | 0.0 | 0.1 | 0.0 | 0.0 | 0.1 | 0.2 | 0.2 | 0.2 | 0.2 | 0.2 | 0.2 | 0.1 | 0.2 | 0.2 | 0.1 | 0.2 | 0.1 |
| LABA/LAMA/ICS | 0.0 | 0.0 | 0.0 | 0.0 | 0.1 | 0.1 | 0.2 | 0.3 | 0.4 | 0.6 | 0.6 | 0.6 | 1.0 | 0.8 | 0.9 | 0.9 | 1.0 | 1.0 | 0.9 | 0.9 |
| N | 314 | 295 | 426 | 523 | 667 | 694 | 796 | 809 | 931 | 1093 | 1060 | 1263 | 1408 | 1578 | 1811 | 2144 | 2397 | 2633 | 3077 | 3735 |
| **COPD review‡** | 9.2 | 9.5 | 14.6 | 19.9 | 39.3 | 46.3 | 48.9 | 48.5 | 43.3 | 48.0 | 48.3 | 47.5 | 48.2 | 47.0 | 43.3 | 43.8 | 43.1 | 42.0 | 39.5 | 38.2 |
| N | 1590 | 1777 | 2333 | 2861 | 3378 | 3534 | 3746 | 3986 | 4614 | 4902 | 4701 | 5274 | 5216 | 5872 | 5577 | 6131 | 6462 | 7711 | 8592 | 10095 |
| **Cardiac risk¶§** | 0.8 | 3.0 | 6.3 | 8.2 | 9.1 | 9.3 | 9.6 | 8.9 | 9.7 | 18.6 | 23.2 | 21.6 | 20.6 | 23.2 | 20.7 | 21.1 | 21.3 | 20.5 | 21.2 | 20.5 |
| **mMRC¶** | 13.9 | 14.2 | 15.9 | 17.6 | 17.4 | 19.4 | 17.9 | 18.9 | 18.6 | 19.4 | 20.9 | 21.1 | 21.1 | 20.1 | 19.8 | 20.6 | 20.5 | 19.7 | 19.8 | 18.1 |

* in the 12-month period after January 1^st^; †in the 12-month period before January 1^st^; ‡within 6 weeks post respiratory hospitalization; ¶ within 12 months either side of January 1^st^ of each year; § Pre-2007 we searched for any coding of cardiac risk, including Framingham score, Joint British Societies cardiac risk as well as additional evidence of cardiac risk assessments. The data were dominated by QRISK post 2007. CONQUEST: The COllaboratioN on QUality improvement initiative for achieving Excellence in STandards of COPD care; COPD: chronic obstructive pulmonary disease; ICS: inhaled corticosteroid; LABA: long-acting β_2_-agonist; LAMA: long-acting muscarinic antagonist; mMRC: modified Medical Research Council

**REFERENCES**

1 Payne RA, Mendonca SC, Elliott MN, *et al.* Development and validation of the Cambridge Multimorbidity Score. *CMAJ* 2020; **192**: E107–14.
