# Supplementary material for: Identification of key opportunities for optimising the management of high-risk COPD patients in the UK using the CONQUEST quality standards: an observational longitudinal study
Source: Lancet Reg Health Eur. 2023 Apr 21;29:100619. doi: 10.1016/j.lanepe.2023.100619 (PMC10149261; doi:10.1016/j.lanepe.2023.100619)
Supplement: Caption for supplementary material [file mmc11.docx]

1. Methods
2. S-Table 1: Definitions
3. S-Figure 1: Study design
4. S-Figure 2: Timeline for outcome assessment relative to index date
   1. A: Newly diagnosed cohort
   2. B: Already diagnosed cohort
   3. C: Potential undiagnosed cohort
5. Data based on sensitive definition of exacerbations
6. S-Figure 3A: Median time to initiation of new therapy after an exacerbation (newly diagnosed cohort)
7. Figure 3B: Median time to initiation of new therapy after an exacerbation (already diagnosed cohort)
8. Results
9. S-Table 2 Eligible and high-risk patient numbers by year
10. S-Table 3: High risk patient characteristics (2000-2004)
11. S-Table 4: Patient characteristics (2005-2009)
12. S-Table 5: Patient characteristics (2010-2014)
13. S-Table 6: Patient characteristics (2015-2019)
14. S-Table 7: Percentage of high-risk newly diagnosed patients with COPD who met relevant CONQUEST quality standards from 2009-2019
15. S-Table 8: Percentage of high-risk already diagnosed patients with COPD who met relevant CONQUEST quality standards from 2009-2019
16. S-Table 9: Percentage of high-risk undiagnosed patients with COPD who met relevant CONQUEST quality standards from 2009-2019
17. Data based on specific definition of exacerbations requiring a respiratory-related code within 3 days of a steroid/antibiotic prescription
18. S-Figure 4A: Median time to initiation of new therapy after an exacerbation (newly diagnosed cohort - based on specific definition of exacerbations)
19. Figure 4B: Median time to initiation of new therapy after an exacerbation (already diagnosed cohort - based on specific definition of exacerbations)
20. Results
21. S-Table 10 Eligible and high-risk patient numbers by year (based on specific definition of exacerbations)
22. S-Table 11: High risk patient characteristics (2000-2004) (based on specific definition of exacerbations)
23. S-Table 12: Patient characteristics (2005-2009) (based on specific definition of exacerbations)
24. S-Table 13: Patient characteristics (2010-2014) (based on specific definition of exacerbations)
25. S-Table 14: Patient characteristics (2015-2019) (based on specific definition of exacerbations)
26. S-Table 15: Percentage of high-risk newly diagnosed patients with COPD who met relevant CONQUEST quality standards from 2009-2019 (based on specific definition of exacerbations)
27. S-Table 16: Percentage of high-risk already diagnosed patients with COPD who met relevant CONQUEST quality standards from 2009-2019 (based on specific definition of exacerbations)
28. S-Table 17: Percentage of high-risk undiagnosed patients with COPD who met relevant CONQUEST quality standards from 2009-2019 (based on specific definition of exacerbations)
